# Origins of High-Activity Cage-Catalyzed Michael Addition

**DOI:** 10.1021/jacs.4c05160

**Published:** 2024-07-08

**Authors:** Patrick J. Boaler, Tomasz K. Piskorz, Laura E. Bickerton, Jianzhu Wang, Fernanda Duarte, Guy C. Lloyd-Jones, Paul J. Lusby

**Affiliations:** † EaStCHEM School of Chemistry, University of Edinburgh, Joseph Black Building, David Brewster Road, Edinburgh, Scotland EH9 3FJ, U.K.; ‡ Chemistry Research Laboratory, University of Oxford, Oxford OX1 3TA, U.K.

## Abstract

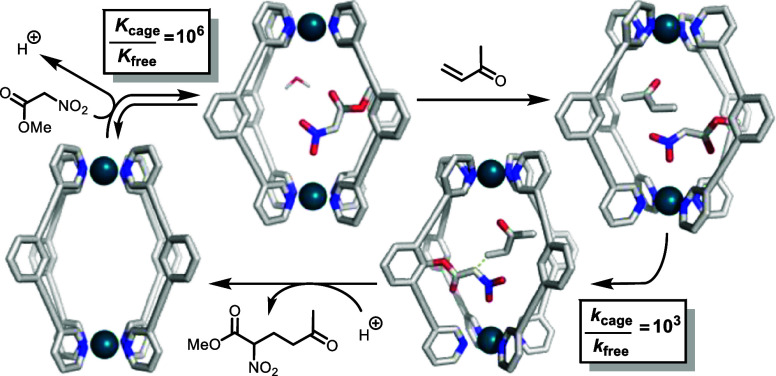

Cage catalysis continues to create significant interest, yet catalyst function remains poorly understood. Herein, we report mechanistic insights into coordination-cage-catalyzed Michael addition using kinetic and computational methods. The study has been enabled by the detection of identifiable catalyst intermediates, which allow the evolution of different cage species to be monitored and modeled alongside reactants and products. The investigations show that the overall acceleration results from two distinct effects. First, the cage reaction shows a thousand-fold increase in the rate constant for the turnover-limiting C–C bond-forming step compared to a reference state. Computational modeling and experimental analysis of activation parameters indicate that this stems from a significant reduction in entropy, suggesting substrate coencapsulation. Second, the cage markedly acidifies the bound pronucleophile, shifting this equilibrium by up to 6 orders of magnitude. The combination of these two factors results in accelerations up to 10^9^ relative to bulk-phase reference reactions. We also show that the catalyst can fundamentally alter the reaction mechanism, leading to intermediates and products that are not observable outside of the cage. Collectively, the results show that cage catalysis can proceed with very high activity and unique selectivity by harnessing a series of individually weak noncovalent interactions.

## 1 Introduction

The development of bioinspired synthetic catalysts that use noncovalent interactions to encapsulate substrates and promote their transformation into products has remained a significant academic challenge for more than 70 years.^1^ During this time, several types of artificial receptors have been explored, from cyclodextrins,^2,3^ other covalent macrocycles^4,5^ and three-dimensional structures,^6,7^ to self-assembled systems that rely on either hydrogen bonding^8−10^ or metal–ligand interactions.^11−22^ Pioneering studies of coordination cages by Fujita,^23^ Stang,^24^ Raymond,^25^ and others^26−34^ means that it is now relatively easy to design and prepare structures that possess well-defined cavities. These metallo-organic systems can be exploited in much the same way that earlier supramolecular catalysts were, such as by increasing the effective concentration of two substrates^35,36^ or by binding a single substrate in a constricted orientation.^37−39^

Coordination cages also possess a catalytically useful property that is absent in many other host systems: they are invariably charged. It has now been shown several times that this charge can be used to promote complementary reactions;^11,12,14,38−41^ however, the precise origin(s) of the rate enhancement are often not well understood. For example, ionogenic reactions often proceed via several charged intermediates, before or after the rate-determining step. How the cage interacts with these different species can have a significant impact on the overall acceleration. Furthermore, the charge can also be exploited to concentrate nonencapsulated anions or cations at the periphery, producing higher effective molarity.^15,42^ Deciphering how these effects impact or are responsible for encapsulated catalysis will significantly aid in the expansion beyond the relatively few cage structures that currently dominate the field.

Probing the mechanisms of coordination cage catalysis, and indeed other forms of reactivity that rely on weak noncovalent interactions,^43−48^ presents significant challenges. Specifically, the relatively weak binding to substrates, intermediates, and products can cause difficulties in quantifying catalyst speciation. For example, ^1^H NMR spectra of cage-catalyzed reactions frequently present a single set of time-averaged cage signals, with chemical shifts that temporally evolve to reflect the concentrations of various host–guest complexes. This lack of detailed information on the catalyst speciation limits studies to analyzing the consumption of substrates and the evolution of products. The data from these experiments has typically been processed using linearized forms of the Michaelis–Menten equation, such as Lineweaver–Burk or Eadie-Hofstee plots.^40,49^ Host–guest titrations,^14−16^ isotopic labeling,^50^ variable-temperature experiments,^37^ structure–activity relationships,^51^ and several computational studies^52−58^ have also been used to shed further light on catalysis. A full kinetic simulation of a cage-catalyzed multistep process requires the quantification of most or all species; otherwise, there are a range of possible solutions, leading to uncertainty in some, or all, of the extracted parameters. Herein, we report the analysis of coordination cage-catalyzed reactions using numerical-method-based kinetic simulations, underpinned by the observation of detectable intermediate complexes. Combining this approach with computational and variable-temperature kinetic studies has revealed the dual origins of the very high activity of the cage.^38^

## 2 Results and Discussion

### 2.1 In Situ ^1^H NMR Spectroscopic Monitoring of Cage-Catalyzed Michael Reactions

**Scheme 1:**
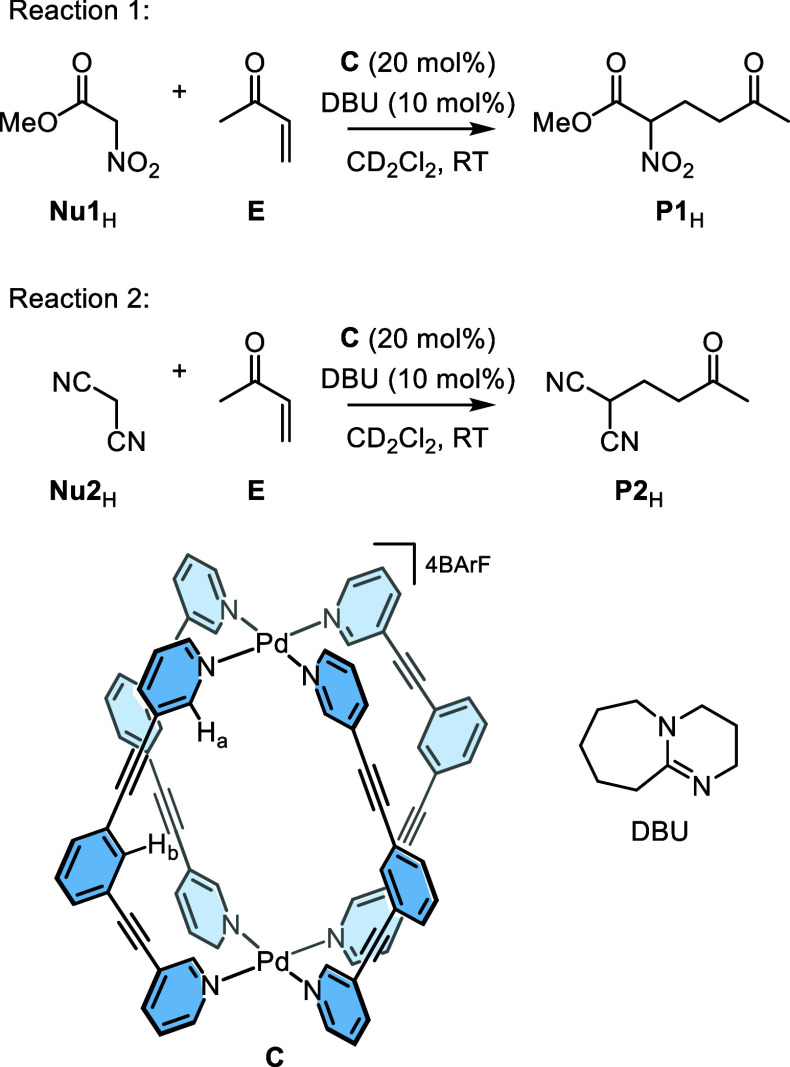
Cage (**C**)-Catalyzed Michael Addition Reactions of Electrophile **E** with Pronucleophiles **Nu1**_H_ and **Nu2**_H_

The dinuclear Pd-cage **C** (Scheme 1) catalyzes the Michael addition of pronucleophiles and electrophiles.^41^ We focus our mechanistic investigation on two examples of this reaction, using the same electrophile, **E**, with different pronucleophiles, **Nu1**_H_ and **Nu2**_H_ (Scheme 1, reactions 1 and 2, respectively). These transformations were selected because the pronucleophiles possess different intrinsic properties: **Nu1**_H_ (p*K*_a_ = 5.7) is significantly more acidic than **Nu2**_H_ (p*K*_a_ = 11), while conversely, the Mayr-Patz reactivity index predicts that [**Nu2**]^−^ is four orders more reactive than [**Nu1**]^−^.^59^ It was envisaged that a comparison of these two reactions would address a key question as to whether activity stems from pronucleophile acidification through stabilization of the conjugate anion inside the 4+ coordination structure, modulated reactivity of the bound nucleophilic anion, or both effects combined.

We have previously found that the acceleration provided by **C** is significantly enhanced using either 18-crown-6 or an organic base, such as 1,8-diazabicyclo (5.4.0)undec-7-ene (DBU).^41^ These additives stabilize proton loss from the pronucleophile in the case of 18-crown-6 by binding the hydronium ion that results from the reaction with residual water. Herein, we focus on the use of DBU which induces detectable background Michael addition in the absence of cage **C**. These base-catalyzed background reactions (*k*^B^) usefully allow comparison with the rate of the cage-catalyzed reactions (*k*^C^) and thus a quantitative estimation of the acceleration provided by the cage over a reference “bulk phase” process. In contrast, 18-crown-6 does not induce any detectable background reaction but does interact with the exterior of the cage, thus complicating analysis.

**Figure 1:**
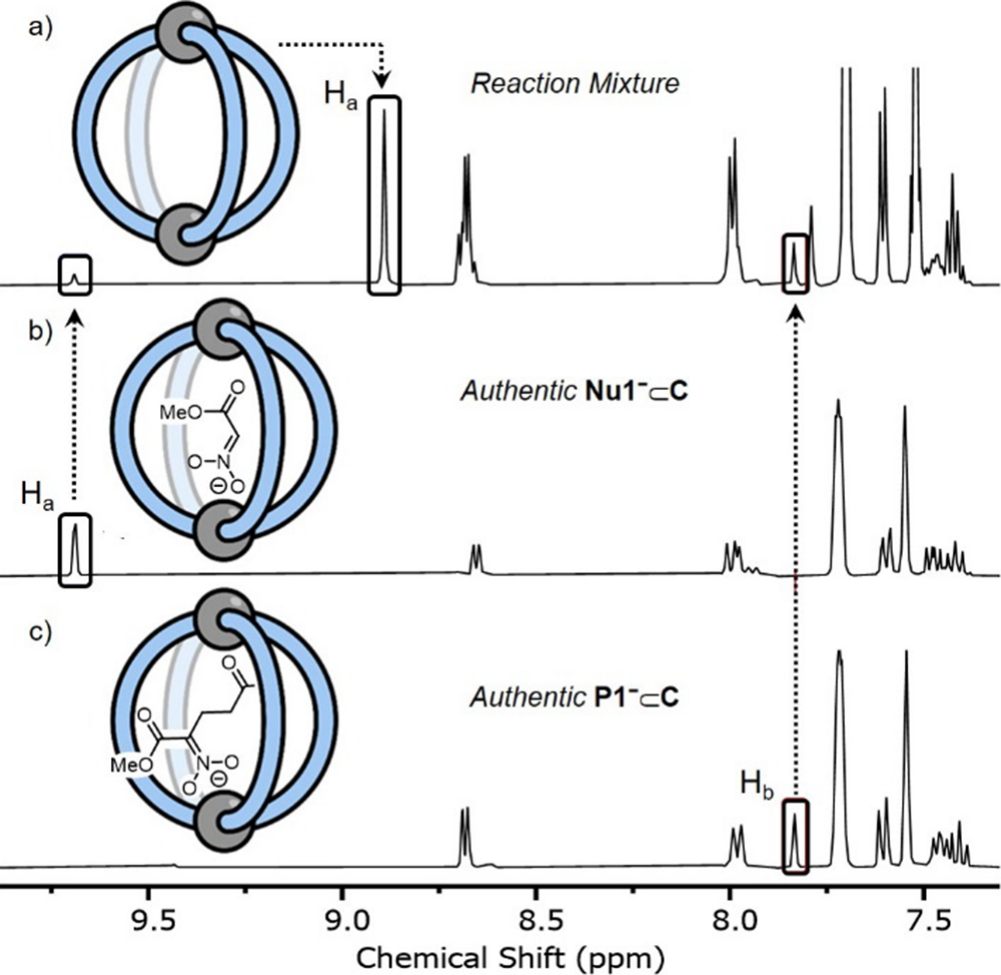
^1^H NMR spectra (600 MHz, CD_2_Cl_2_, 298 K) of (a) reaction 1 immediately following initiation; (b) authentically generated **Nu1**^–^⊂**C** (by the reaction of **C**, **Nu1**_H_, and DBU); and (c) authentically generated **P1**^–^⊂**C** (by the reaction of **C**, **P1**_H_, and DBU). The identities of protons H_a_ and H_b_ in cage **C** are shown in Scheme 1.

The in situ ^1^H NMR spectroscopic analysis^60^ of reactions 1 and 2 reveals the presence of multiple, low-intensity signals (Figures 1, S2, S7, S14, and S21). These can be readily attributed to different catalytic-cage intermediates because these resonances are characteristic of the H_a_ and H_b_ protons; these are the inward-facing hydrogen atoms that are sensitive to bound guests (Scheme 1). Significant shifting of the H_a_ signal to >9.5 ppm is indicative of a strong guest binding,^61^ consistent with a rate of exchange that is slower than the ^1^H NMR time scale. These low-intensity signals were assigned to specific intermediates by comparison with the authentically generated samples. For example, in reaction 1, three sets of signals are observed: (i) cage containing only weak binding guests (**Nu1**_H_, *K*_a_ ≈ 30 M^–1^; **P1**_H_, *K*_a_ ≈ 150 M^–1^,^41^ or solvent) collectively referred to herein as “empty” cage **C** (Figure 1a); (ii) the cage containing the nucleophilic anion **Nu1**^–^⊂**C** (Figure 1b), and (iii) the cage containing the anionic product **P1**^–^⊂**C** (Figure 1c).

### 2.2 Kinetics and Mechanism of Cage-Catalyzed Reaction 1

**Scheme 2:**
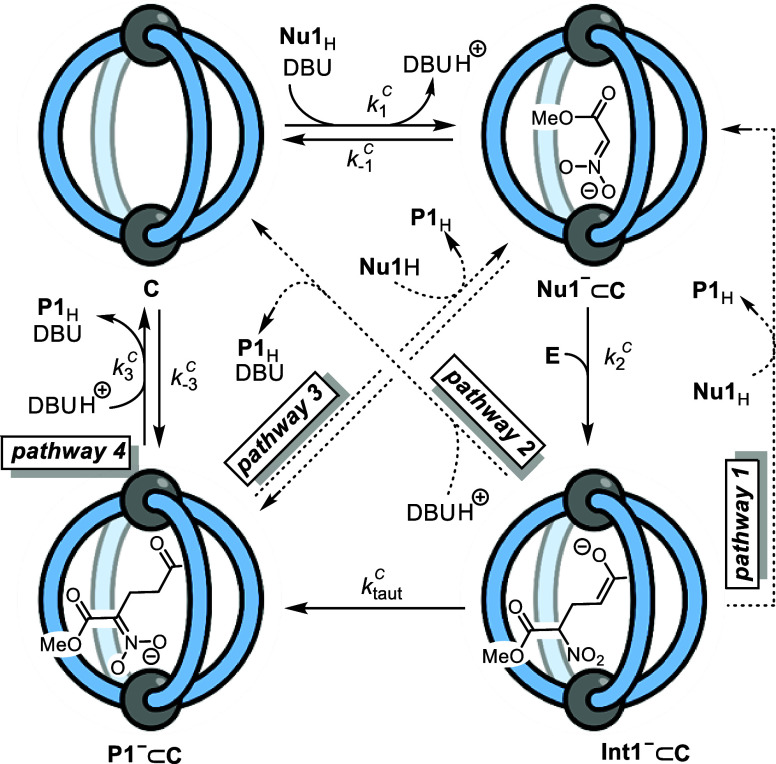
Pathways 1–4 Used for Numerical Methods Simulations of the Temporal Concentrations of **Nu1**_H_, **E**, **P1**_H_, **C**, **Nu**^–^⊂**C**, and **P1**^–^⊂**C** in Reaction 1 The data is not consistent with catalysis solely via pathway 1. The model based on pathway 4 is marginally more consistent with the experimental data than models based on pathway 2 or 3, see the Supporting Information Section S.1.

With a method to analyze both the overall kinetics of reaction 1 and the major speciation of the cage-derived intermediates, we considered a range of general mechanisms for the catalysis. Mechanisms that involve binding on the outside of the cage were eliminated from consideration by the complete inhibition of catalysis on addition of a strong-binding competitor guest.^41^ Our analysis thus started with the four pathways illustrated in Scheme 2. Since the total concentration of protonated DBU matches that of the slow-exchange cage signals, all of the deprotonated species are bound by the cage; thus, none of the models include a bulk-phase DBU-mediated reaction in the presence of **C**. Common to all four pathways is the encapsulation-deprotonation of **Nu1**_H_ to give **Nu1**^–^⊂**C**, which we simplified to a single rapid associative deprotonation equilibrium (*K*_1_^C^). From **Nu1**^–^⊂**C**, all four pathways involve C–C bond formation (*k*_2_^C^) within the cage by reaction with **E** to give **Int1**^–^⊂**C**. At this point, the four pathways diverge to generate **P1**_H_, Scheme 2.

*Pathway 1*: In this route, **Int1**^–^⊂**C** reacts irreversibly with **Nu1**_H_ via proton transfer to release **P1**_H_ and regenerate the bound anionic nucleophile, **Nu1**^–^⊂**C**. This associative endocage mechanism avoids loss in Coulombic stabilization arising from dissociation of an anion from the tetra-cationic cage.
*Pathway 2*: Here, **Int1**^–^⊂**C** undergoes dissociative exocage protonation by DBUH^+^ to give cage **C**, product **P1**_H_, and DBU. In this mechanism, the “empty” cage can nonproductively bind (*K*_–3_^C^) the DBU-deprotonated product, [**P1**]^−^[DBU_H_]^+^, resulting in 'off cycle' inhibition.
*Pathway 3:* In contrast to pathways 1 and 2, **Int1**^–^⊂**C** undergoes fast irreversible tautomerization, *k*_taut_, to give **P1**^–^⊂**C**. Product **P1**_H_ is then released by the associative proton transfer from **Nu1**_H_ to regenerate **Nu1**^–^⊂**C**. While this bypasses “empty” cage **C**, the reversible proton-transfer still results in product inhibition.
*Pathway 4:* This pathway diverges from pathway 3 at the stage of **P1**^–^⊂**C** through reversible associative proton transfer (*K*_3_^C^) from [DBU_H_]^+^. This results in catalytic turnover by pathway 4 involving all four cage species, Scheme 2.

Each of the models (pathways 1–4) were tested by numerical methods fitting to full temporal concentration data (>98% conversion) acquired under two initial sets of conditions: one with the nucleophile (**Nu1**_H_) in 3-fold excess over the electrophile (**E**) and the other with them approximately equimolar (see the Supporting Information Section S4.2). Models based on Pathway 1 were unable to satisfactorily fit the substrate and product temporal concentrations, with major deviations when the nucleophile was not in excess (Figure S43). Moreover, the model was wholly unable to reconcile the experimentally determined concentrations of the two detectable cage intermediates (**Nu**^–^⊂**C** and **P1**^–^⊂**C**, Figures S42 and S44).

Models based on pathways 2–4 gave significantly better correlations (Figures S46–S59) with all three providing the subtle but evident inhibition by **P1**_H_/**P1**^–^. There is a small but distinct improvement in the correlations using models based on pathways 2 and 4, with the latter marginally providing the best overall fit (Figures S56–S59). The optimized fitting parameters for this model are shown in Table 1. Several key observations emerge:

a) Under the conditions explored, where [**E**]_0_ = 4 mM, the C–C bond formation step (*k*_2_^C^) is turnover-rate limiting, with the tautomerization (*k*_taut_^C^) sufficiently favorable for **Int1**^–^⊂**C** not to accumulate.
b) Analysis of the cage speciation by ^1^H NMR integration shows that the deprotonation equilibria for **Nu1**_H_ and **P1**_H_ (*K*_1_^C^ and *K*_–3_^C^, respectively) are sufficiently favorable for essentially complete protonation of DBU, and thus, [**Nu1**^–^**⊂C**] + [**P1**^–^**⊂C**] = [DBU]_tot_.
c) The cage **C** has a slightly greater affinity (*K*_1_^C^/*K*_–3_^C^ < 1 Table 1) for the product anion **P1**^–^ over the deprotonated nucleophile, **Nu1**^–^. This leads to modest product inhibition in the catalysis, albeit with all the limiting reagent (**E**) eventually being fully consumed.
d) Because the proton transfer equilibria (*K*_1_^C^, *K*_3_^C^) are rapid, the models do not reveal information about their mechanisms, e.g., dissociative, associative, or intramolecular.
e) The models based on pathways 2 and 4 differ only by their route to the detected species **P1**^–^⊂**C**, and the fitting values for turnover (*k*_2_^C^) and inhibition (*K*_1_^C^/*K*_–3_^C^) are the same, within experimental error (see Supporting Information, Section S6.1).

**Table 1:** Key Parameters for Models of the Kinetics of Reaction 1 Catalyzed by C + DBU and DBU Alone^a^^,^^b^

parameter^a^	**C** + DBU	parameter	DBU^c^
*K*_1_^C^ (M^–1^)^d^	>10^4^	*K*_1_^B^ (M^–1^)	4.2 × 10^5^^e^
*k*_2_^C^ (M^–1^ s^–1^)	2.7× 10^1^	*k*_2_^B^ (M^–1^ s^–1^)	2.2 × 10^–2^^f^
*K*_1_^C^/*K*_–3_^C^	0.13		
*k*_2_^C^/*k*_–3_^C^	0.17		
*k*_taut_^C^ (s^–1^)	>1		

a These parameters provide a satisfactory fit to the temporal concentrations of **Nu1**_H_, **E**, **P1**_H_, **C**, **Nu1**^–^⊂**C**, and **P1**^–^⊂**C**, using numerical methods modeling of pathway 4, with all proton transfer equilibria set to be fast.

b For fitting thresholds, see Supporting Information, Section S6.1.4.

c From NMR titration (*K*_1_^B^) and model optimization (*k*_2_^B^), see Supporting Information, Section S8.1.

d Attempts to obtain this parameter using direct titration were limited by the instability of **C** in the presence of excess **Nu1**_H_ and DBU, see Supporting Information, Section S7.3.

e For **Nu1**_H_ + DBU ↔ [**Nu1**]^−^[DBU_H_]^+^.

f For the elementary step [**Nu1**]^−^[DBU_H_]^+^ + **E** → [**P1**]^−^[DBU_H_]^+^.

### 2.3 Kinetics of Reaction 1 Catalyzed by DBU Alone

To contextualize the kinetics of the cage catalysis, we analyzed reaction 1 catalyzed by DBU in the absence of **C**. Under these conditions, the time for complete consumption of **E** increases from about an hour to more than a month. To facilitate the in situ ^1^H NMR spectroscopic analysis of the kinetics, the reaction was conducted at 5- to 10-fold higher concentrations of all reactants. The reaction kinetics were satisfactorily simulated using a simple two-stage model comprising (i) the deprotonation equilibrium (*K*_1_^B^) between DBU and **Nu1**_H_ to give [**Nu1**]^−^[DBU_H_]^+^ and (ii) an irreversible, turnover-rate limiting (*k*_2_^B^) 1,4-addition of **Nu1**^–^DBUH^+^ to **E**, followed by rapid tautomerization and proton transfer to give **P1**_H_ and DBU. Fitting of a 1:1 binding isotherm (Figures S67 and S68) to data from a ^1^H NMR titration of DBU with **Nu1**_H_ in which the protonated and neutral forms of DBU are in fast exchange gave the equilibrium constant, *K*_1_^B^ = 4.2 × 10^5^ M^–1^. The magnitude of *k*_2_^B^ (2.2 × 10^–2^ M^–1^ s^–1^, Table 1) was then estimated by numerical fitting of the simple two-step model to experimental temporal concentration data for **P1**_H_ in reactions conducted at three different [DBU]_tot_ catalyst concentrations (Figure S79). Three orders of magnitude acceleration of the C–C bond-forming step within the cage, compared to the “bulk-phase” process, is thus evident from the ratio *k*_2_^C^/*k*_2_^B^ = 1.2 × 10^3^, independent of any changes in equilibrium between **Nu1**_H_ and [**Nu1**]^−^ exerted by the cage, i.e., *K*_1_^C^ versus *K*_1_^B^.

### 2.4 Computational Analysis of Cage-Catalyzed and Bulk-Phase DBU-Catalyzed Reaction 1

**Figure 2:**
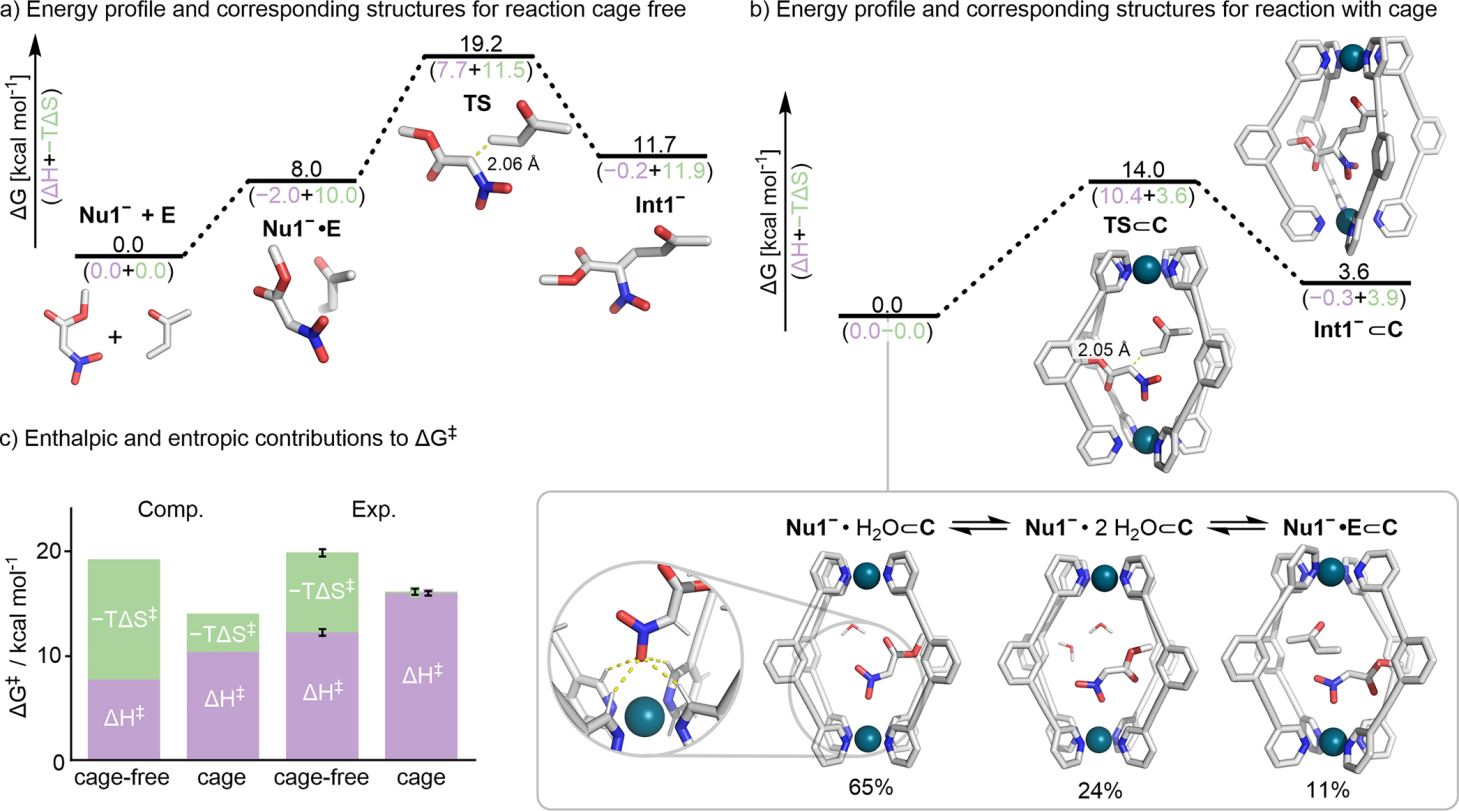
(a) Gibbs free energy and corresponding structures for the cage-free (**Nu1**^–^ + **E** → **Int1**^–^) reaction. (b) Gibbs free energy profiles and corresponding structures for the cage-mediated (**Nu1**^–^⊂**C** + **E** → **Int1**^–^⊂**C**) reaction. The ground state was calculated according to Boltzmann weighting of three states: **Nu1**^–^·H_2_O⊂**C1**, **Nu1**^–^·2H_2_O⊂**C1**, and **Nu1**^–^·**E**⊂**C1**. The magnified region shows the binding of the **Nu1**^–^ nitronate group to the H-bond donor pocket on the cage interior. (c) Comparison of the activation parameters predicted by DFT to those determined experimentally. Calculations on the CPCM(DCM)-M06-2X/def2-TZVP//CPCM(DCM)-PBE0-D3BJ/def2-SVP level of theory.

The above results pose the obvious question: why is the activation barrier within the cage more than 4 kcal mol^–1^ lower than that of the “bulk-phase” process? To better understand the origin of this acceleration, we used density functional theory (DFT) to analyze the rate-limiting step, **Nu1**^−^ + **E** → **Int1**^−^, in the presence and absence of a cage (Figure 2a,b). For the process mediated by DBU in the absence of a cage, i.e., *k*_2_^B^, the computed activation barrier (Δ*G*^‡^_uncat_ = 19.2 kcal mol^–1^) is in good agreement with experiment (Δ*G*^‡^_exp_ = 19.8 kcal mol^–1^). Approximately 40% of the calculated energetic barrier arises from bringing the substrates into complex **Nu1**^–^·**E** (Δ*G*_complex_ = 8.0 kcal mol^–1^), with significantly more than half of the total activation barrier stemming from entropic costs (−*T*Δ*S*^‡^ = 11.5 kcal mol^–1^).

The reaction pathway inside the cage, i.e., *k*_2_^C^, was elucidated through a combination of classical methods, including molecular dynamics (MD) and docking and DFT calculations at the CPCM(DCM)-M06-2X/def2-TZVP//CPCM(DCM)-PBE0-D3BJ/def2-SVP level of theory (see Supporting Information). MD simulations reveal that **Nu1**^−^ resides inside the cage for a significant period (53%), accompanied by either dichloromethane or one or two residual water molecules. **Nu1**^−^ adopts a stable binding mode involving H-bond interactions between one of the H-bond donor pockets (i.e., 4 × neighboring H_a_ atoms) on the cage interior and a single oxygen atom of the nitronate group (Figure 2b). Representative structures from MD trajectories, along with the reactant complex inside the cage, were optimized by DFT. Three structures were identified to have similar energies within 1.1 kcal mol^–1^, **Nu1**^–^·H_2_O⊂**C**, **Nu1**^–^·2H_2_O⊂**C**, and **Nu1**^–^·**E**⊂**C**, that coexist in solution with a ratio of 0.65:0.24:0.11, based on their Boltzmann-weighted distribution (Figure 2b). Consequently, the energy of the effective ground state was calculated as the Boltzmann-weighted Gibbs free energy of these three states, resulting in an activation barrier of Δ*G*^‡^_cat_ = 14.0 kcal mol^–1^. The calculated acceleration of the C–C bond-forming step inside the cage, relative to the bulk phase, thus aligns well with the experiment (ΔΔ*G*^‡^_exp_–ΔΔ*G*^‡^_comp_ = 1 kcal mol^–1^).

The favorable preassociation of the electrophile and nucleophile within the cage to give **Nu1**^–^·**E**⊂**C1**, contrasts with the high energy needed to form the reactant complex, **Nu1**^–^·**E**, in the cage-free reaction. Moreover, computational analysis indicates that the reduced activation barrier in the cage, ΔΔ*G*^‡^_comp_, stems from a significant reduction in entropy (−*T*ΔΔ*S*^‡^_comp_ = −7.9 kcal mol^–1^; Figure 2b).

These observations suggest that favorable cobinding of substrates within the cage accelerates the reaction, as found in other examples of supramolecular catalysis.^3−5,8,35^ Perhaps surprisingly, reaction inside the cage induces a slight electronic destabilization of the TS, with ΔΔ*H*^‡^_comp_ = +2.7 kcal mol^–1^, which can be attributed to the distortion needed to reach the TS geometry (Figure S101). To better understand the origin of this destabilization, we analyzed the influence of the cage on the elementary reaction **Nu1**^–^·**E → TS** using a framework analogous to distortion-interaction analysis (Figure S101).^62^ The results show that the distortion caused by steric clashes with the cage destabilizes the TS (Δ*E*^‡^_dist_ = +2.8 kcal mol^–1^), outweighing the slightly favorably interaction energy (Δ*E*^‡^_inter_ = −1.6 kcal mol^–1^).

The origin of the acceleration in the C–C bond-forming step has also been investigated experimentally by measuring the rates of reaction at different temperatures for the cage and cage-free processes. The activation parameters obtained from standard Eyring analyses of this data (see Supporting Information, Section 9) are consistent with the overall trend obtained from calculations (Figure 2c); compared to the bulk-phase process, *k*_2_^B^, the cage-catalyzed process, *k*_2_^C^, has a slightly higher enthalpy of activation (ΔΔ*H*^‡^_exp_ = +3.7 kcal mol^–1^), but this is notably outweighed by a significantly lower entropy component (*T*ΔΔ*S*^‡^_exp_ = −7.5 kcal mol^–1^ at 298 K). A mechanism in which the rate of C–C bond formation is faster due to the favorable dual encapsulation of both substrates inside the cage is therefore supported by both calculations and experiments.

### 2.5 Kinetic Analysis of Cage-Catalyzed Reaction 2 and Comparison to the Bulk-Phase Process

**Figure 3:**
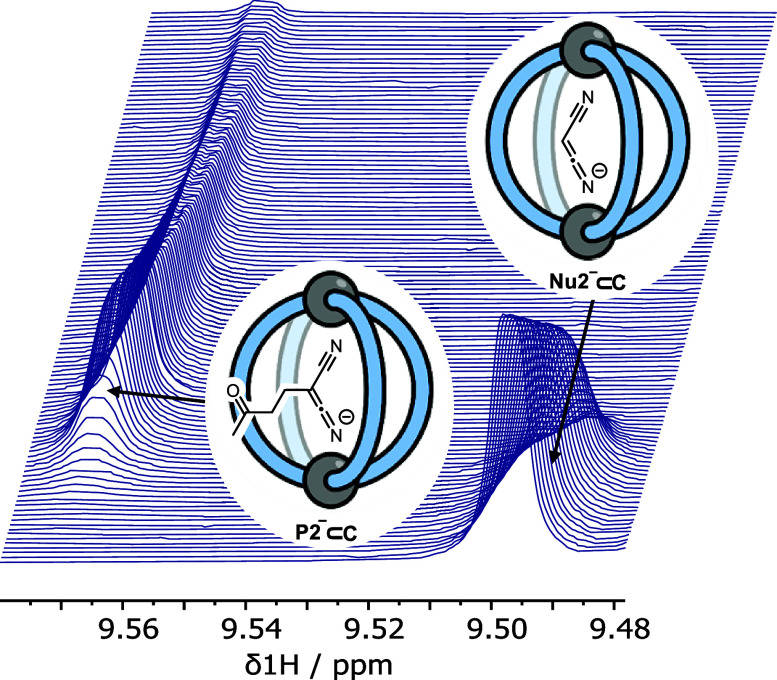
Partial ^1^H NMR spectra (600 MHz, CD_2_Cl_2_, 298 K) for reaction 2 showing the time-dependent evolution of **Nu2**^–^⊂**C** and **P2**^–^⊂**C**. The signals correspond to the H_a_ atoms of **C** (Scheme 1). The first spectrum, obtained immediately after reaction initiation, is shown at the bottom, with subsequent spectra recorded at 30 s intervals.

**Scheme 3:**
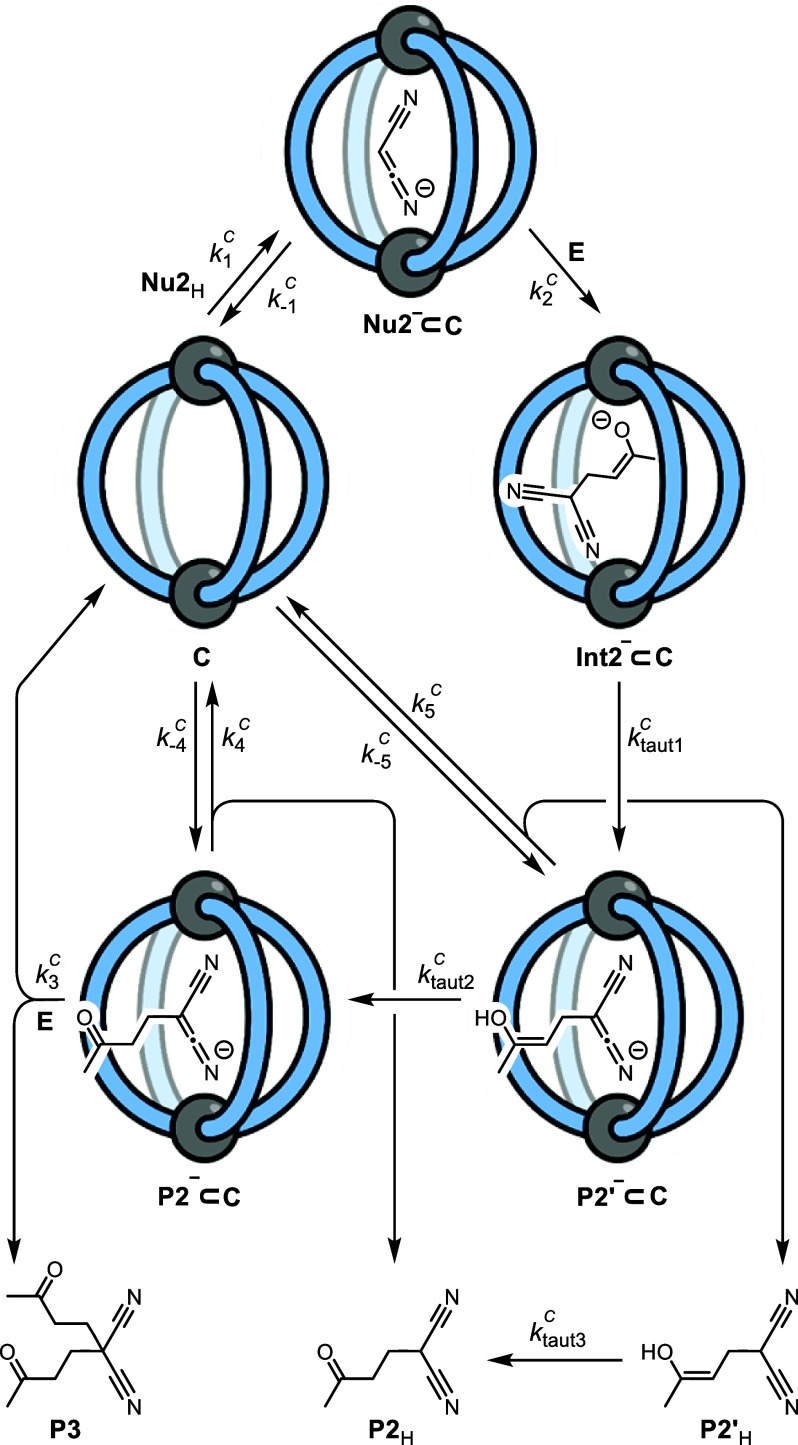
Proposed Catalytic Cycle for Reaction 2, This mechanism is supported by kinetic simulation of the temporal concentrations of **Nu2**_H_, **E**, **P2**_H_, **P3**, **C** and detectable intermediates **Nu2**^–^⊂**C**, **P2**^–^⊂**C**, and **P2′**_H_. DBU and DBUH^+^ have been omitted from the catalytic cycle for clarity, as has a decomposition step from **Int2**^–^⊂**C**, which accounts for a small amount of free ligand (<5%). For key parameter thresholds and relationships, see Table S8.

The in situ ^1^H NMR data for reaction 2 is considerably more complex than that of reaction 1 (Figures S14–S28). First, reaction 2 generates two products corresponding to single addition, **P2**_H_, and double addition, **P3**, of the electrophile, **E**. Second, a transient enol tautomer, **P2′**_H_, of the single addition product can be detected during the early stages of the reaction. In reaction 1, the lower affinity of the substrate anion, **Nu1**^−^, for the cage compared to the product, **P1**^−^ (*K*_1_^C^/*K*_–3_^C^ = 0.13, Table 1), means that the evolution of catalytic species from **Nu1**^–^⊂**C** to **P1**^–^⊂**C** occurs within the dead-time of reaction initiation (Figures S30 and S32). The concentration of **P1**^–^**⊂C** then remains approximately constant throughout the reaction. In contrast, the sequential diminishment of **Nu2**^–^⊂**C**, and the appearance of **P2**^–^⊂**C**, is readily detected during reaction 2 (Figure 3). Moreover, with the accumulation of the nonacidic double addition product, **P3**, the catalyst speciation in reaction 2 progressively reverts to being dominated by the 'empty' cage, **C**. Beginning with the optimized model for reaction 1 and incorporating the additional features noted above, we arrived, after considerable exploration of alternative possible mechanisms, at the model shown in Scheme 3.

Reaction 2 is also unusual in that enol **P2′**_H_ is not detected when the reaction is mediated by DBU alone. In the best-fit model for the cage-catalyzed process, **Int2**^–^⊂**C** undergoes tautomerization (Scheme 3, *k*_taut1_) and then the keto-product **P2**_H_ is liberated by (a) dissociative protonation (*k*_5_) and bulk-phase enol-keto tautomerism (*k*_taut3_) and (b) enol-keto tautomerism within the cage to give the bound keto-product anion, **P2**^–^⊂**C** (*k*_taut2_), followed by dissociative protonation (*k*_4_). The intermediacy of the putative complex **P2′**^–^⊂**C** is tentative and not easily tested because of the transient nature of **P2′**_H_. Nonetheless, the kinetic simulations suggest that product **P2**_H_ is predominantly liberated from **P2**^–^⊂**C** (see Supporting Information) in competition with the second addition to **E** (*k*_3_). The two reactions with electrophile **E** proceed with similar efficiency (*k*_3_ ∼ *k*_2_, Table S8) despite the increase in the steric bulk of the nucleophile.

**Figure 4:**
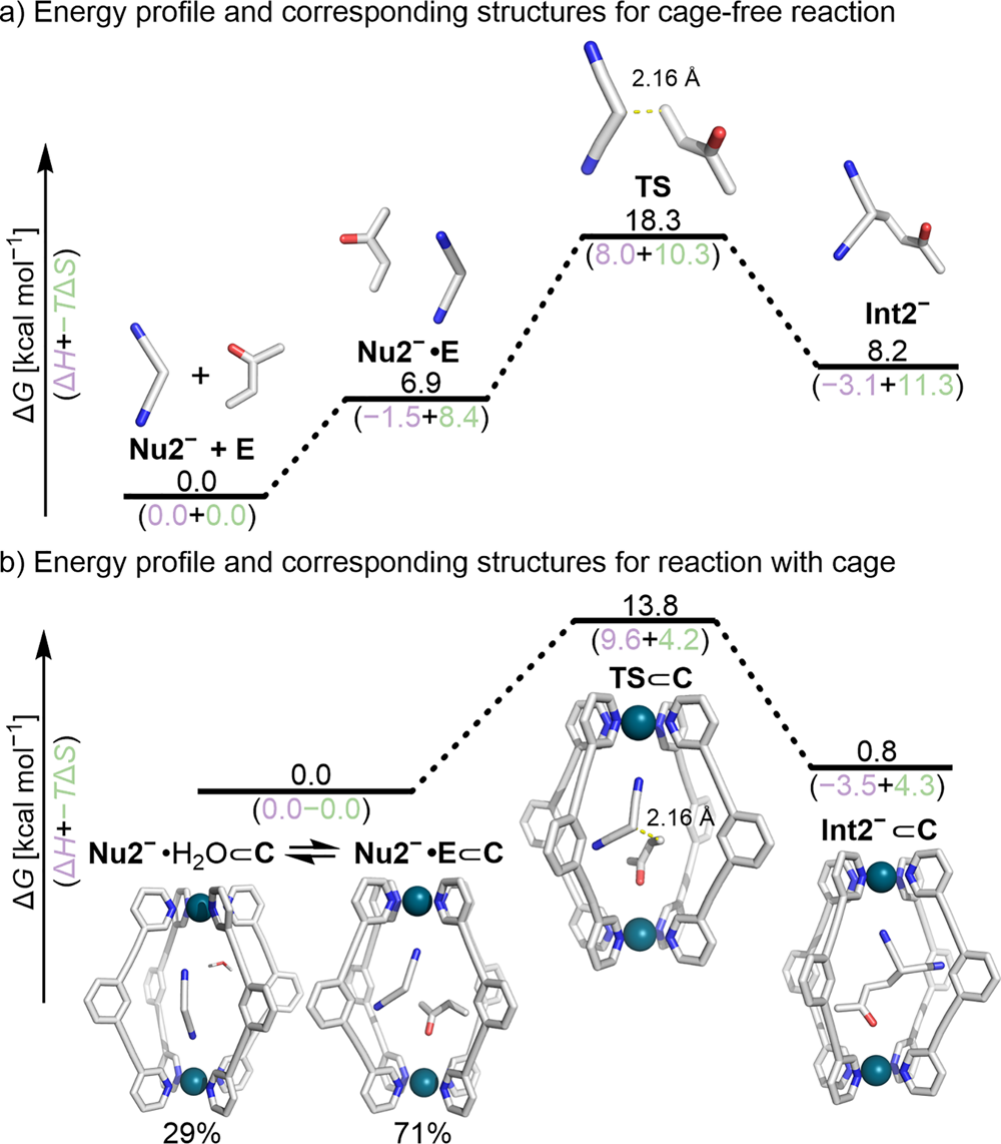
Energy profile and corresponding structures for reaction 2 (a) without and (b) with a cage. For (b), the ground state was calculated as the Boltzmann average of the **Nu2**^–^·H_2_O⊂**C**, **Nu2**^–^·**E**⊂**C** states. Calculations were performed at the CPCM(DCM)-M06-2X/def2-TZVP//CPCM(DCM)-PBE0-D3BJ/def2-SVP level of theory.

We also analyzed the kinetics of reaction 2 mediated by DBU in the absence of cage **C** (see the Supporting Information, Section 8.2). The rate of product, **P2**_H_, generation decreases disproportionately with conversion due to a second-order dependency on the electrophile concentration, [**E**]. Under some reaction conditions, this can give rise to temporal concentration profiles that are similar to those of product inhibition. For a discussion of possible mechanisms for this, see the Supporting Information, Section S8.2.2. The fundamental differences in the mechanisms of reaction 2 inside cage **C** and in the bulk phase by DBU alone hinder any meaningful direct comparison. Consequently, we evaluated the cage against the reference reaction using the same computational procedures that were applied in reaction 1. For the cage-free DBU-mediated process, a significant proportion of the activation barrier (Δ*G*^‡^ = 18.3 kcal mol^–1^, Figure 4a) stems from the entropically unfavorable generation of the reactant complex (Δ*G*_complex_ = 6.9 kcal mol^–1^;–TΔ*S*^‡^ = 10.3 kcal mol^–1^).

For the cage-catalyzed process, MD simulations in dichloromethane show that **Nu2**^−^ stays in the cage accompanied by 1–4 water molecules for a significant period (48%). In contrast to **Nu1**^−^, no clear binding mode of **Nu2**^−^ was evident (although it is clear that there are hydrogen bonding interactions between the nitrile N atom of the H_a_ protons,^63^ consistent with the NMR data). Therefore, representative structures of **Nu2**^−^ and water molecules in the cage were located by clustering MD trajectory frames (Figure S104). The most stable structure, **Nu2**^–^**·H**_**2**_**O**⊂**C**, is higher in energy than the reactant complex **Nu2**^–^**·E**⊂**C**, supporting the hypothesis that C–C bond formation proceeds via a favorable termolecular Michaelis complex. The significant difference in entropic contribution to reach the transition state without and with the cage (−*T*ΔΔ*S*^‡^_comp_ = −6.1 kcal mol^–1^) provides additional evidence for this conclusion.

### 2.6 Estimation of the Cage-Induced Acidification of the Pronucleophile

Having established that C–C bond formation, *k*_2_^C^, between **Nu1**^−^ and **E** inside the cage is accelerated relative to the bulk-phase process outside the cage, *k*_2_^B^, we examined the impact the cage has on the pre-equilibrium between the pronucleophile, **Nu1**_H_, and the active **Nu1**^−^. Under the conditions employed for the analysis of the C–C bond-forming kinetics in reaction 1 (Sections 2 and 3), this effect is negligible because deprotonation of **Nu1**_H_ by DBU (p*K*_aH_ ∼ 13, DMSO) is extensive in the absence of the cage (*K*_1_^DBU^ = 4.2 × 10^5^ M^–1^). However, cage-catalyzed reaction 1 using the much weaker bases 2,6-di-^*t*^butylpyridine (DtBPy, p*K*_aH_ ∼ 5, DMSO) and diethylaniline (DEA, p*K*_aH_ ∼ 3, DMSO) becomes a function of both *K*_1_^C^ and *k*_2_^C^, and the bulk-phase background reaction is a function of *K*_1_^B^and *k*_2_^B^. Comparison of *K*_1_^C^ and *K*_1_^B^ under these 'weak-base' conditions then provides an estimation of the 'acidification' of the pronucleophile by virtue of host–guest complexation of **Nu1**^−^ in the tetra-cationic cage.

**Figure 5:**
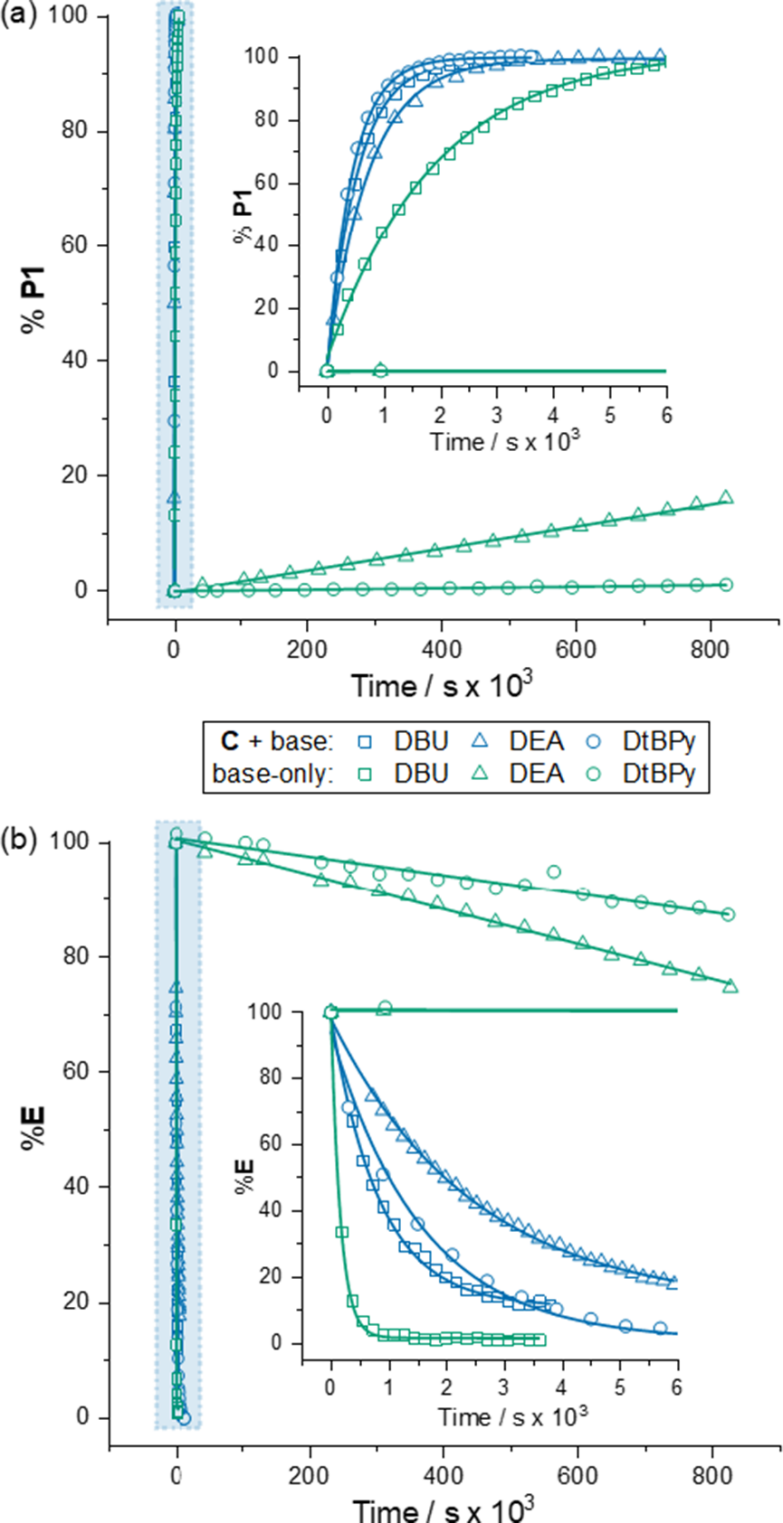
Kinetic plots for (a) reaction 1 and (b) reaction 2 with different bases.^a,b^ The fitted curves for the **C** + base and DBU alone reactions are kinetic simulations. The DEA and DtBPy alone reactions are the initial rate. ^a^DEA = diethylaniline and DtBPy = 2,6-di^t^butylpyridine. ^b^Reaction conditions. Cage-accelerated reaction 1: **C** (0.78 mM), **Nu1**_H_ (10.5 mM), **E** (3.95 mM), base (0.27 mM), CD_2_Cl_2_, RT; cage-free reaction 1: **Nu1**_H_ (1050 mM), **E** (395 mM), base (27.3 mM), CD_2_Cl_2_, RT. Cage-accelerated reaction 2: **C** (0.84 mM), **Nu1**_H_ (34.9 mM), **E** (12.4 mM), base (0.34 mM), CD_2_Cl_2_, RT. Strong base (DBU) cage-free reaction 2: base (0.34 mM), **Nu1**_H_ (34.9 mM), **E** (12.4 mM) CD_2_Cl_2_, RT. Weak base (DEA and DtBPy) cage-free reaction 2: base (33.7 mM), **Nu1**_H_ (3490 mM), **E** (1240 mM), CD_2_Cl_2_, RT.

Reaction 1 catalyzed by DtBPy or DEA alone proceeds very slowly and required monitoring at 100-fold higher concentration of all components, with the kinetics estimated from the initial rates of generation of **P1**_H_ over a period of days (Figure 5a). In contrast, the cage-catalyzed reactions, with DtBPy or DEA, and at the normal concentration of components, are complete in about 1 h (Figure 5a, inset). Analogous, although less pronounced results were obtained with reaction 2 (Figure 5b). Making the approximation that the C–C bond-forming step within the cage, *k*_2_^C^ = 2.7 × 10^1^ M^–1^ s^–1^ (Table 1), is relatively insensitive to the identity of the conjugate acid, [BH]^+^, outside of the cage, allows estimation from kinetic simulations that *K*_1_^C^ ∼ 2.4 × 10^1^ M^–1^ for DtBPy and *K*_1_^C^ ∼ 6.3 M^–1^ for DEA (Table 2). Using an analogous approximation that *k*_2_^B^ = 2.2 × 10^–2^ M^–1^ s^–1^ (Table 1) in the analysis of the initial rates of the cage-free reactions allows estimation that *K*_1_^DtBPy^∼ 2.1 × 10^–5^ M^–1^ and *K*_1_^DEA^ ∼ 3.1 × 10^–4^ M^–1^, consistent with their much lower basicity relative to DBU. The analysis shows that the H-bonded encapsulation of **Nu1**^−^ within the tetra-cationic cage C has the effect of shifting the deprotonation equilibrium of **Nu1**_H_ by a factor of 10^4^–10^6^ (Table 2).

**Table 2:** Approximate Equilibria for Base +**Nu1**_H_ and **[Nu1]^−^** [BH]^+^ Inside and Outside Cage C, Estimated from the Kinetics of Reaction 1, or by NMR Titration^a^^,^^b^^,^^c^

base	*K*_1_^C^ (M^–1^)	*K*_1_^*B*^ (M^–1^)
DBU	–d	4.2 × 10^5^^a^
DtBPy	2.4 × 10^1^^b^	2.1 × 10^–5^^c^
DEA	6.3^b^	3.1 × 10^–4^^c^

a By ^1^H NMR titration (see Supporting Information, Figures S67 and S68).

b By kinetic simulations assuming *k*_2_^C^ = 2.7 × 10^1^ M^–1^ s^–1^ (Table 1, see Supporting Information, Section S6.1.4).

c By kinetic simulations assuming *k*_2_^B^ = 2.2 × 10^–2^ M^–1^ s^–1^ (Table 1, see Supporting Information, Section S8.1.2).

d Value not established by titration, but the kinetic model requires *K*_1_^C^ > 10^4^ M^–1^.

### 2.7 Generalizing the Overall Rate Acceleration by the Cage Relative to Bulk-Phase Weak-Base Only Catalysis

Comparison of the initial rates of catalysis of reaction 1 by a weak base in the presence (*v*_0_^*C*^/M s^–1^) and absence (*v*_0_^*B*^/M s^–1^) of cage **C** allows evaluation of the acceleration over the background “bulk phase” process. The maximum acceleration (*k*_rel_^max^) exerted by the cage is approached under the conditions shown in eq 1. Steady-state initial rate approximations for reaction 1 when the base is in very low concentration and there is a substantial 'acidification' of the pronucleophile (*K*_1_^C^ ≫ 1, see the Supporting Information Section S10.2) allow estimation of the maximum accelerations attainable (eq 2). Under these conditions, the relative initial rates approach an inverse dependence on the pronucleophile concentration as the base becomes weaker, i.e., when *K*_1_^B^ ≪ 1.
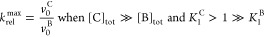
1
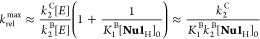
2

Consideration of the values in Tables 1 and 2 shows that very large rate accelerations are feasible. For example, when [**Nu1**_H_]_0_ ∼ 10 mM and [**C**]_tot_ ≫ [DtBPy]_tot_, eq 2 predicts that *k*_rel_^max^ ∼ 6 × 10^9^. This value can be compared to the concentration-normalized initial rates measured experimentally using DtBPy as the weak base (Table S20), where *k*_rel_^norm^ ∼ 1 × 10^9^.

## 3 Conclusions

A detailed in situ ^1^H NMR spectroscopic analysis of cage-catalyzed Michael addition reactions 1 and 2 (Scheme 1) has allowed the detection and identification of a number of cage-based species in the catalytic process (Figures 1 and 3). Numerical methods analyses of the reaction kinetics, referenced to the background “bulk-phase” base-only catalysis, including simulation of the temporal concentrations of substrates, products, and detected intermediates, led to the estimation of two key rates and equilibria: (i) C–C bond formation inside, *k*_2_^C^, and outside, *k*_2_^B^, of the cage and (ii) the pre-equilibrium between the pronucleophile and the active nucleophile, inside, *K*_1_^C^, and outside, *K*_1_^B^, of the cage. The work reported herein reinforces the importance of modeling supramolecular catalysis kinetics under more than one set of initial conditions and, importantly, conditions that give nonoptimal catalysis. Variable-temperature kinetics conducted under conditions where the rate is dominated by *k*_2_^C^[(**Nu**^–^⊂**C**][**E**] and by *k*_2_^B^[**Nu**^–^][**E**] were used to inform molecular dynamics (MD), docking, and DFT calculations of the C–C bond-forming step (Figure 2). The tensioning of these calculations against experimentally determined activation parameters (Δ*H*^‡^_exp_ and Δ*S*^‡^_exp_) allowed the identification of the origins of the reaction acceleration by the cage. Favorable electrostatic interactions between the cage and the anionic intermediates lead to a synergistic combination of raised concentrations of preorganized active species and transition-state stabilization in the C–C bond-forming step. The wealth of reactions that proceed via enolate and equivalent species suggest that there is significant potential to expand the scope of reactivity beyond Michael addition (and their asymmetric variants) using the shape and size of the cavity to match specific requirements (e.g., different selectivities, etc.).

The largest difference between the rates of cage and cage-free reactions is obtained at close to neutral conditions by using weak bases. This effect mirrors the work of Raymond and Bergman, where the optimal acid catalysis using an anionic cage occurs under basic conditions.^40^ In the work presented here, the overall effects of the cage acceleration (eq 2) are greatest at low nucleophile concentrations, with weak bases. This is not dissimilar to the way that enzymes show the largest enhancement at low substrate concentration, close to pH 7. Conducting reaction 1, for example, using DtBPy as a weak base in the presence of cage, **C**, results in a rate acceleration of 10^9^ over the “bulk phase” reference process catalyzed by the weak base alone. The magnitude of this enhancement provides another step toward creating bioinspired catalysts that move beyond mere concepts to systems that might be able to achieve enzyme-like activity derived solely from the use of weak noncovalent interactions.^15,38,49^

## Supporting Information Available

Details of reaction monitoring, kinetic data and fitting, NMR titrations, Eyring analysis, and computational modeling (PDF)
MD simulations of reactions 1 and 2 with cage and cage-free (ZIP)
DFT calculations of reactions 1 and 2 with cage and cage-free (ZIP)

## Abbreviations

DBU: 1,8-diazabicyclo (5.4.0)undec-7-ene
DEA: diethylaniline
DtBPy: 2,6-ditbutylaniline.

## References

[ref1] Piskorz T. K.; Martí-Centelles V.; Spicer R. L.; Duarte F.; Lusby P. J. Picking the lock of coordination cage catalysis. Chem.Sci. 2023, 14, 11300–11331. 10.1039/D3SC02586A. 37886081 PMC10599471

[ref2] Cramer F.; Kampe W. Inclusion Compounds. XVII.1 Catalysis of Decarboxylation by Cyclodextrins. A Model Reaction for the Mechanism of Enzymes. J. Am. Chem. Soc. 1965, 87, 1115–1120. 10.1021/ja01083a031. 14284626

[ref3] Rideout D. C.; Breslow R. Hydrophobic acceleration of Diels-Alder reactions. J. Am. Chem. Soc. 1980, 102, 7816–7817. 10.1021/ja00546a048.

[ref4] Mackay L. G.; Wylie R. S.; Sanders J. K. M. Catalytic Acyl Transfer by a Cyclic Porphyrin Trimer: Efficient Turnover without Product Inhibition. J. Am. Chem. Soc. 1994, 116, 3141–3142. 10.1021/ja00086a061.

[ref5] Tehrani F. N.; Assaf K. I.; Hein R.; Jensen C. M. E.; Nugent T. C.; Nau W. M. Supramolecular Catalysis of a Catalysis-Resistant Diels–Alder Reaction: Almost Theoretical Acceleration of Cyclopentadiene Dimerization inside Cucurbit[7]uril. ACS Catal. 2022, 12, 2261–2269. 10.1021/acscatal.1c05659.

[ref6] Sharafi M.; McKay K. T.; Ivancic M.; McCarthy D. R.; Dudkina N.; Murphy K. E.; Rajappan S. C.; Campbell J. P.; Shen Y.; Badireddy A. R.; Li J.; Schneebeli S. T. Size-Selective Catalytic Polymer Acylation with a Molecular Tetrahedron. Chem. 2020, 6, 1469–1494. 10.1016/j.chempr.2020.05.011. 32728651 PMC7388586

[ref7] Dhamija A.; Gunnam A.; Yu X.; Lee H.; Hwang I.-C.; Ho Ko Y.; Kim K. Dramatically Enhanced Reactivity of Fullerenes and Tetrazine towards the Inverse-Electron-Demand Diels–Alder Reaction inside a Porous Porphyrinic Cage. Angew. Chem., Int. Ed. 2022, 61, e202209326 10.1002/anie.202209326. 36104313

[ref8] Kang J.; Rebek J. Acceleration of a Diels–Alder reaction by a self-assembled molecular capsule. Nature 1997, 385, 50–52. 10.1038/385050a0. 8985245

[ref9] Zhang Q.; Tiefenbacher K. Terpene cyclization catalysed inside a self-assembled cavity. Nat. Chem. 2015, 7, 197–202. 10.1038/nchem.2181. 25698327

[ref10] La Manna P.; Talotta C.; Floresta G.; De Rosa M.; Soriente A.; Rescifina A.; Gaeta C.; Neri P. Mild Friedel–Crafts Reactions inside a Hexameric Resorcinarene Capsule: C–Cl Bond Activation through Hydrogen Bonding to Bridging Water Molecules. Angew. Chem., Int. Ed. 2018, 57, 5423–5428. 10.1002/anie.201801642. 29533510

[ref11] Bierschenk S. M.; Bergman R. G.; Raymond K. N.; Toste F. D. A Nanovessel-Catalyzed Three-Component Aza-Darzens Reaction. J. Am. Chem. Soc. 2020, 142, 733–737. 10.1021/jacs.9b13177. 31909615

[ref12] Murase T.; Nishijima Y.; Fujita M. Cage-catalyzed Knoevenagel condensation under neutral conditions in water. J. Am. Chem. Soc. 2012, 134, 162–164. 10.1021/ja210068f. 22145970

[ref13] Samanta D.; Mukherjee S.; Patil Y. P.; Mukherjee P. S. Self-Assembled Pd_6_ Open Cage with Triimidazole Walls and the Use of Its Confined Nanospace for Catalytic Knoevenagel- and Diels–Alder Reactions in Aqueous Medium. Chem.—Eur. J. 2012, 18, 12322–12329. 10.1002/chem.201201679. 22899180

[ref14] Bolliger J. L.; Belenguer A. M.; Nitschke J. R. Enantiopure Water-Soluble [Fe_4_L_6_] Cages: Host–Guest Chemistry and Catalytic Activity. Angew. Chem., Int. Ed. 2013, 52, 7958–7962. 10.1002/anie.201302136. 23788518

[ref15] Cullen W.; Misuraca M. C.; Hunter C. A.; Williams N. H.; Ward M. D. Highly efficient catalysis of the Kemp elimination in the cavity of a cubic coordination cage. Nat. Chem. 2016, 8, 231–236. 10.1038/nchem.2452. 26892554

[ref16] Martí-Centelles V.; Lawrence A. L.; Lusby P. J. High Activity and Efficient Turnover by a Simple, Self-Assembled “Artificial Diels–Alderase. J. Am. Chem. Soc. 2018, 140, 2862–2868. 10.1021/jacs.7b12146. 29406705

[ref17] Holloway L. R.; Bogie P. M.; Lyon Y.; Ngai C.; Miller T. F.; Julian R. R.; Hooley R. J. Tandem Reactivity of a Self-Assembled Cage Catalyst with Endohedral Acid Groups. J. Am. Chem. Soc. 2018, 140, 8078–8081. 10.1021/jacs.8b03984. 29913069

[ref18] Guo J.; Fan Y. Z.; Lu Y. L.; Zheng S. P.; Su C. Y. Visible-Light Photocatalysis of Asymmetric [2 + 2] Cycloaddition in Cage-Confined Nanospace Merging Chirality with Triplet-State Photosensitization. Angew. Chem., Int. Ed. 2020, 59, 8661–8669. 10.1002/anie.201916722. 32011801

[ref19] Zhao L.; Cai J.; Li Y.; Wei J.; Duan C. A host–guest approach to combining enzymatic and artificial catalysis for catalyzing biomimetic monooxygenation. Nat. Commun. 2020, 11, 2903 10.1038/s41467-020-16714-7. 32518257 PMC7283336

[ref20] Jiao J.; Tan C.; Li Z.; Liu Y.; Han X.; Cui Y. Design and Assembly of Chiral Coordination Cages for Asymmetric Sequential Reactions. J. Am. Chem. Soc. 2018, 140, 2251–2259. 10.1021/jacs.7b11679. 29346728

[ref21] Preston D.; Sutton J. J.; Gordon K. C.; Crowley J. D. A Nona-nuclear Heterometallic Pd_3_Pt_6_ “Donut”-Shaped Cage: Molecular Recognition and Photocatalysis. Angew. Chem., Int. Ed. 2018, 57, 8659–8663. 10.1002/anie.201804745. 29774643

[ref22] Wang Q.-Q.; Gonell S.; Leenders S. H. A. M.; Dürr M.; Ivanović-Burmazović I.; Reek J. N. H. Self-assembled nanospheres with multiple endohedral binding sites pre-organize catalysts and substrates for highly efficient reactions. Nat. Chem. 2016, 8, 225–230. 10.1038/nchem.2425. 26892553

[ref23] Fujita M.; Oguro D.; Miyazawa M.; Oka H.; Yamaguchi K.; Ogura K. Self-assembly of ten molecules into nanometre-sized organic host frameworks. Nature 1995, 378, 469–471. 10.1038/378469a0.

[ref24] Stang P. J.; Olenyuk B.; Muddiman D. C.; Smith R. D. Transition-Metal-Mediated Rational Design and Self-Assembly of Chiral, Nanoscale Supramolecular Polyhedra with Unique T Symmetry. Organometallics 1997, 16, 3094–3096. 10.1021/om9702993.

[ref25] Caulder D. L.; Powers R. E.; Parac T. N.; Raymond K. N. The Self-Assembly of a Predesigned Tetrahedral M4L6 Supramolecular Cluster. Angew. Chem., Int. Ed. 1998, 37, 1840–1843. 10.1002/(SICI)1521-3773(19980803)37:13/14<1840::AID-ANIE1840>3.0.CO;2-D.

[ref26] Mal P.; Schultz D.; Beyeh K.; Rissanen K.; Nitschke J. R. An Unlockable–Relockable Iron Cage by Subcomponent Self-Assembly. Angew. Chem., Int. Ed. 2008, 47, 8297–8301. 10.1002/anie.200803066. 18729112

[ref27] Bloch W. M.; Holstein J. J.; Hiller W.; Clever G. H. Morphological Control of Heteroleptic *cis*- and *trans*-Pd(_2_)L(_2_)L’(_2_) Cages. Angew. Chem., Int. Ed. 2017, 56, 8285–8289. 10.1002/anie.201702573. PMC5499718 28544072

[ref28] Pearcy A. C.; Lisboa L. S.; Preston D.; Page N. B.; Lawrence T.; Wright L. J.; Hartinger C. G.; Crowley J. D. Exploiting reduced-symmetry ligands with pyridyl and imidazole donors to construct a second-generation stimuli-responsive heterobimetallic [PdPtL_4_]^4+^ cage. Chem. Sci. 2023, 14, 8615–8623. 10.1039/D3SC01354E. 37592996 PMC10430685

[ref29] Yazaki K.; Akita M.; Prusty S.; Chand D. K.; Kikuchi T.; Sato H.; Yoshizawa M. Polyaromatic molecular peanuts. Nat. Commun. 2017, 8, 15914 10.1038/ncomms15914. 28656977 PMC5493752

[ref30] Ube H.; Endo K.; Sato H.; Shionoya M. Synthesis of Hetero-multinuclear Metal Complexes by Site-Selective Redox Switching and Transmetalation on a Homo-multinuclear Complex. J. Am. Chem. Soc. 2019, 141, 10384–10389. 10.1021/jacs.9b04123. 31189315

[ref31] Abe T.; Sanada N.; Takeuchi K.; Okazawa A.; Hiraoka S. Assembly of Six Types of Heteroleptic Pd_2_L_4_ Cages under Kinetic Control. J. Am. Chem. Soc. 2023, 145, 28061–28074. 10.1021/jacs.3c09359. 38096127 PMC10755705

[ref32] Molinska P.; Tarzia A.; Male L.; Jelfs K. E.; Lewis J. E. M. Diastereoselective Self-Assembly of Low-Symmetry Pd_n_L_2n_ Nanocages through Coordination-Sphere Engineering. Angew. Chem., Int. Ed. 2023, 62, e202315451 10.1002/anie.202315451. PMC10952360 37888946

[ref33] Dasary H.; Jagan R.; Chand D. K. Ligand Isomerism in Coordination Cages. Inorg. Chem. 2018, 57, 12222–12231. 10.1021/acs.inorgchem.8b01884. 30230331

[ref34] Cook T. R.; Stang P. J. Recent Developments in the Preparation and Chemistry of Metallacycles and Metallacages via Coordination. Chem. Rev. 2015, 115, 7001–7045. 10.1021/cr5005666. 25813093

[ref35] Yoshizawa M.; Tamura M.; Fujita M. Diels-Alder in Aqueous Molecular Hosts: Unusual Regioselectivity and Efficient Catalysis. Science 2006, 312, 251–254. 10.1126/science.1124985. 16614218

[ref36] Li L.; Yang L.; Li X.; Wang J.; Liu X.; He C. Supramolecular Catalysis of Acyl Transfer within Zinc Porphyrin-Based Metal–Organic Cages. Inorg. Chem. 2021, 60, 8802–8810. 10.1021/acs.inorgchem.1c00745. 34085514

[ref37] Fiedler D.; Bergman R. G.; Raymond K. N. Supramolecular Catalysis of a Unimolecular Transformation: Aza-Cope Rearrangement within a Self-Assembled Host. Angew. Chem., Int. Ed. 2004, 43, 6748–6751. 10.1002/anie.200461776. 15558640

[ref38] Hastings C. J.; Pluth M. D.; Bergman R. G.; Raymond K. N. Enzymelike Catalysis of the Nazarov Cyclization by Supramolecular Encapsulation. J. Am. Chem. Soc. 2010, 132, 6938–6940. 10.1021/ja102633e. 20443566

[ref39] Kaphan D. M.; Toste F. D.; Bergman R. G.; Raymond K. N. Enabling New Modes of Reactivity via Constrictive Binding in a Supramolecular-Assembly-Catalyzed A -Prins Cyclization. J. Am. Chem. Soc. 2015, 137, 9202–9205. 10.1021/jacs.5b01261. 26176416

[ref40] Pluth M. D.; Bergman R. G.; Raymond K. N. Acid catalysis in basic solution: a supramolecular host promotes orthoformate hydrolysis. Science 2007, 316, 85–88. 10.1126/science.1138748. 17412953

[ref41] Wang J.; Young T. A.; Duarte F.; Lusby P. J. Synergistic Noncovalent Catalysis Facilitates Base-Free Michael Addition. J. Am. Chem. Soc. 2020, 142, 17743–17750. 10.1021/jacs.0c08639. 32927950

[ref42] Ludden M. D.; Taylor C. G. P.; Tipping M. B.; Train J. S.; Williams N. H.; Dorrat J. C.; Tuck K. L.; Ward M. D. Interaction of anions with the surface of a coordination cage in aqueous solution probed by their effect on a cage-catalysed Kemp elimination. Chem. Sci. 2021, 12, 14781–14791. 10.1039/D1SC04887B. 34820094 PMC8597839

[ref43] Vachal P.; Jacobsen E. N. Structure-Based Analysis and Optimization of a Highly Enantioselective Catalyst for the Strecker Reaction. J. Am. Chem. Soc. 2002, 124, 10012–10014. 10.1021/ja027246j. 12188665

[ref44] Zuend S. J.; Jacobsen E. N. Mechanism of Amido-Thiourea Catalyzed Enantioselective Imine Hydrocyanation: Transition State Stabilization via Multiple Non-Covalent Interactions. J. Am. Chem. Soc. 2009, 131, 15358–15374. 10.1021/ja9058958. 19778044 PMC2783581

[ref45] Dale H. J. A.; Hodges G. R.; Lloyd-Jones G. C. Taming Ambident Triazole Anions: Regioselective Ion Pairing Catalyzes Direct N-Alkylation with Atypical Regioselectivity. J. Am. Chem. Soc. 2019, 141, 7181–7193. 10.1021/jacs.9b02786. 30943722

[ref46] Wang J.; Horwitz M. A.; Dürr A. B.; Ibba F.; Pupo G.; Gao Y.; Ricci P.; Christensen K. E.; Pathak T. P.; Claridge T. D. W.; Lloyd-Jones G. C.; Paton R. S.; Gouverneur V. Asymmetric Azidation under Hydrogen Bonding Phase-Transfer Catalysis: A Combined Experimental and Computational Study. J. Am. Chem. Soc. 2022, 144, 4572–4584. 10.1021/jacs.1c13434. 35230845 PMC8931729

[ref47] Zhao Y.; Beuchat C.; Domoto Y.; Gajewy J.; Wilson A.; Mareda J.; Sakai N.; Matile S. Anion−π Catalysis. J. Am. Chem. Soc. 2014, 136, 2101–2111. 10.1021/ja412290r. 24456523

[ref48] Paraja M.; Hao X.; Matile S. Polyether Natural Product Inspired Cascade Cyclizations: Autocatalysis on π-Acidic Aromatic Surfaces. Angew. Chem., Int. Ed. 2020, 59, 15093–15097. 10.1002/anie.202000681. 32181559

[ref49] Kaphan D. M.; Levin M. D.; Bergman R. G.; Raymond K. N.; Toste F. D. A supramolecular microenvironment strategy for transition metal catalysis. Science 2015, 350, 1235–1238. 10.1126/science.aad3087. 26785485

[ref50] Pluth M. D.; Bergman R. G.; Raymond K. N. The Acid Hydrolysis Mechanism of Acetals Catalyzed by a Supramolecular Assembly in Basic Solution. J. Org. Chem. 2009, 74, 58–63. 10.1021/jo802131v. 19113901

[ref51] Hong C. M.; Morimoto M.; Kapustin E. A.; Alzakhem N.; Bergman R. G.; Raymond K. N.; Toste F. D. Deconvoluting the Role of Charge in a Supramolecular Catalyst. J. Am. Chem. Soc. 2018, 140, 6591–6595. 10.1021/jacs.8b01701. 29767972

[ref52] Frushicheva M. P.; Mukherjee S.; Warshel A. Electrostatic Origin of the Catalytic Effect of a Supramolecular Host Catalyst. J. Phys. Chem. B 2012, 116, 13353–13360. 10.1021/jp3084327. 23088306 PMC3536831

[ref53] Ootani Y.; Akinaga Y.; Nakajima T. Theoretical investigation of enantioselectivity of cage-like supramolecular assembly: The insights into the shape complementarity and host–guest interaction. J. Comput. Chem. 2015, 36, 459–466. 10.1002/jcc.23821. 25565267

[ref54] Vaissier Welborn V.; Head-Gordon T. Electrostatics Generated by a Supramolecular Capsule Stabilizes the Transition State for Carbon–Carbon Reductive Elimination from Gold(III) Complex. J. Phys. Chem. Lett. 2018, 9, 3814–3818. 10.1021/acs.jpclett.8b01710. 29939756

[ref55] Norjmaa G.; Maréchal J.-D.; Ujaque G. Microsolvation and Encapsulation Effects on Supramolecular Catalysis: C–C Reductive Elimination inside [Ga_4_L_6_]^12–^ Metallocage. J. Am. Chem. Soc. 2019, 141, 13114–13123. 10.1021/jacs.9b04909. 31390202

[ref56] Young T. A.; Martí-Centelles V.; Wang J.; Lusby P. J.; Duarte F. Rationalizing the Activity of an “Artificial Diels-Alderase”: Establishing Efficient and Accurate Protocols for Calculating Supramolecular Catalysis. J. Am. Chem. Soc. 2020, 142, 1300–1310. 10.1021/jacs.9b10302. 31852191

[ref57] Nguyen Q. N. N.; Xia K. T.; Zhang Y.; Chen N.; Morimoto M.; Pei X.; Ha Y.; Guo J.; Yang W.; Wang L.-P.; Bergman R. G.; Raymond K. N.; Toste F. D.; Tantillo D. J. Source of Rate Acceleration for Carbocation Cyclization in Biomimetic Supramolecular Cages. J. Am. Chem. Soc. 2022, 144, 11413–11424. 10.1021/jacs.2c04179. 35699585

[ref58] Delle Piane M.; Pesce L.; Cioni M.; Pavan G. M. Reconstructing reactivity in dynamic host–guest systems at atomistic resolution: amide hydrolysis under confinement in the cavity of a coordination cage. Chem. Sci. 2022, 13, 11232–11245. 10.1039/D2SC02000A. 36320487 PMC9517058

[ref59] Mayr H.; Patz M. Scales of Nucleophilicity and Electrophilicity: A System for Ordering Polar Organic and Organometallic Reactions. Angew. Chem., Int. Ed. 1994, 33, 938–957. 10.1002/anie.199409381.

[ref60] Ben-Tal Y.; Boaler P. J.; Dale H. J. A; Dooley R. E.; Fohn N. A.; Gao Y.; García-Domínguez A.; Grant K. M.; Hall A. M. R.; Hayes H. D. L.; Kucharski M. M.; Wei R.; Lloyd-Jones G. C. Mechanistic Analysis by NMR Spectroscopy: a Users Guide. Prog. Nucl. Magn. Reson. Spectrosc. 2022, 129, 28–106. 10.1016/j.pnmrs.2022.01.001. 35292133

[ref61] August D. P.; Nichol G. S.; Lusby P. J. Maximizing Coordination Capsule–Guest Polar Interactions in Apolar Solvents Reveals Significant Binding. Angew. Chem., Int. Ed. 2016, 55, 15022–15026. 10.1002/anie.201608229. 27809382

[ref62] Bickelhaupt F. M.; Houk K. N. Analyzing Reaction Rates with the Distortion/Interaction-Activation Strain Model. Angew. Chem., Int. Ed. 2017, 56, 10070–10086. 10.1002/anie.201701486. PMC5601271 28447369

[ref63] O’Connor H. M.; Tipping W. J.; Vallejo J.; Nichol G. S.; Faulds K.; Graham D.; Brechin E. K.; Lusby P. J. Utilizing Raman Spectroscopy as a Tool for Solid- and Solution-Phase Analysis of Metalloorganic Cage Host–Guest Complexes. Inorg. Chem. 2023, 62, 1827–1832. 10.1021/acs.inorgchem.2c00873. 35512336 PMC9906719

