# Supplementary material for: Origins of High-Activity Cage-Catalyzed Michael Addition
Source: J Am Chem Soc. 2024 Jul 8;146(28):19317–26. doi: 10.1021/jacs.4c05160 (PMC11258793; doi:10.1021/jacs.4c05160)
Supplement: Supplementary file 1 — ja4c05160_si_001.pdf [file ja4c05160_si_001.pdf]

Supporting Information

The Origins of High-Activity Cage-Catalyzed Michael Addition

Patrick J. Boaler,[a] Tomasz K. Piskorz,[b] Laura E. Bickerton,[a] Jianzhu Wang,[a]

Fernanda Duarte,[b]* Guy. C. Lloyd-Jones[a]* and Paul J. Lusby[a]*

[a]EaStCHEM School of Chemistry, University of Edinburgh, Joseph Black Building, David Brewster Road, Edinburgh, Scotland, EH9 3FJ, U.K.

[b]Chemistry Research Laboratory, University of Oxford, Oxford OX1 3TA, U.K.

Paul.Lusby@ed.ac.uk

Guy.Lloyd-Jones@ed.ac.uk

fernanda.duartegonzalez@chem.ox.ac.uk

S1.1 Table of contents

S1.1 Table of contents 2
S2. Abbreviations 5
S3. Materials and Methods 6
S3.1 Synthesis of cage catalyst 6
S3.2 Purification of substrates 6
S4. *In situ* reaction monitoring of Cage-catalysed Michael additions *via* ^{1}H NMR spectroscopy 7
S4.1 General Considerations 7
S4.1.1 Representative procedure for the reaction of $Nu2_H$ and E catalysed by C and DBU 7
S4.1.2 Spectral processing 7
S4.2 Spectra from ^{1}H NMR reaction monitoring 8
S4.2.1 Reaction 1 8
S4.2.2 Reaction 2 15
S4.3 Temporal concentrations 23
S4.3.1 Reaction 1 23
S4.3.2 Reaction 2 25
S5. Intermediate characterisation 27
S5.1 $Nu1^{-}\subset C$ and $P1^{-}\subset C$ 27
S5.2 $Nu2^{-}\subset C$ and $P2^{-}\subset C$ 28
S6. Kinetic modelling of cage-catalysed Michael addition reactions 29
S6.1 Reaction 1 Models 29
S6.1.1 Model 1 30
S6.1.2 Model 2 34
S6.1.3 Model 3 38
S6.1.4 Model 4 42
S6.2 Reaction 2 Model 46
S6.2.1 E excess 48
S6.2.2 $Nu2_H$ Excess 50
S7. NMR titrations 52
S7.1 Titration of $Nu1_H$ with DBU in the absence of C 53
S7.2 Titrations of $Nu2_H$ with DBU in the absence of C 54
S7.3 Titrations of $Nu1_H$ with DBU in the presence of C 55

S7.3.1 Competitive titration of DBU in the presence of Anthraquinone (AQ) and excess $Nu1_H$ ... 55
S7.3.2 Direct titration of DBU into C in the presence of excess $Nu1_H$ ... 57
S8. DBU catalysed Michael addition in the absence of C ... 58
S8.1 Reaction 1 ... 59
S8.1.1 Spectra ... 59
S8.1.2 Reaction 1 Model ... 61
S8.2 Reaction 2 ... 62
S8.2.1 Spectra ... 62
S8.2.2 Discussion ... 64
S9. Eyring analysis ... 69
S9.1 Cage Catalysed Reaction ... 70
S9.2 DBU-only reaction ... 72
S9.3 Error Analysis ... 73
S9.3.1 Cage catalysed reaction 1 ... 74
S9.3.2 DBU-only reaction 1 ... 74
S9.3.3 Summary of Eyring analysis ... 74
S10. Effect of alternative bases and a description of rate enhancement by cage ... 76
S10.1 Estimation of $K1B$ and $K1C$ ... 76
S10.2 Extent of acceleration $krelmax$ for cage-catalysed Michael additions ... 84
S10.3 Alternative $krelmax$ derivation by approximation ... 86
S11. Computational methods ... 87
S11.1 Quantum calculations ... 87
S11.2 Molecular dynamics ... 87
S12. Additional computational results ... 89
S12.1 Reaction 1 ... 89
S12.1.1 Cage-free reaction ... 89
S12.1.2 Benchmark studies ... 90
S12.1.3 Catalysed reaction ... 91
S12.2 Reaction 2 ... 93
S12.2.1 Cage-free reaction ... 93
S12.2.2 Catalysed reaction ... 96
S13. References ... 99

S2. Abbreviations

DBU – 1,8-Diazabicyclo[5.4.0]undec-7-ene

DEA – Diethylaniline

DtBPy – Di-(tert-butyl)-pyridine

TMS – Trimethylsilyl

m – multiplet

q – quartet

t – triplet

d – doublet

s – singlet

t_0 – Initial time

ppm – parts per million

J – scalar coupling constant

M – mol dm^{-3} (moles per cubic decimetre)

mM – mmol dm^{-3} (millimoles per cubic decimetre)

S3. Materials and Methods

Unless otherwise stated, all reagents and solvents were purchased from Alfa Aesar, VWR, Fluorochem or Sigma Aldrich and used without further purification. Column chromatography was carried out using Geduran Si60 (40-63 µm) as the stationary phase and TLC was performed on precoated Kieselgel 60 plates (0.20 mm thick, 60F254. Merck, Germany) and observed under UV light at 254 nm. All reactions were carried out under air and at room temperature, unless otherwise stated.

All ^{1}H and ^{13}C NMR spectra were recorded on either a 500 MHz Bruker AV III equipped with a DCH cryo-probe (Ava500), a 400MHZ Bruker AV III equipped with BBFO+ probe (Ava400), a 500 MHz Bruker AV IIIHD equipped with a Prodigy cryo-probe (Pro500) or a 600 MHz Bruker AV IIIHD equipped with a TCI cryo-probe (Ava600) at a constant temperature of 300 K. Chemical shifts are reported in parts per million (ppm). Coupling constants (J) are reported in Hertz (Hz). All spectral processing was performed with MestReNova, Version 14.0.0.

S3.1 Synthesis of cage catalyst

Catalyst C was synthesised according to a previously reported literature procedure.[1]

S3.2 Purification of substrates

Methyl nitroacetate (**Nu1**$_H$) and malononitrile (**Nu2**$_H$) were purified by silica plug using CH_2Cl_2 as an eluent. Methyl vinyl ketone (**E**) was purified by distillation under reduced pressure at room temperature. All purified substrates were stored neat at -20°C, and CD_2Cl_2 stock solutions prepared as required.

S4. *In situ* reaction monitoring of Cage-catalysed Michael additions *via* ^{1}H NMR spectroscopy

S4.1 General Considerations

All reaction monitoring was conducted using a total sample volume of 600 µL, using $Si(TMS)_4$ as an internal standard. All reagents were added as stock solutions prepared in CD_2Cl_2 with the exception of **C**, which was added to the NMR tube first, directly as a solid.

S4.1.1 Representative procedure for the reaction of $Nu2_H$ and E catalysed by C and DBU

An NMR tube was charged 2.9 mg **C** (6.1×10^{-4} mmol, 1 mM in 600 µL) and 506 µL CD_2Cl_2, followed by $Si(TMS)_4$ (30 µL of a 19.9 mM stock solution), malononitrile (30 µL of a 606 mM stock solution) and methyl vinyl ketone (30 µL of a 240 mM stock solution). After mixing, the sample was inserted into the spectrometer and the t_0 spectrum was recorded. The spectrometer parameters were then used to set up a pseudo2D ^{1}H pulse sequence for reaction monitoring. The reaction was initiated by addition of DBU (4 µL of a 267 mM stock solution) and shaken to ensure thorough mixing sample. The sample tube was then inserted immediately into the spectrometer and the shimming re-optimised before acquisition. The reactions were then monitored using the pre-calibrated pseudo2D pulse sequence.

S4.1.2 Spectral processing

All NMR spectra were processed in MNova version 14 according to a processing template comprising automatic phase correction, baseline correction using the Whittaker smoother method, solvent chemical shift referencing and block-wise non-local means denoising.

Concentrations were extracted from the integral of appropriate resonances, normalised by the integral of the internal standard. In cases where more than one resonance was observable for a given species, the integral of only one resonance was used, chosen by prioritising absolute integral and separation from resonances corresponding to other species.

S4.2 Spectra from ^{1}H NMR reaction monitoring

S4.2.1 Reaction 1

The Michael addition reaction between methyl nitroacetate (**Nu1**$_H$) and methyl vinyl ketone (**E**) Figure S1 in the presence of catalytic DBU and **C** was studied at two different initial conditions, outlined in table S1.

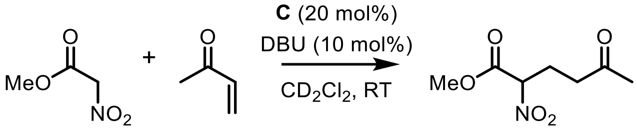

Nu1$_H$ **E** **P1**$_H$

*Figure S1. DBU-catalysed Michael addition reaction between **Nu1**$_H$ and **E** (reaction 1) in the presence of catalytic **C**.*

Table S1. Initial concentrations and name for the different conditions under which reaction 1 was studied.

Conditions	$[E]_0$ / mM	$[Nu1_H]_0$ / mM	$[C]_0$ / mM	$[DBU]_0$ / mM
"Nu2$_H$ Excess"	3.95	10.5	0.784	0.273
"Equimolar"	4.21	5.13	0.501	0.303

S4.2.1.1 Nu1$_H$ Excess

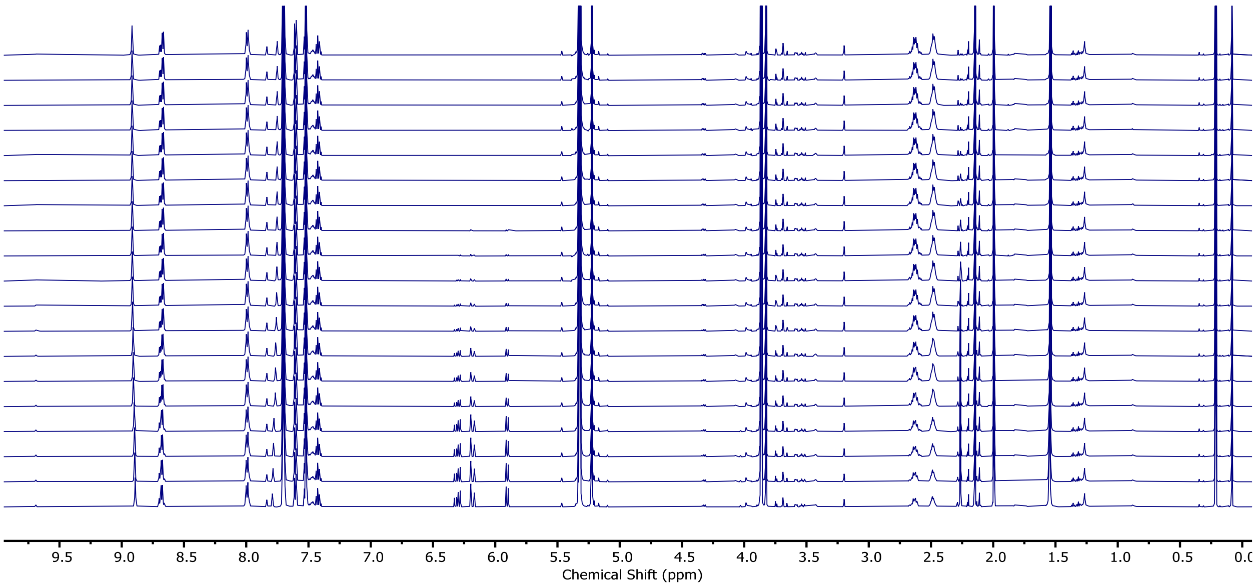

Figure S2. Temporal ^{1}H NMR Spectra (600 MHz) of the DBU catalysed reaction of ***Nu1$_H$*** *and* ***E****, in the presence of* ***C*** *under* ***Nu1$_H$*** *Excess conditions over the course of 1 hour. The time increments correspond to those shown in Figures S29 and S30.*

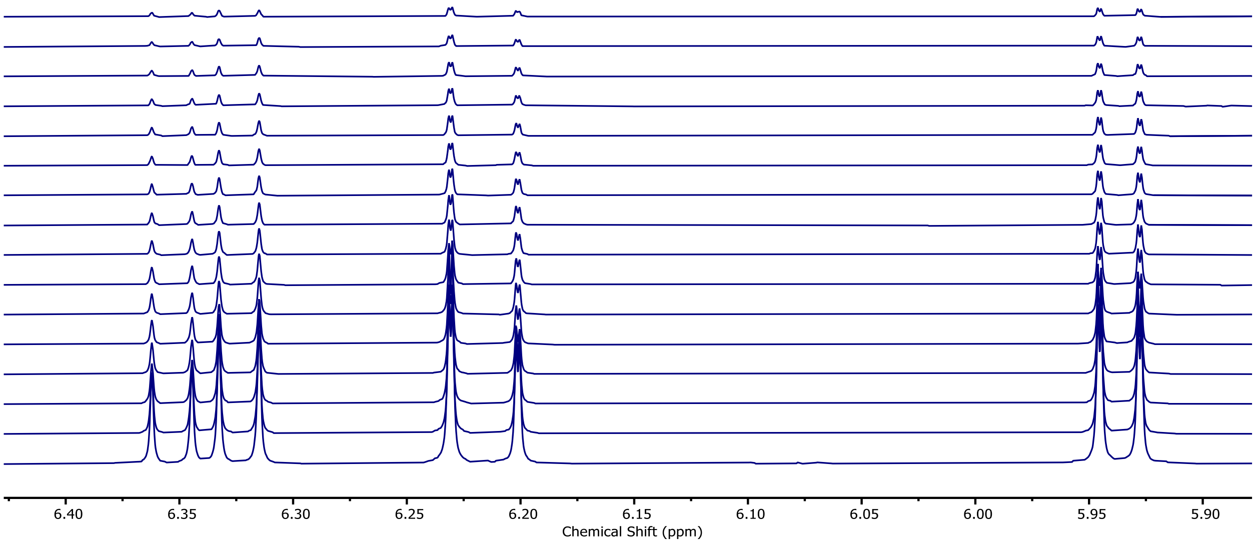

Figure S3. Partial temporal ^{1}H NMR Spectra (600 MHz) of the DBU catalysed reaction of ***Nu1$_H$*** *and* ***E****, in the presence of* ***C*** *showing the consumption of limiting substrate* ***E*** *under* ***Nu1$_H$*** *Excess conditions over the course of 1 hour. The time increments correspond to those shown in Figures S29 and S30.*

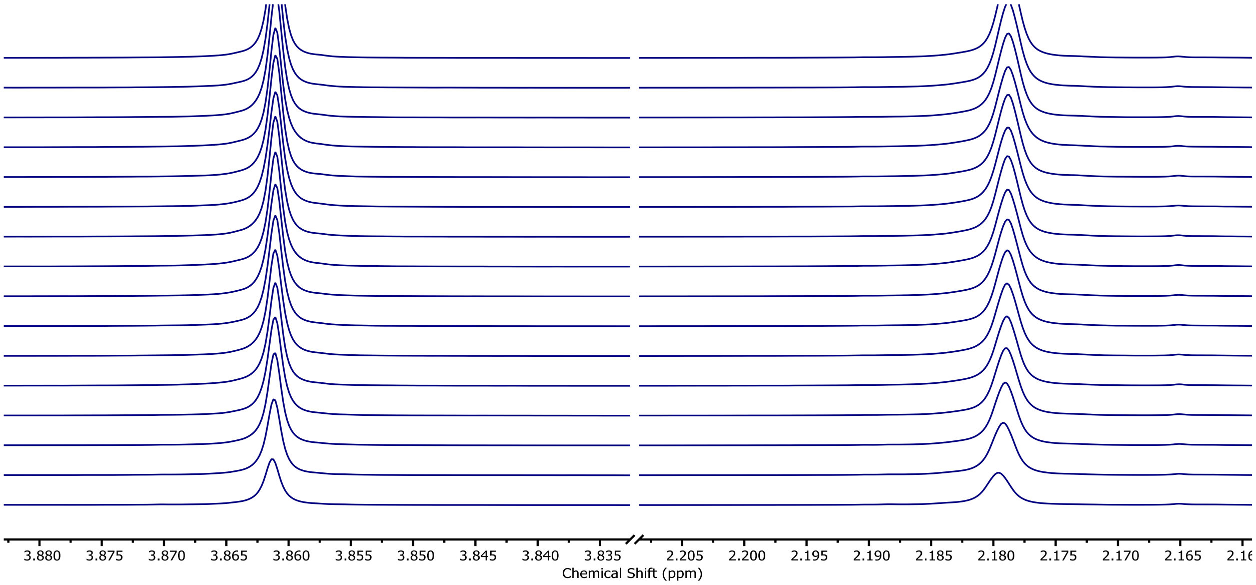

Figure S4. Partial temporal ^{1}H NMR Spectra (600 MHz) of the DBU catalysed reaction of $\mathbf{Nu1}_H$ *and* **E***, in the presence of* **C***, showing the formation of product* $\mathbf{P1}_H$ *under* $\mathbf{Nu1}_H$ *Excess conditions over the course of 1 hour. The time increments correspond to those shown in Figures S29 and S30.*

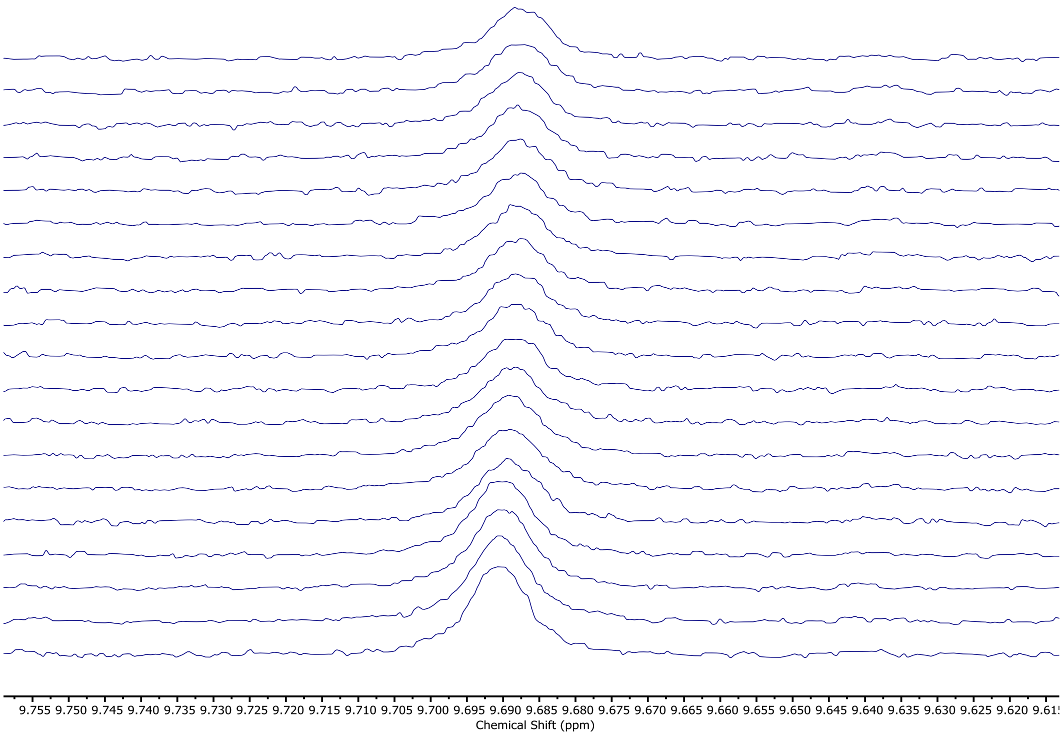

Figure S5. Partial temporal ^{1}H NMR Spectra (600 MHz) of the DBU catalysed reaction of $\mathbf{Nu1}_H$ *and* **E***, in the presence of* **C***, showing the consumption of* **C** *derived intermediate* $\mathbf{Nu1}^{-}\subset\mathbf{C}$ *under* $\mathbf{Nu1}_H$ *Excess conditions over the course of 1 hour. The time increments correspond to those shown in Figure S30.*

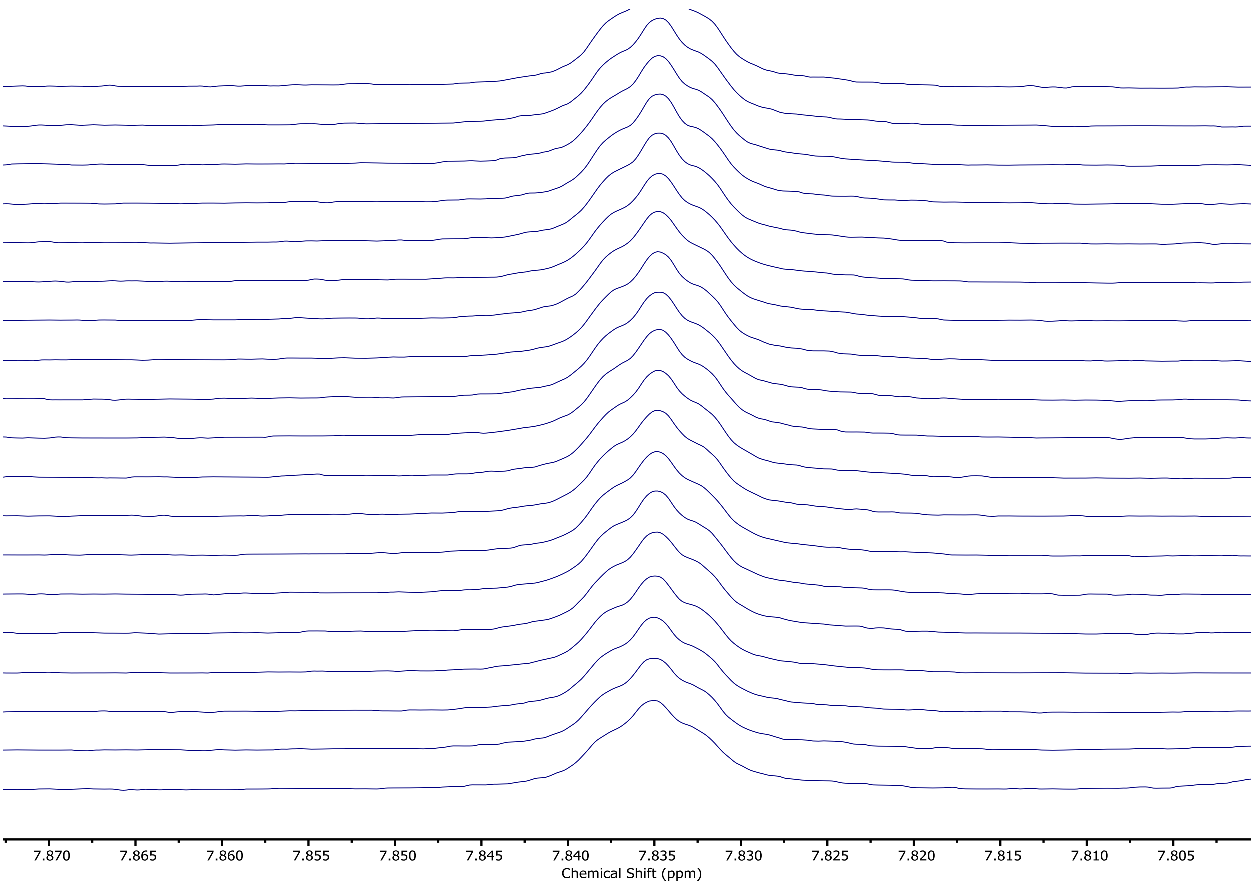

Figure S6. Partial temporal ^{1}H NMR Spectra (600 MHz) of the DBU catalysed reaction of ***Nu1**$_H$* *and* ***E**, in the presence of* ***C**,* *showing the formation of* ***C*** *derived intermediate* ***P1$^-$⊂C*** *under* ***Nu1**$_H$* *Excess conditions over the course of 1 hour. The time increments correspond to those shown in Figure S30.*

S4.2.1.2 Equimolar

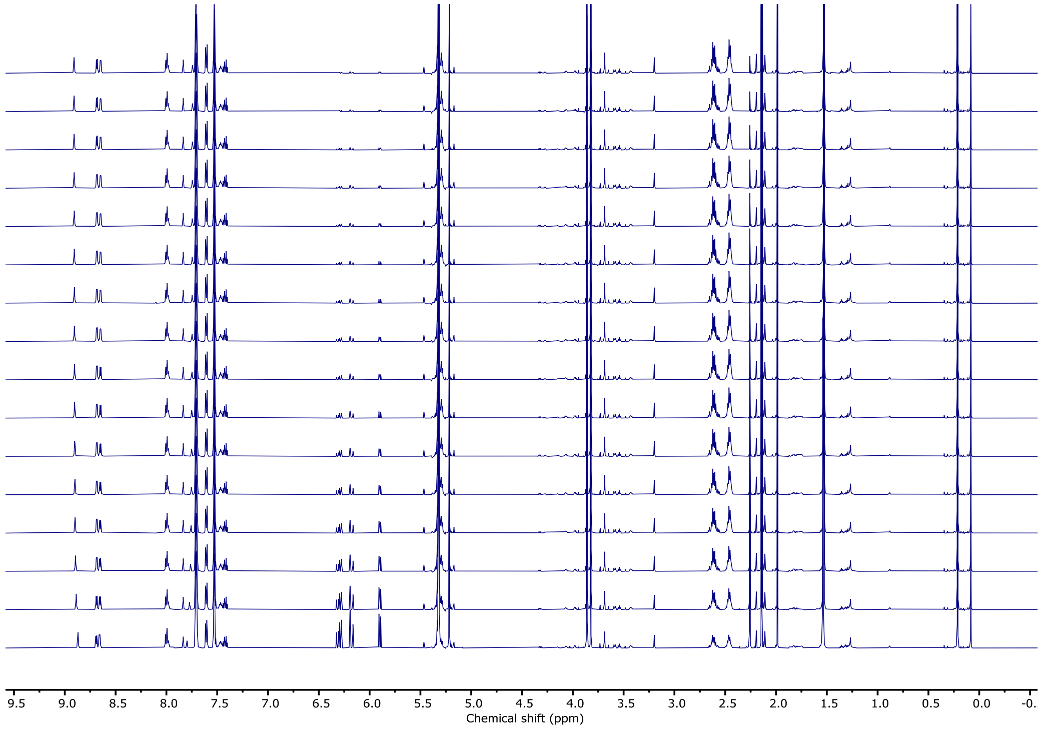

*Figure S7. Temporal ^{1}H NMR Spectra (600 MHz) of the DBU catalysed reaction of **Nu1**$_H$ and **E** under equimolar conditions, in the presence of **C** under equimolar conditions over the course of 2.5 hours. The time increments correspond to those shown in Figures S31 and S32.*

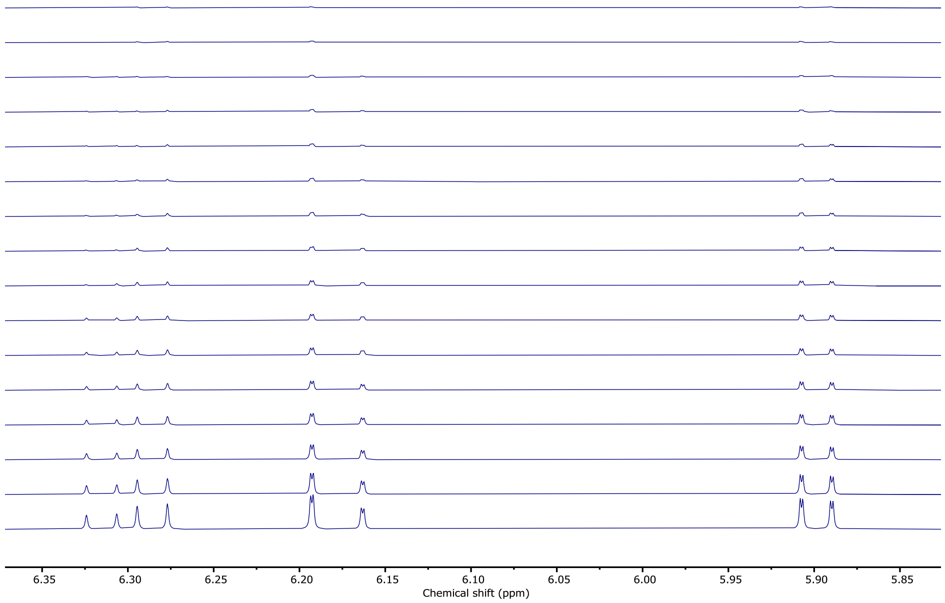

*Figure S8. Partial temporal ^{1}H NMR Spectra (600 MHz) of the DBU catalysed reaction of **Nu1**$_H$ and **E** under equimolar conditions, in the presence of **C**, showing the consumption of **E** under equimolar conditions over the course of 2.5 hours. The time increments correspond to those shown in Figures S31 and S32.*

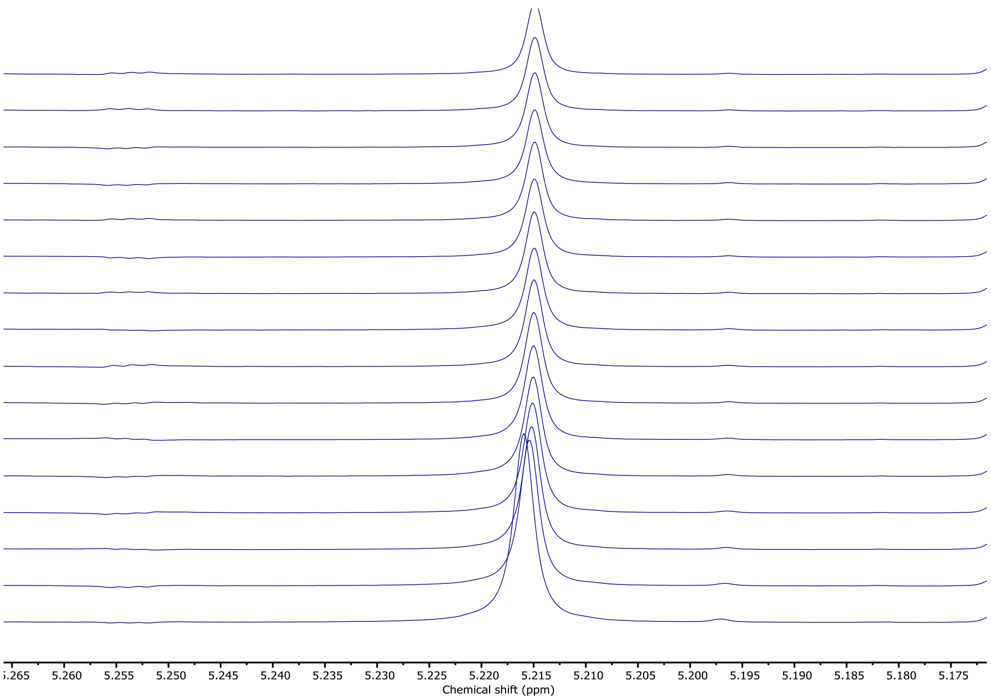

Figure S9. Partial temporal ¹H NMR Spectra (600 MHz) of the DBU catalysed reaction of ***$Nu1_H$*** *and* ***E*** *under equimolar conditions, in the presence of* ***C****, showing the consumption of* ***$Nu1_H$*** *under equimolar conditions over the course of 2.5 hours. The time increments correspond to those shown in Figures S31 and S32.*

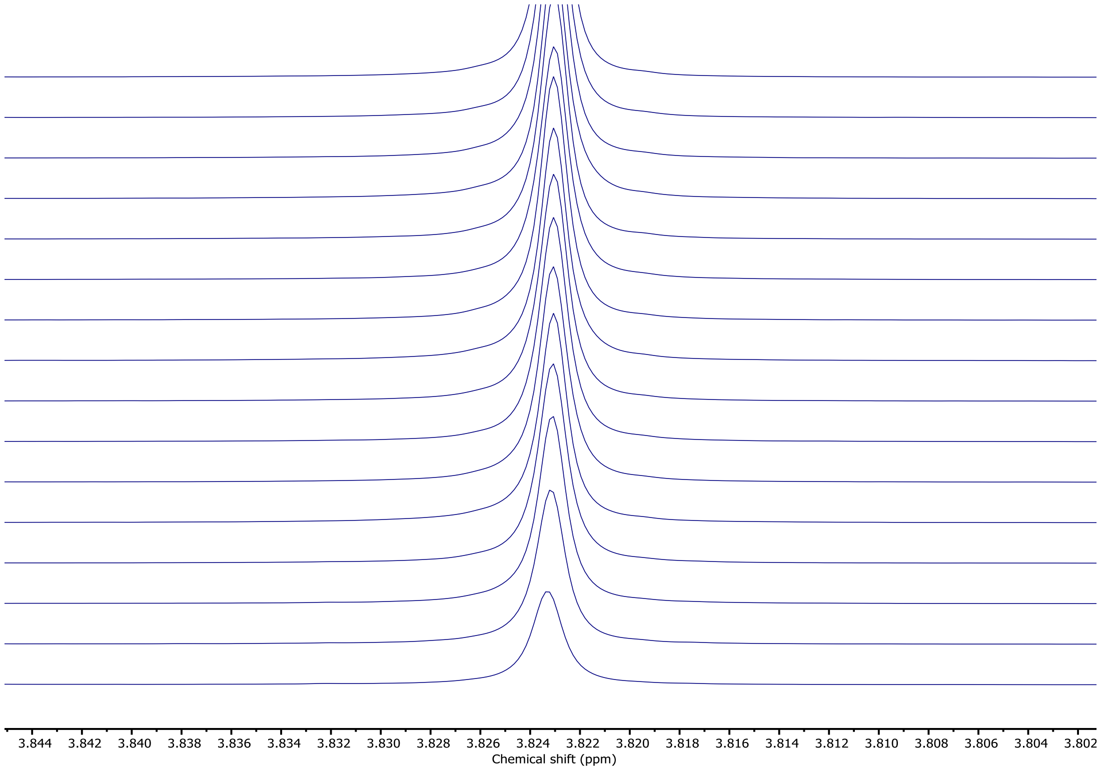

Figure S10. Partial temporal ¹H NMR Spectra (600 MHz) of the DBU catalysed reaction of ***$Nu1_H$*** *and* ***E*** *under equimolar conditions, in the presence of* ***C****, showing the production of* ***$P1_H$*** *under equimolar conditions over the course of 2.5 hours. The time increments correspond to those shown in Figures S31 and S32.*

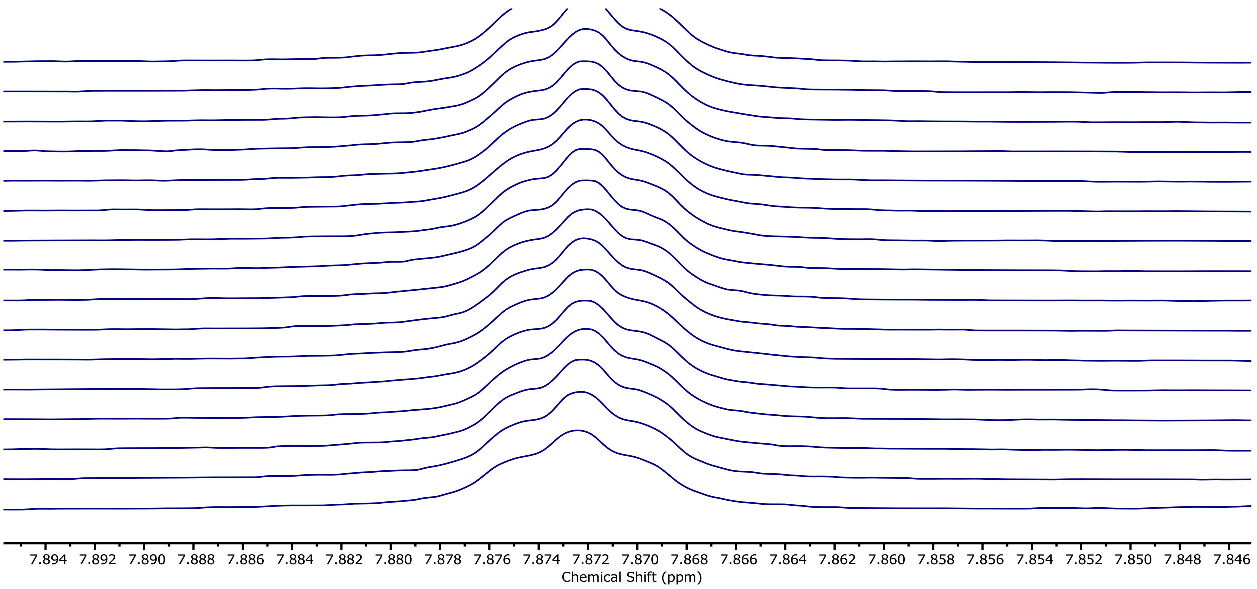

*Figure S11. Partial temporal ^{1}H NMR Spectra (600 MHz) of the DBU catalysed reaction of **Nu1**$_H$ and **E**, in the presence of **C**, showing the formation of **P1**$^-$⊂**C** under equimolar conditions over the course of 2.5 hours. The time increments correspond to those shown in Figure S32.*

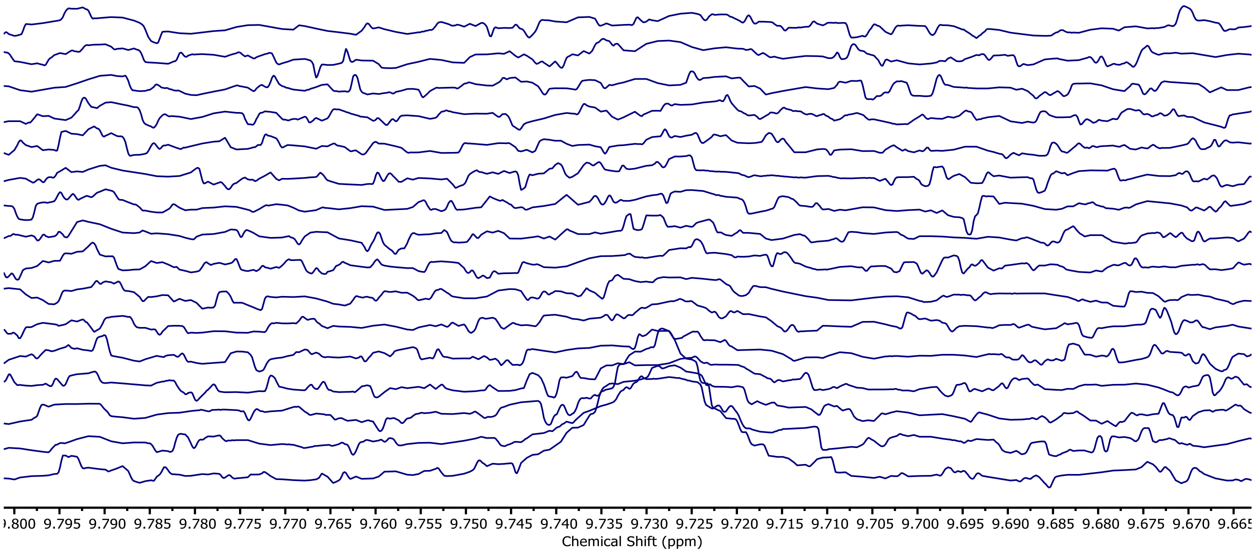

*Figure S12. Partial temporal ^{1}H NMR Spectra (600 MHz) of the DBU catalysed reaction of **Nu1**$_H$ and **E**, in the presence of **C**, showing the formation of **Nu1**$^-$⊂**C** under equimolar conditions over the course of 2.5 hours. The time increments correspond to those shown in Figure S32.*

S4.2.2 Reaction 2

The Michael addition reaction between malononitrile (**Nu2$_H$**) and methyl vinyl ketone (**E**) (figure S13) in the presence of catalytic DBU and **C** was studied at two different initial conditions, outlined in table S2.

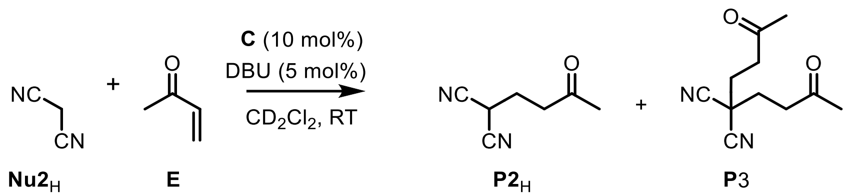

*Figure S13. DBU-catalysed Michael addition reaction between **Nu1$_H$** and **E** (reaction 1) in the presence of catalytic **C**.*

Table S2. Initial concentrations for the different conditions under which reaction 2 was studied.

Entry	$[E]_0$ / mM	$[Nu2_H]_0$ / mM	$[C]_0$ / mM	$[DBU]_0$ / mM
1	33.5	11.6	0.689	0.360
2	12.4	34.9	0.840	0.337

S4.2.2.1 E Excess

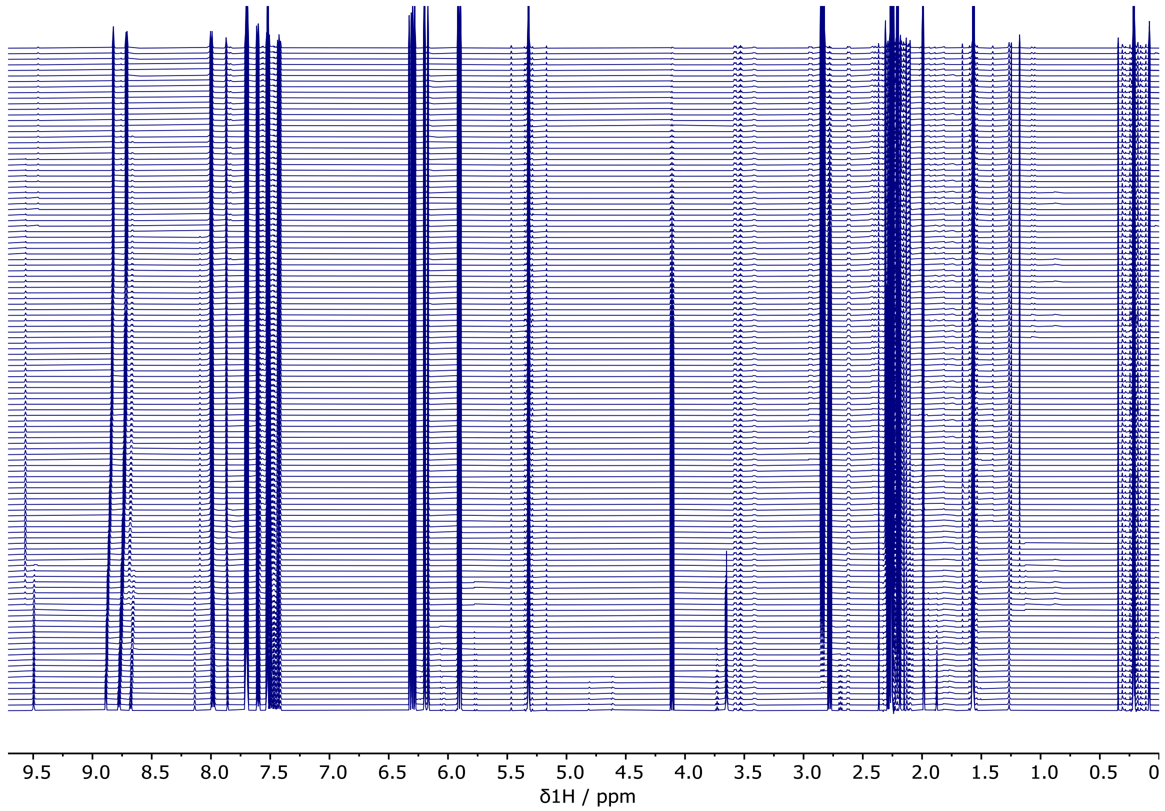

*Figure S14. Temporal ^{1}H NMR Spectra (600 MHz) of the DBU (0.360 mM) catalysed reaction of **Nu2$_H$** (11.6 mM) and an excess of **E** (33.1 mM), in the presence of **C** (0.689 mM) over the course of 1 hour.*

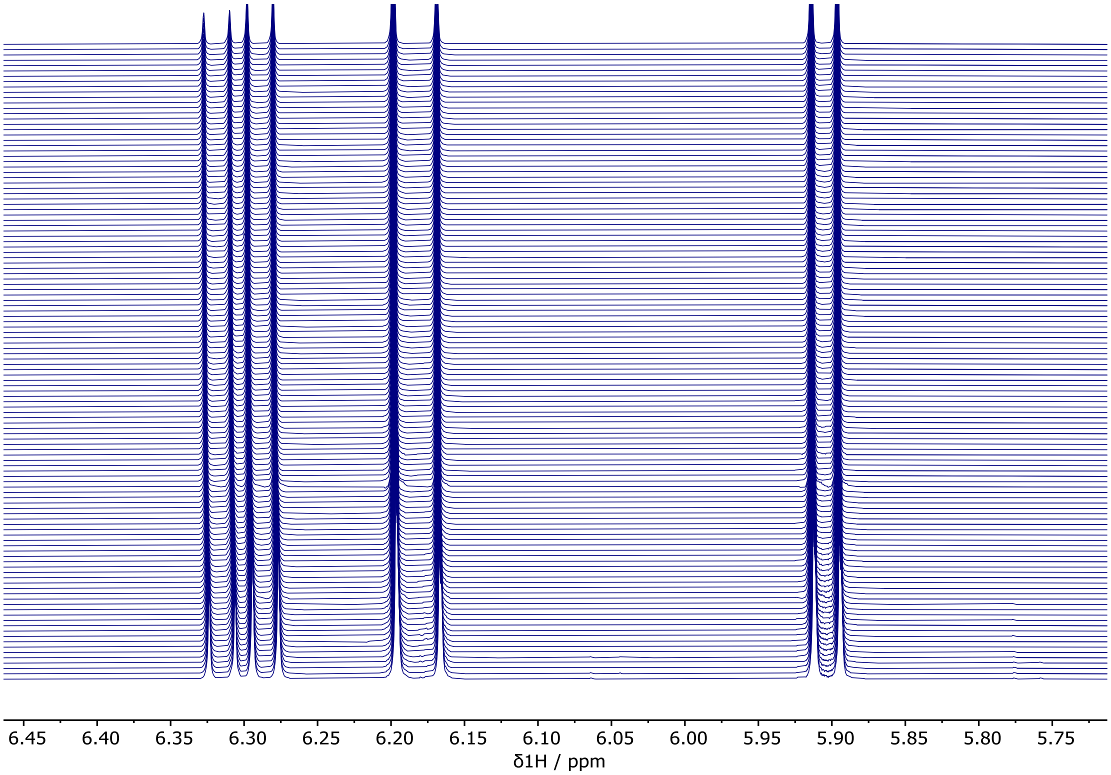

*Figure S15. Partial temporal ^{1}H NMR Spectra (600 MHz) of the DBU (0.360 mM) catalysed reaction of **Nu2**$_H$ (11.6 mM) and an excess of **E** (33.1 mM), in the presence of **C** (0.689 mM) over the course of 1 hour, showing the consumption of substrate **E**.*

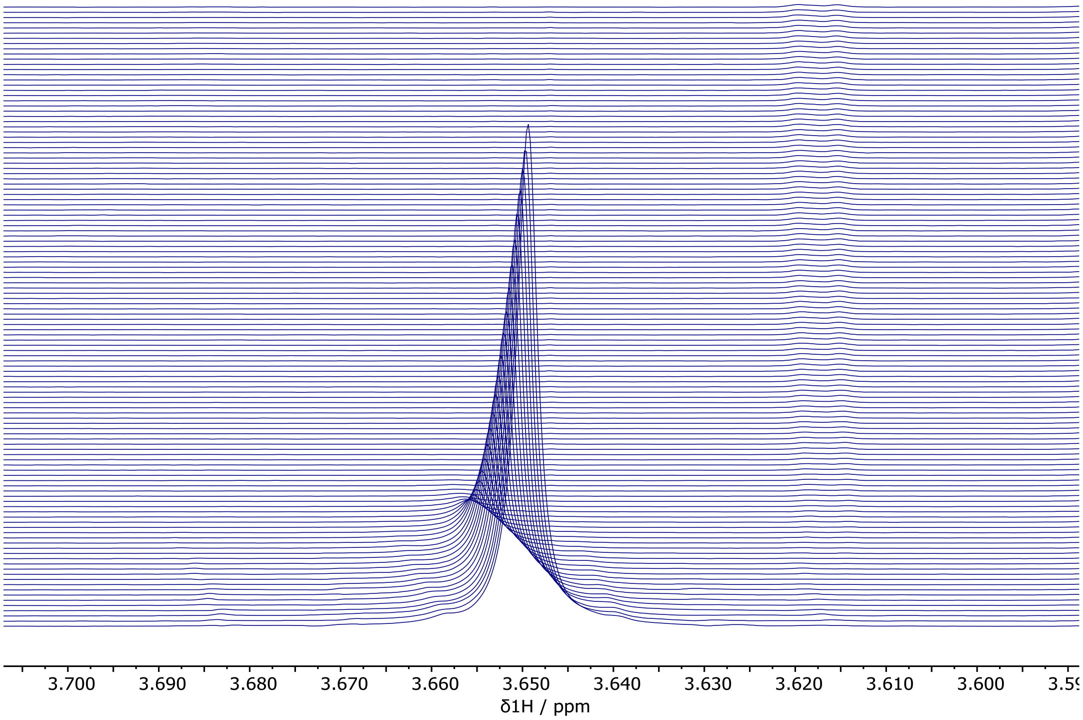

*Figure S16. Partial temporal ^{1}H NMR Spectra (600 MHz) of the DBU (0.360 mM) catalysed reaction of **Nu2**$_H$ (11.6 mM) and an excess of **E** (33.1 mM), in the presence of **C** (0.689 mM) over the course of 1 hour showing the consumption of limiting substrate **Nu2**$_H$.*

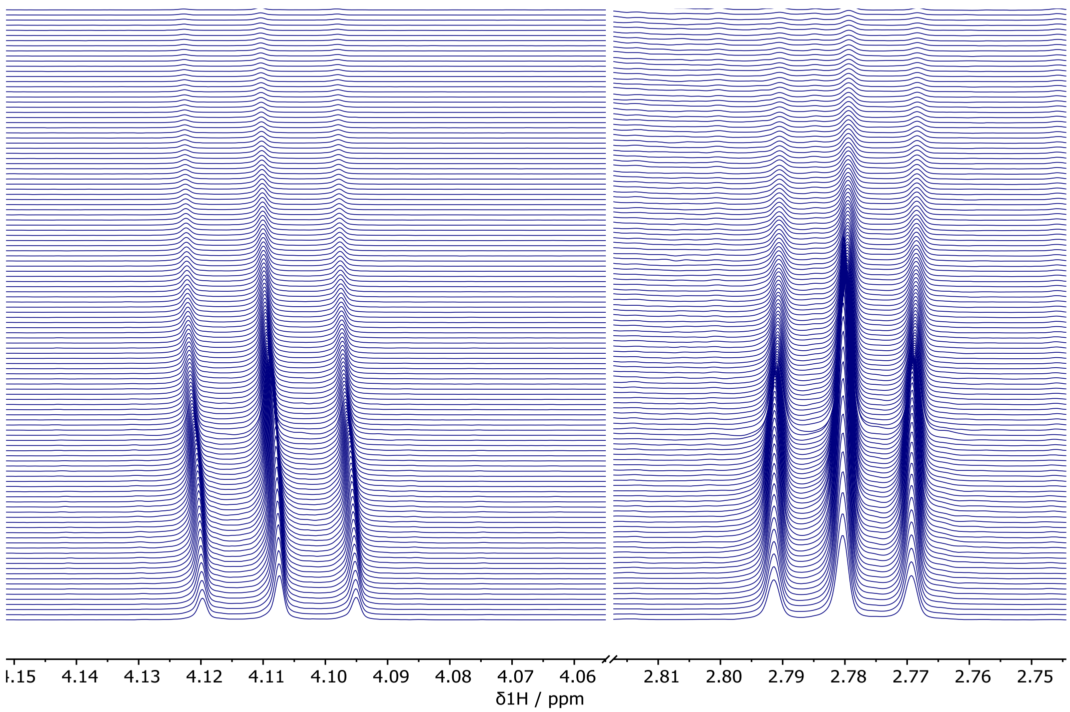

*Figure S17. Partial temporal ^{1}H NMR Spectra (600 MHz) of the DBU (0.360 mM) catalysed reaction of **Nu2**$_{H}$ (11.6 mM) and an excess of **E** (33.1 mM), in the presence of **C** (0.689 mM) over the course of 1 hour showing the formation and consumption of product/intermediate **P2**$_{H}$.*

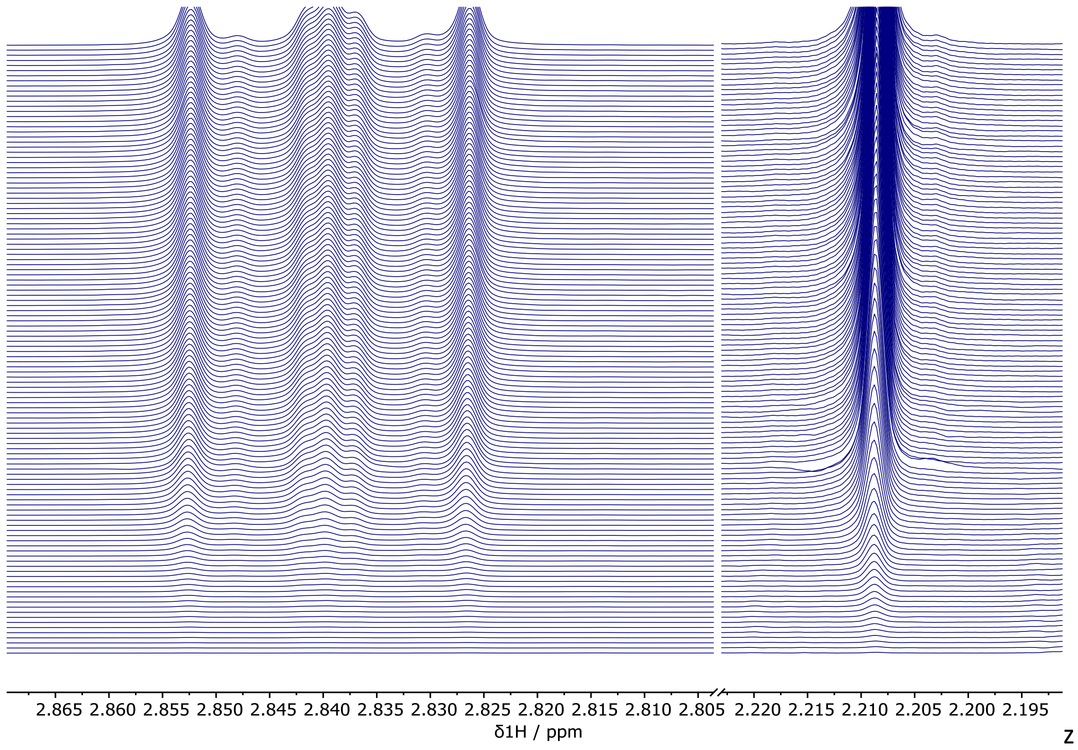

*Figure S18. Partial temporal ^{1}H NMR Spectra (600 MHz) of the DBU (0.360 mM) catalysed reaction of **Nu2**$_{H}$ (11.6 mM) and an excess of **E** (33.1 mM), in the presence of **C** (0.689 mM) over the course of 1 hour showing the formation and consumption of product **P3**.*

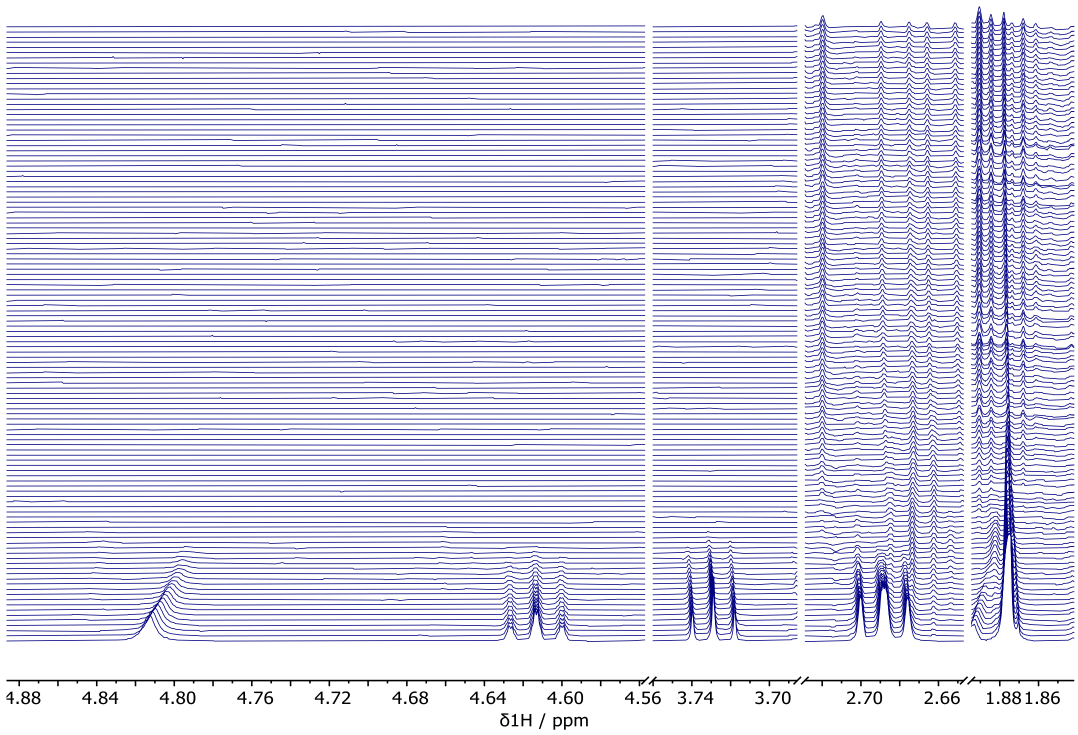

*Figure S19. Partial temporal ^{1}H NMR Spectra (600 MHz) of the DBU (0.360 mM) catalysed reaction of **Nu2**$_H$ (11.6 mM) and an excess of **E** (33.1 mM), in the presence of **C** (0.689 mM) over the course of 1 hour showing the consumption of intermediate **P2'**$_H$.*

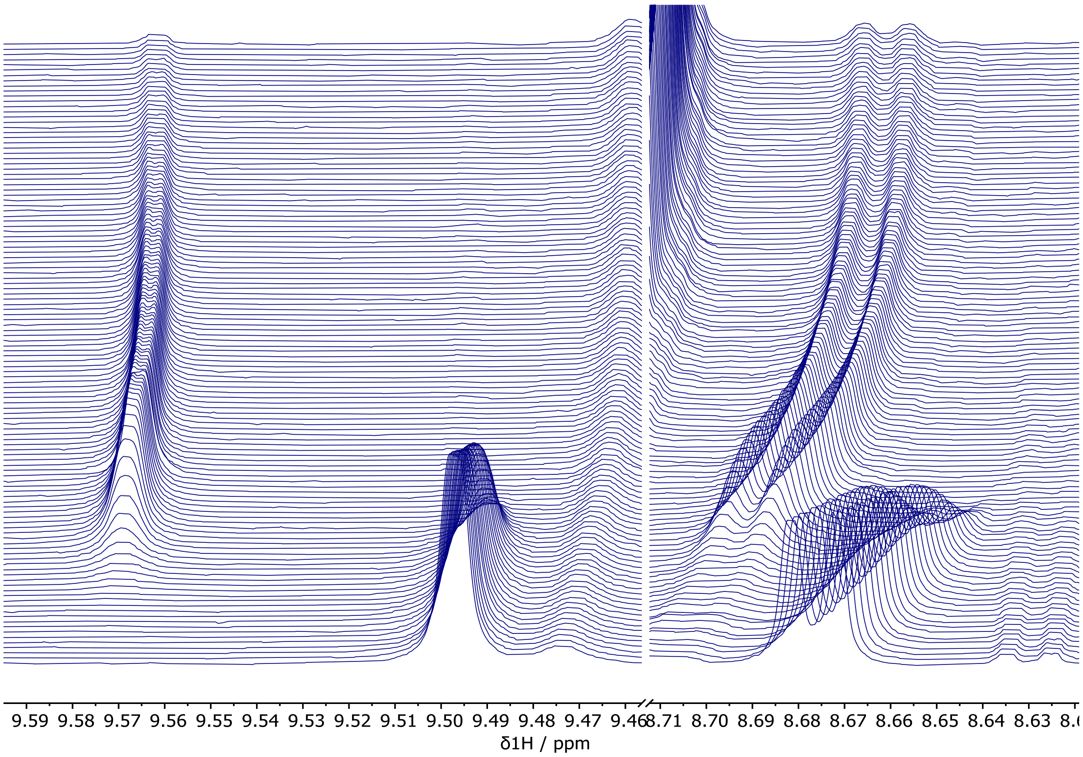

*Figure S20. Partial temporal ^{1}H NMR Spectra (600 MHz) of the DBU (0.360 mM) catalysed reaction of **Nu2**$_H$ (11.6 mM) and an excess of **E** (33.1 mM), in the presence of **C** (0.689 mM) over the course of 1 hour showing the C-derived intermediates **Nu2**$^-$⊂**C** and **P2**$^-$⊂C.*

S4.2.2.2 $Nu2_H$ Excess

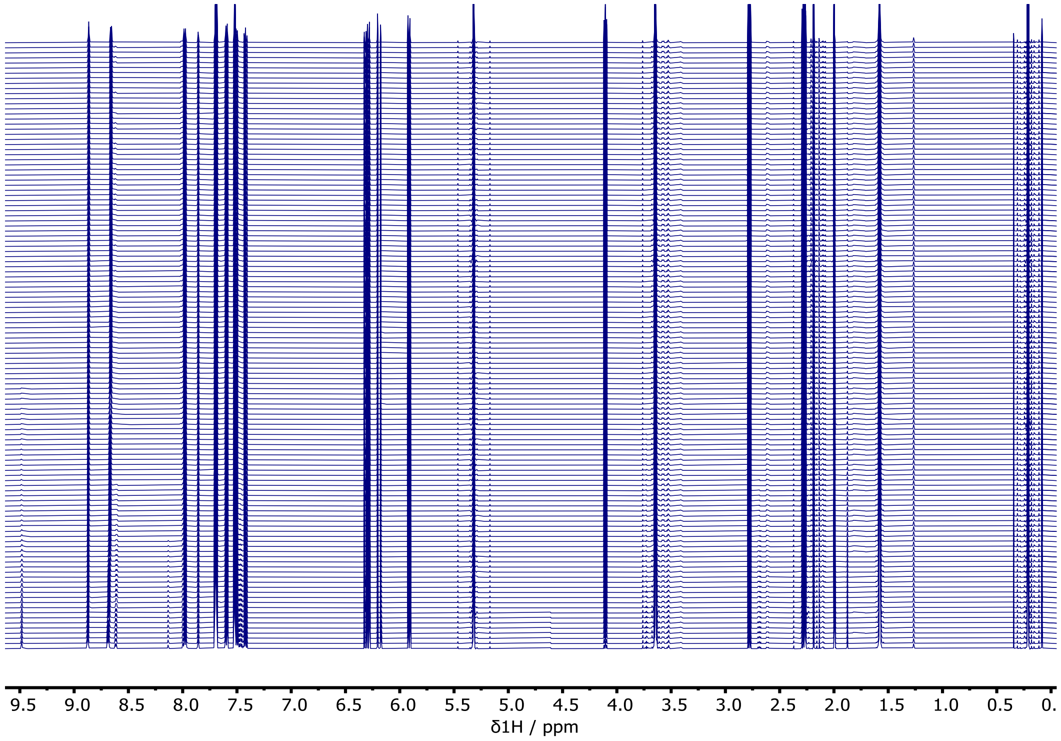

*Figure S21. Temporal ^{1}H NMR Spectra (600 MHz) of the DBU (0.337 mM) catalysed reaction of **E** (12.4 mM) and an excess of $\mathbf{Nu2}_H$ (34.9 mM), in the presence of **C** (0.840 mM) over the course of 1 hour.*

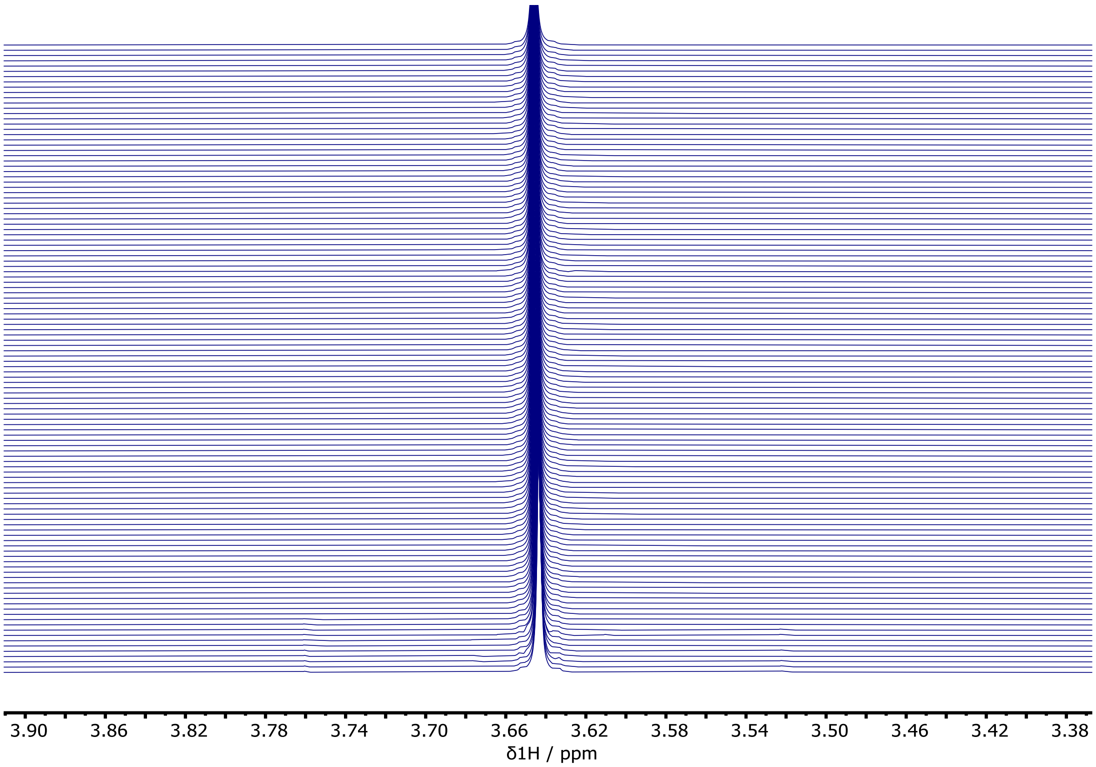

*Figure S22. Partial temporal ^{1}H NMR Spectra (600 MHz) of the DBU (0.337 mM) catalysed reaction of **E** (12.4 mM) and an excess of $\mathbf{Nu2}_H$ (34.9 mM), in the presence of **C** (0.840 mM) over the course of 1 hour showing the consumption of $\mathbf{Nu2}_H$.*

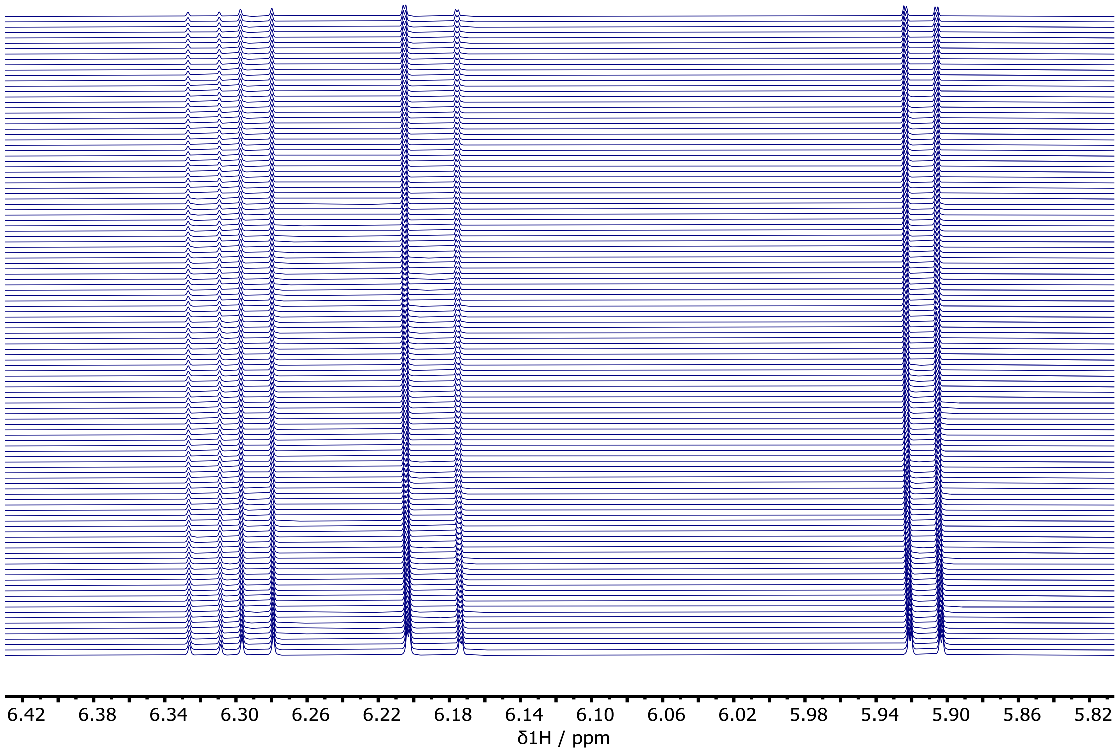

Figure S23. Partial temporal [1]*H NMR Spectra (600 MHz) of the DBU (0.337 mM) catalysed reaction of* ***E*** *(12.4 mM) and an excess of* ***Nu2***$_H$ *(34.9 mM), in the presence of* ***C*** *(0.840 mM) over the course of 1 hour showing the consumption of* ***E****.*

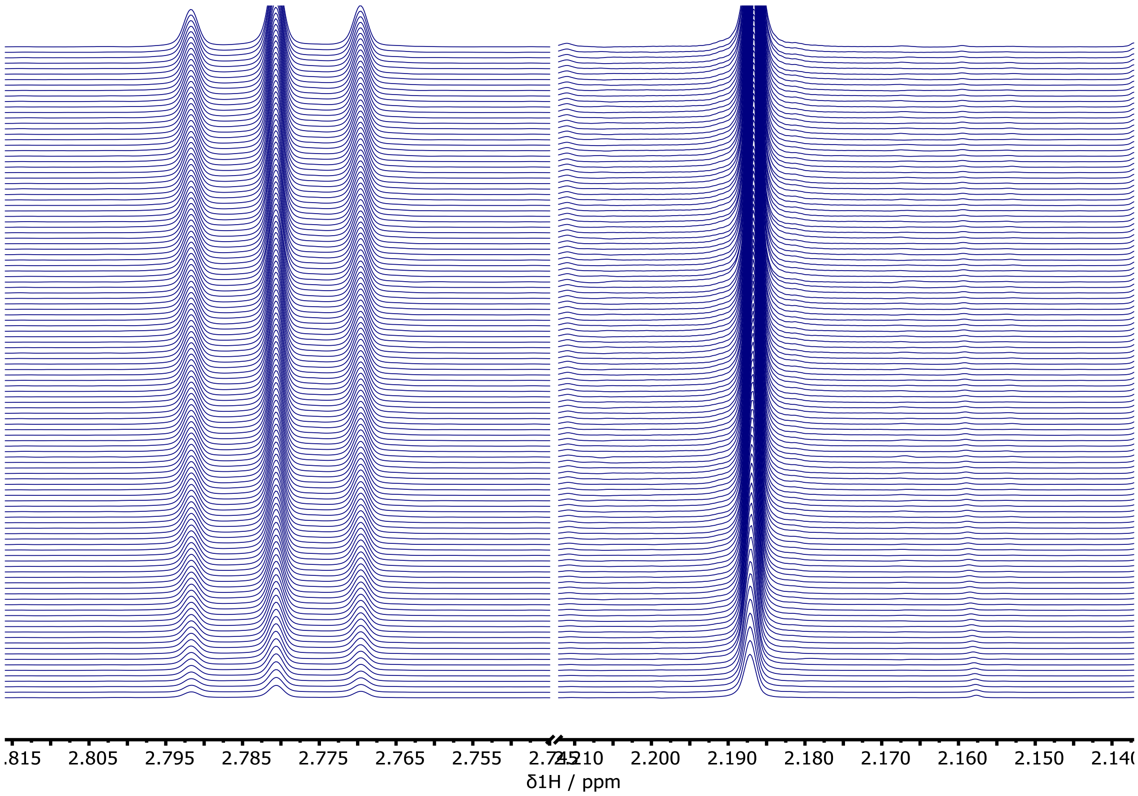

Figure S24. Partial temporal [1]*H NMR Spectra (600 MHz) of the DBU (0.337 mM) catalysed reaction of* ***E*** *(12.4 mM) and an excess of* ***Nu2***$_H$ *(34.9 mM), in the presence of* ***C*** *(0.840 mM) over the course of 1 hour showing the formation of* ***P2***$_H$*.*

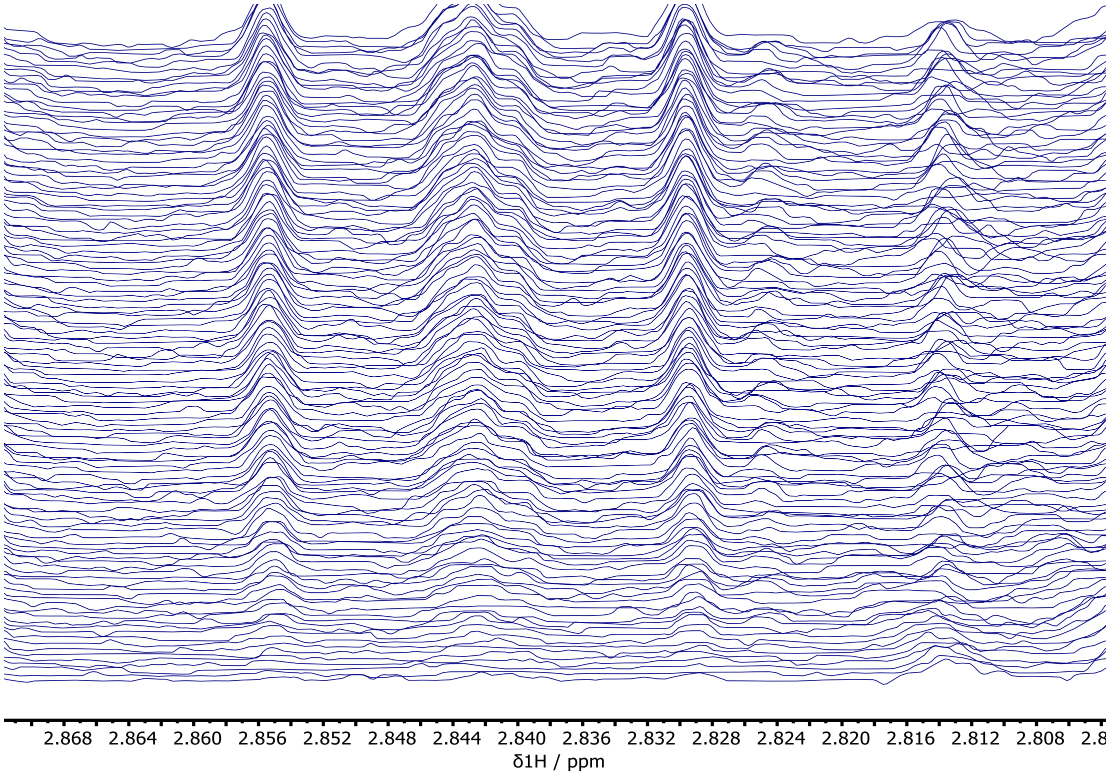

*Figure S25. Partial temporal ^{1}H NMR Spectra (600 MHz) of the DBU (0.337 mM) catalysed reaction of **E** (12.4 mM) and an excess of **Nu2**$_H$ (34.9 mM), in the presence of **C** (0.840 mM) over the course of 1 hour showing the formation of **P3**.*

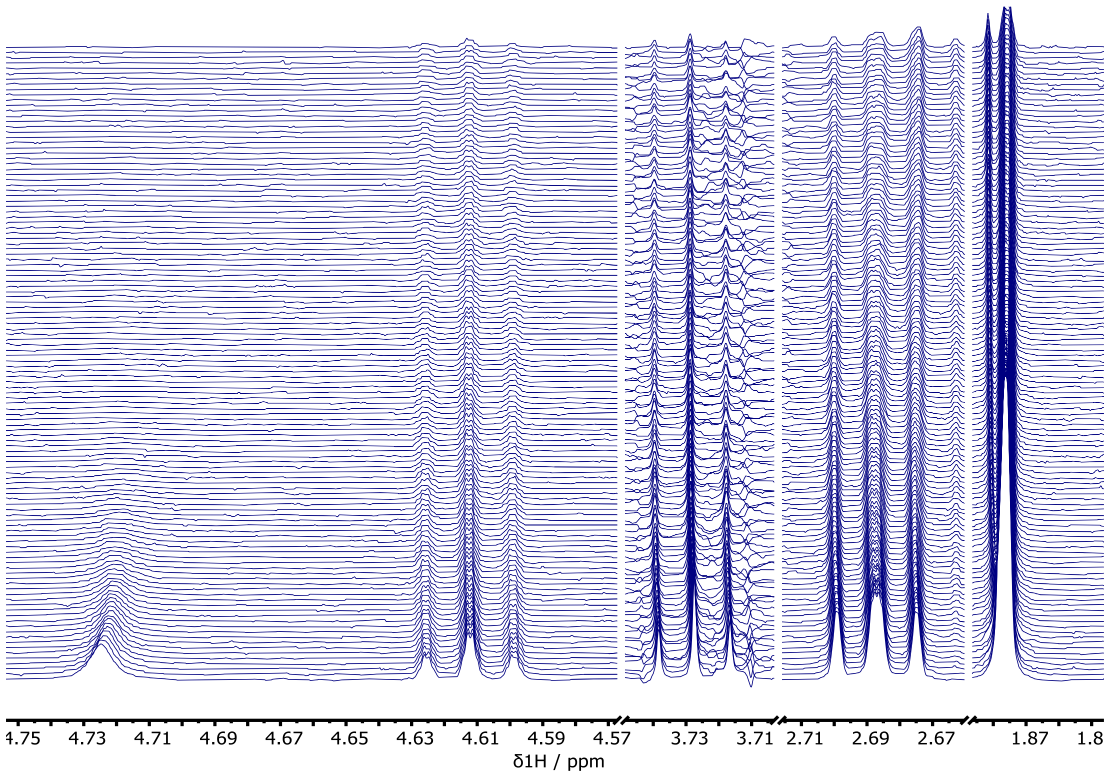

*Figure S26. Partial temporal ^{1}H NMR Spectra (600 MHz) of the DBU (0.337 mM) catalysed reaction of **E** (12.4 mM) and an excess of **Nu2**$_H$ (34.9 mM), in the presence of **C** (0.840 mM) over the course of 1 hour showing the consumption of **P2'**$_H$.*

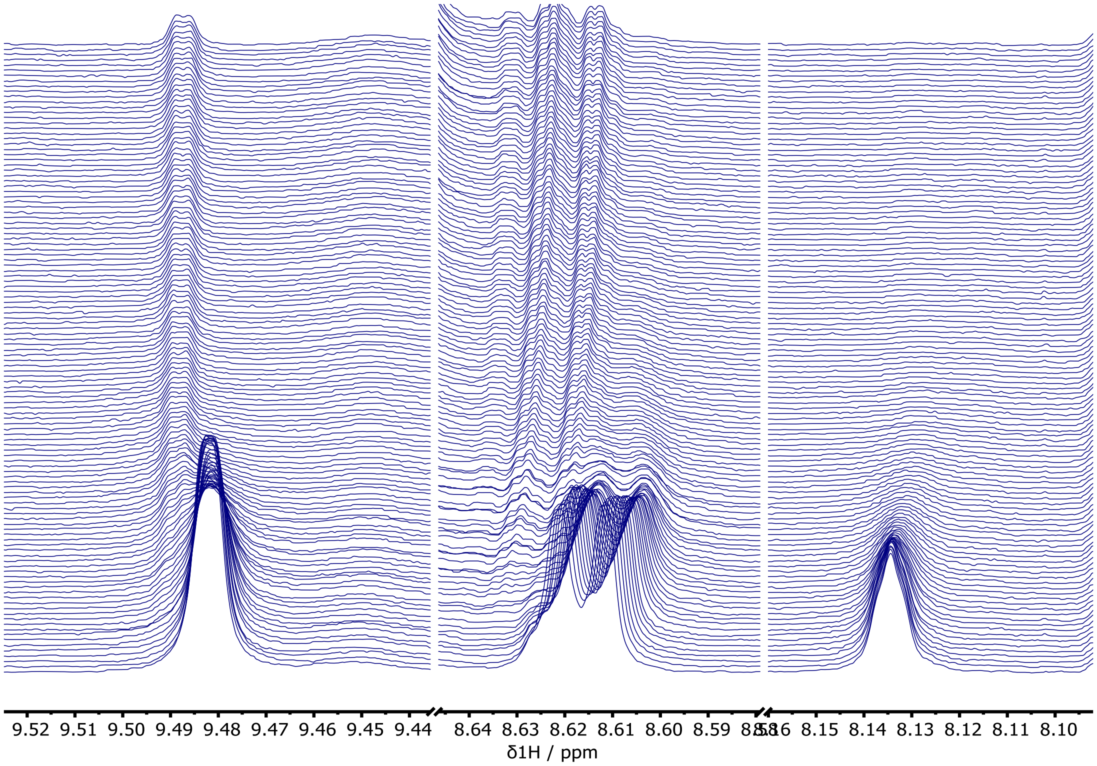

*Figure S27. Partial temporal ¹H NMR Spectra (600 MHz) of the DBU (0.337 mM) catalysed reaction of **E** (12.4 mM) and an excess of **Nu2**$_H$ (34.9 mM), in the presence of **C** (0.840 mM) over the course of 1 hour showing the consumption of **Nu1**$^-$⊂**C**.*

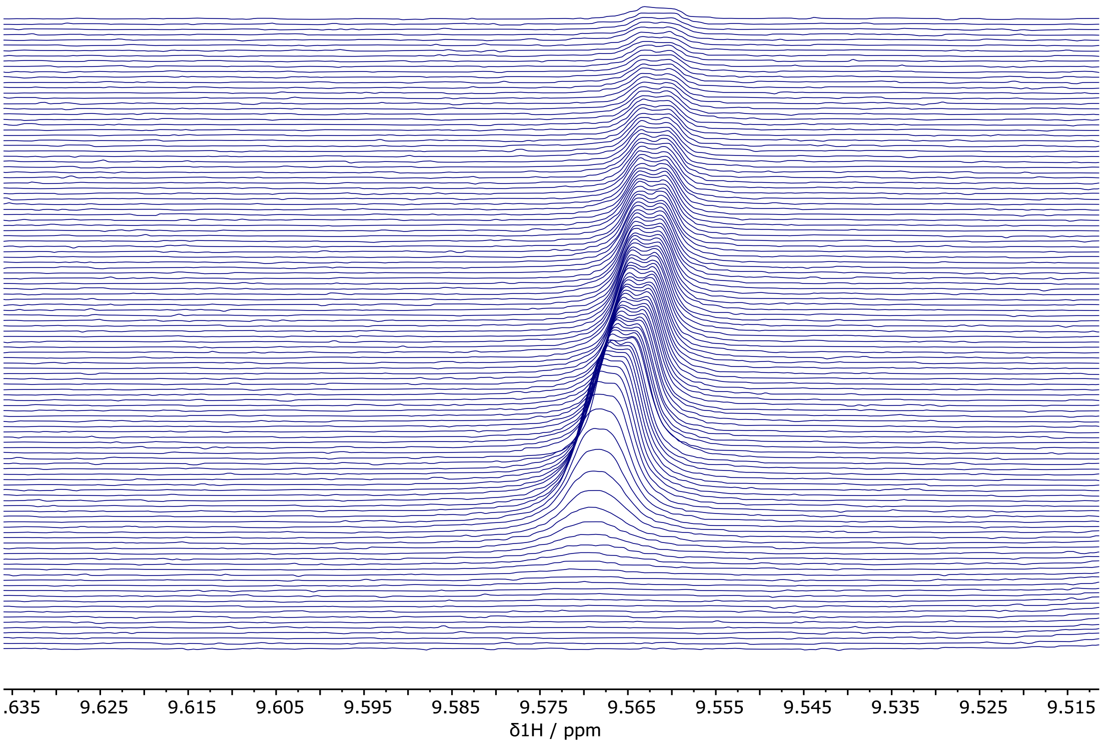

*Figure S28. Partial temporal ¹H NMR Spectra (600 MHz) of the DBU (0.337 mM) catalysed reaction of **E** (12.4 mM) and an excess of **Nu2**$_H$ (34.9 mM), in the presence of **C** (0.840 mM) over the course of 1 hour showing the formation and consumption of **P2**$^-$⊂**C**.*

S4.3 Temporal concentrations

S4.3.1 Reaction 1

S4.3.1.1 Nu1$_H$ Excess

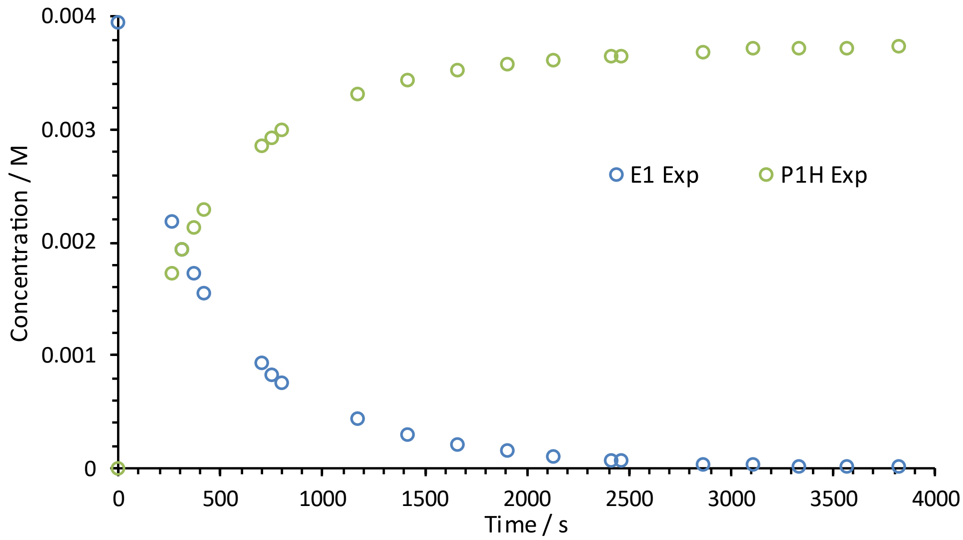

*Figure S29. Temporal concentrations of **Nu1**$_H$ and **E** derived from ^{1}H NMR reaction monitoring of reaction 1 under **Nu1**$_H$ excess conditions.*

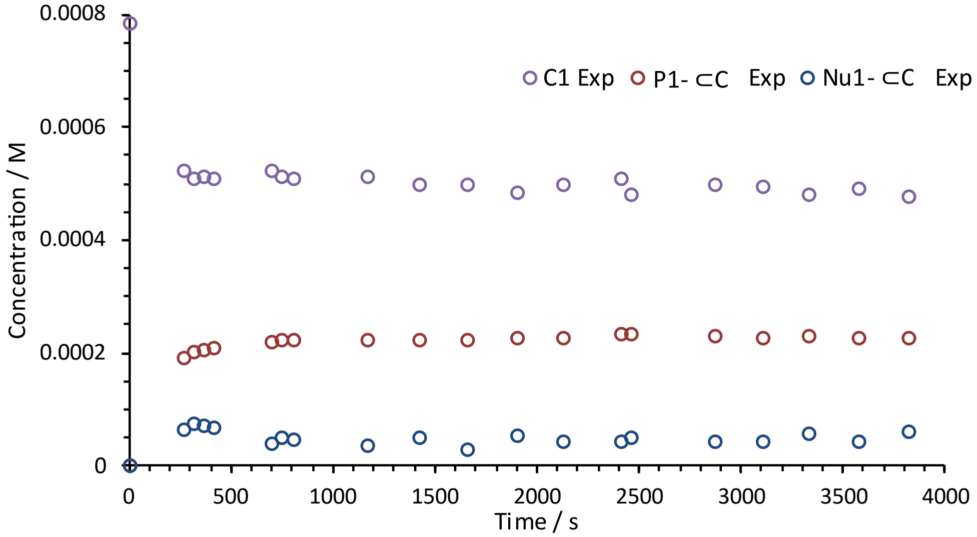

*Figure S30. Temporal concentrations of **C**, **Nu1**$^-$⊂**C** and **P1**$^-$⊂**C** derived from ^{1}H NMR reaction monitoring of reaction 1 under **Nu1**$_H$ excess conditions.*

S4.3.1.2 Equimolar

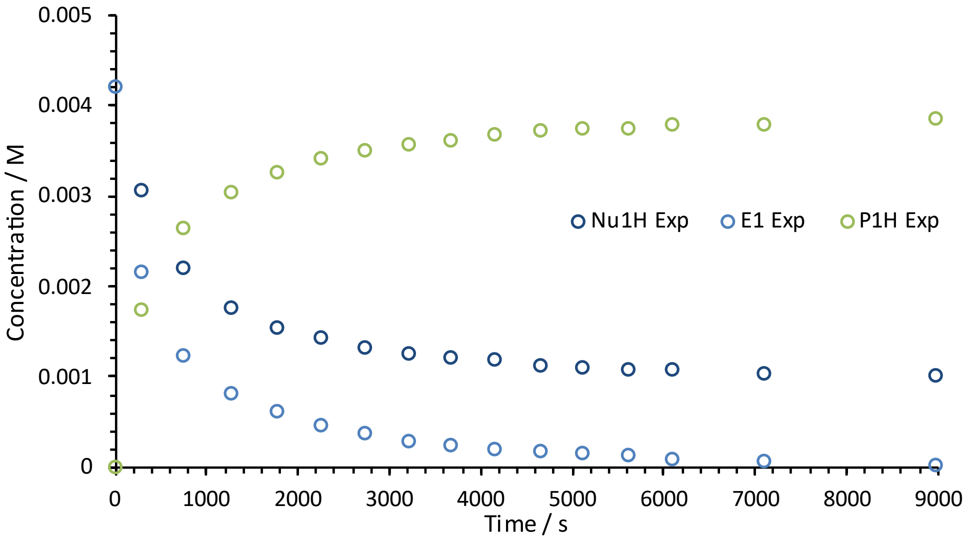

*Figure S31. Temporal concentrations of **Nu1**$_H$ and **E** derived from ^{1}H NMR reaction monitoring of reaction 1 under equimolar conditions.*

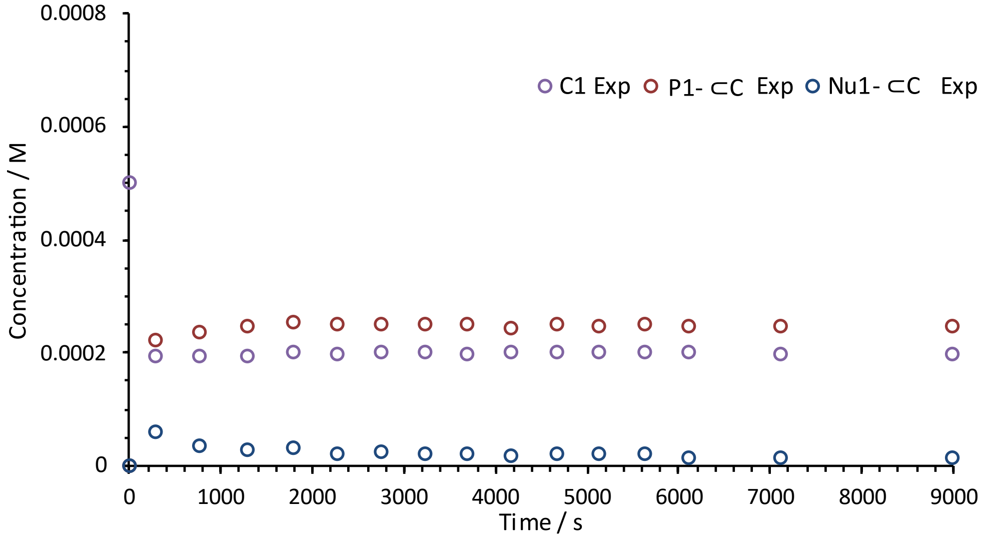

*Figure S32. Temporal concentrations of **C**, **Nu1**$^-$**⊂C** and **P1**$^-$**⊂C** derived from ^{1}H reaction monitoring of reaction 1 under equimolar conditions.*

S4.3.2 Reaction 2

S4.3.2.1 **E** Excess

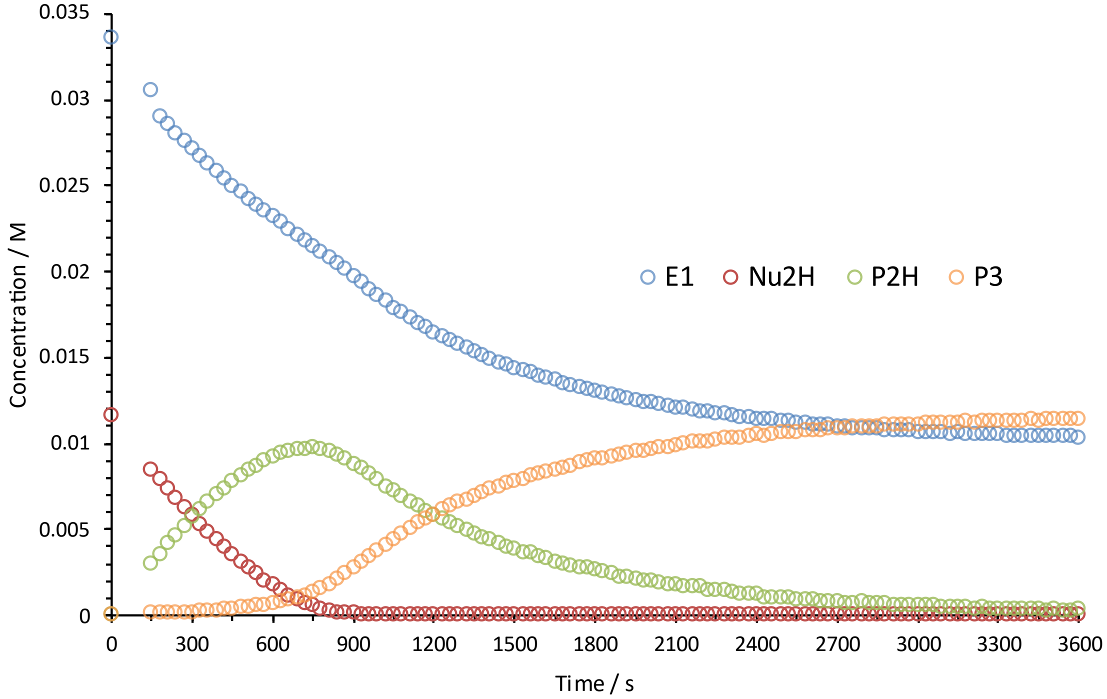

*Figure S33. Temporal concentrations of **Nu2**$_H$, **E**, **P2**$_H$, and **P3** derived from ^{1}H NMR reaction monitoring of reaction 2 under **E** excess conditions.*

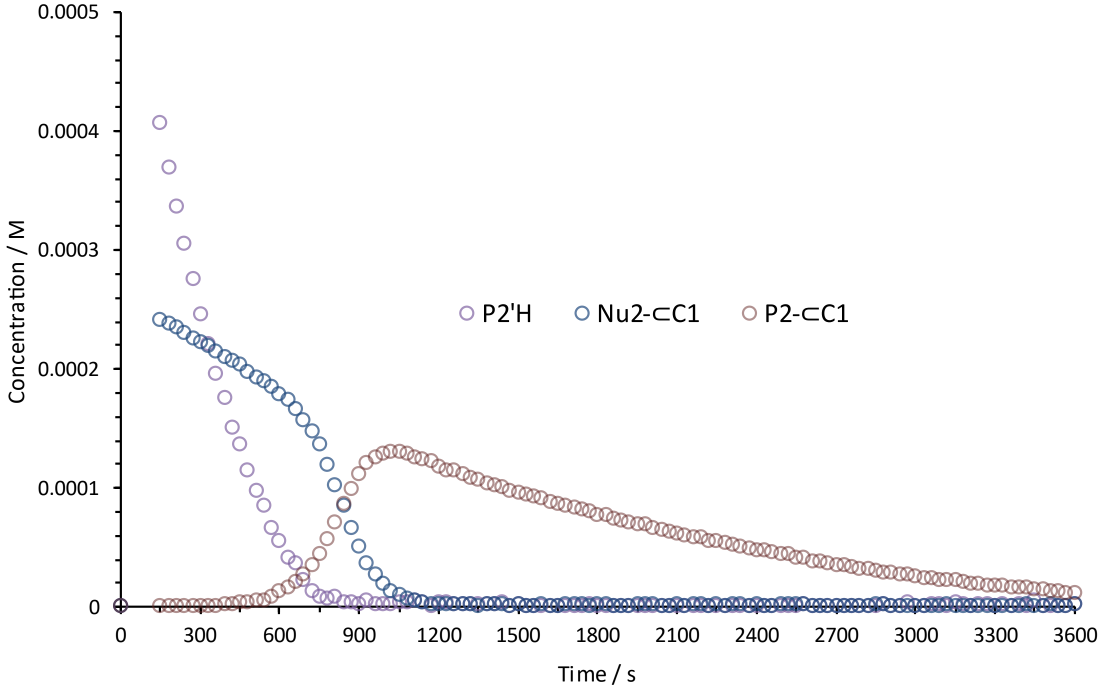

*Figure S34. Temporal concentrations of **P2'**$_H$, **Nu2$^-$⊂C** and **P2$^-$⊂C** derived from ^{1}H NMR reaction monitoring of reaction 2 under **E** excess conditions.*

S4.3.2.2 Nu2H Excess

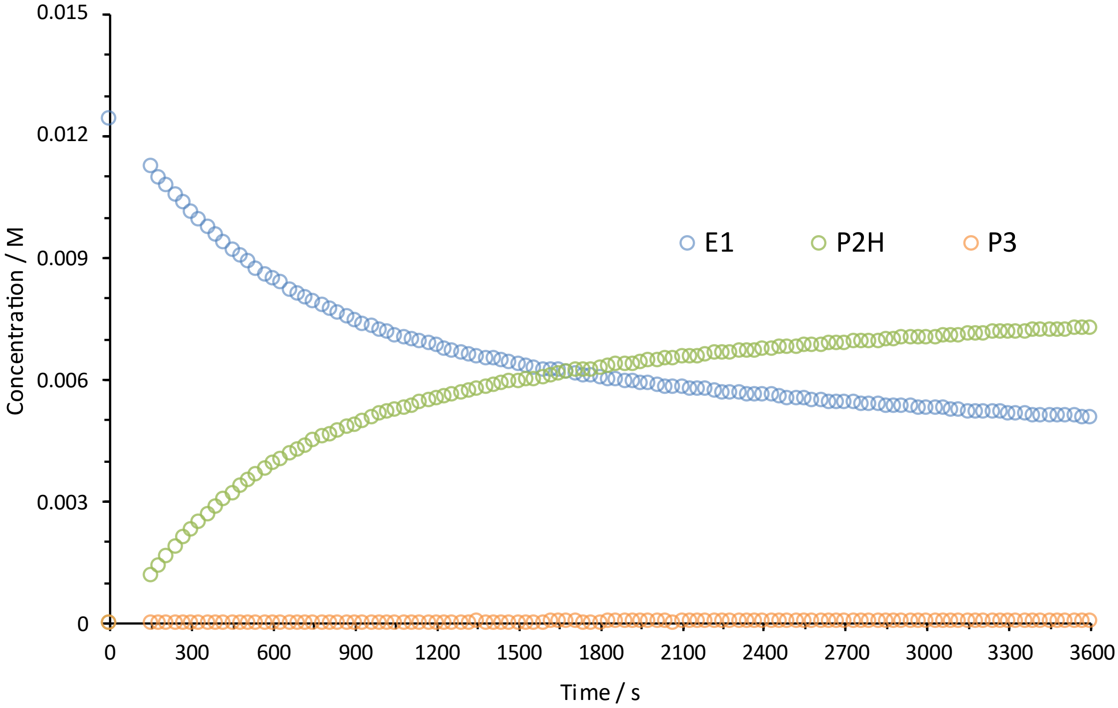

*Figure S35. Temporal concentrations of **Nu2**$_H$, **E**, **P2**$_H$, and **P3** derived from ^{1}H NMR reaction monitoring of reaction 2 under **Nu2**$_H$ excess conditions.*

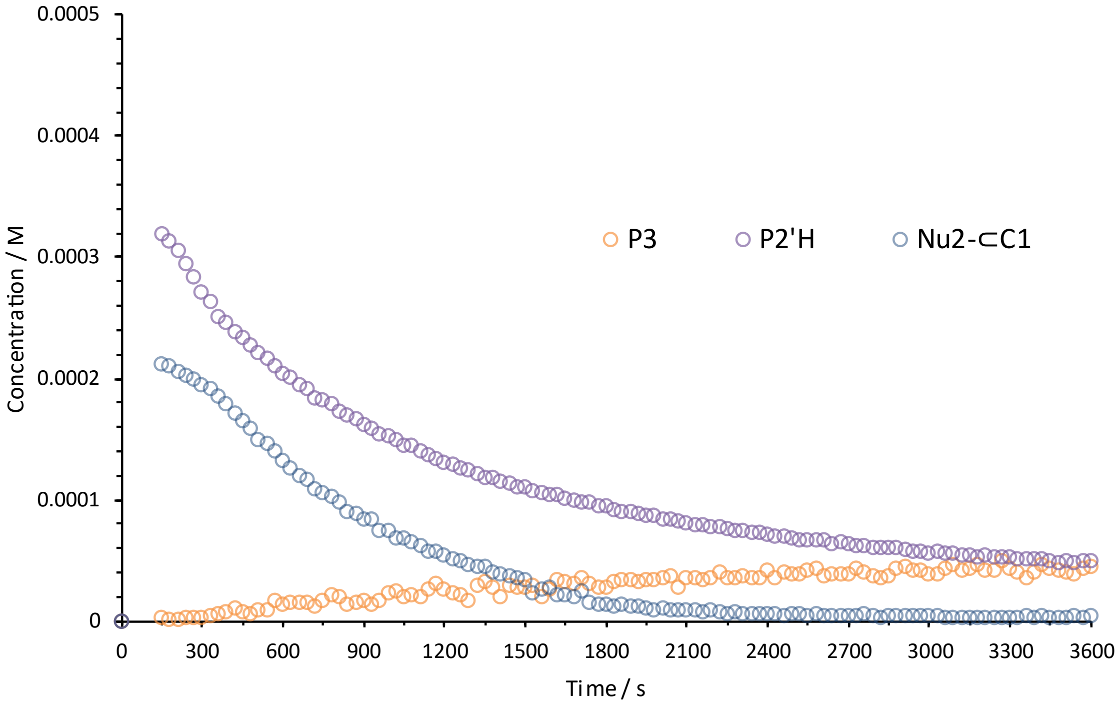

*Figure S36. Temporal concentrations of **P2'**$_H$, **Nu2**$^-$**⊂C** and **P2**$^-$**⊂C** derived from ^{1}H NMR reaction monitoring of reaction 2 under **Nu2**$_H$ excess conditions.*

S5. Intermediate characterisation

Directly detectable intermediates derived from **C** were characterised by comparison between ^1H NMR spectra from reaction monitoring and spectra from authentic generation of the intermediates using only the relevant components of the reaction system. **Nu1⁻⊂C** was prepared *in-situ* by addition of DBU to a solution of **C** and **Nu1**$_H$ in CD_2Cl_2, and **P1⁻⊂C** by addition of DBU to a solution of **P1**$_H$ and **C** in CD_2Cl_2 (Figure S37). Solutions of **Nu2⁻⊂C** and **P2⁻⊂C** were prepared in a similar manner.

S5.1 Nu1⁻⊂C and P1⁻⊂C

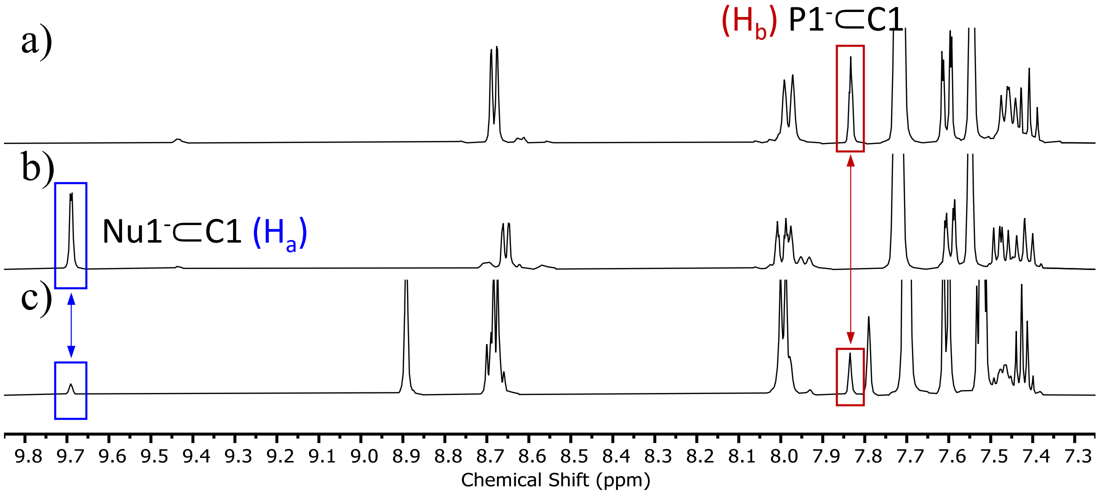

*Figure S37. Partial ^1H NMR spectra (600 MHz) of CD_2Cl_2 solutions of; a) **P1**$_H$ (excess), **C** (1 mM), and DBU (2 mM), b) **Nu1**$_H$ (excess), **C** (1 mM) and DBU (2 mM), c) **Nu1**$_H$, **E**, **P1**$_H$, **C** and DBU extracted from the reaction monitoring stacked spectra.*

S5.2 $Nu2^{-} \subset C$ and $P2^{-} \subset C$

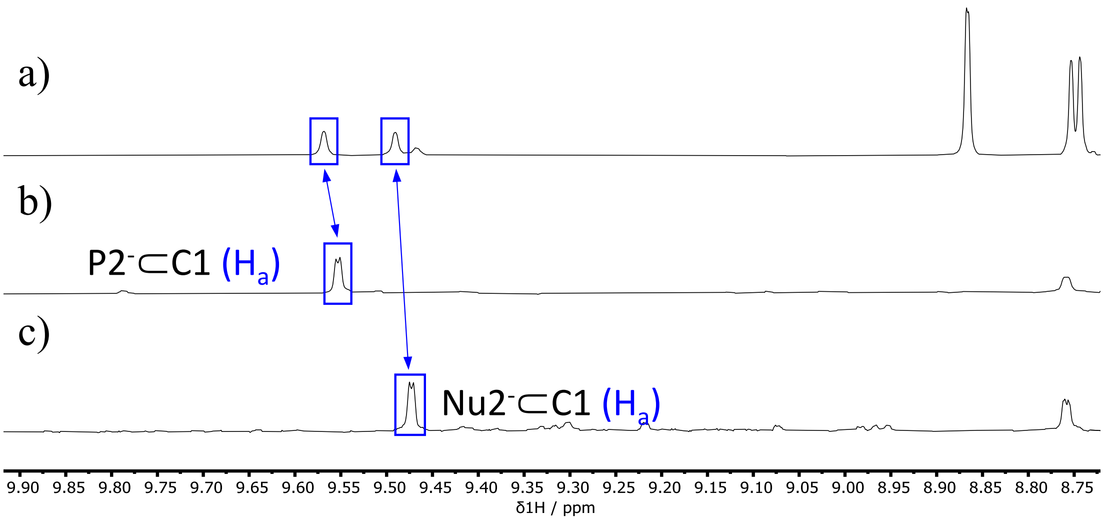

*Figure S38. Partial ^{1}H NMR spectra (600 MHz) of CD_2Cl_2 solutions of; a) **Nu2**$_H$, **E**, **P2**$_H$, **C** and DBU extracted from the reaction monitoring stacked spectra, b) **P2**$_H$ (excess), **C** (1 mM), and DBU (2 mM), c) **Nu2**$_H$ (excess), **C** (1 mM), and DBU (2 mM).*

S6. Kinetic modelling of cage-catalysed Michael addition reactions

Kinetic models were fit to datasets using the DynoChem 5 software package from Scale-up systems. For a given model, the shapes of the temporal concentration curves were calibrated by manually adjusting the values of the rate and equilibrium constants in the interactive simulator environment, before being optimised in the fitting environment using the Levenberg-Marquardt algorithm. For models involving >10 fitting parameters, the optimisations were simplified by fitting only subsets of parameters or subsets of concentrations, e.g. only fitting the intermediate concentrations or the rates for the addition reactions on the first pass, then integrating the substrates and products or the rates of other processes on the second pass. The fitted values of rate constants reported in this section reflect a simultaneous fitting to the temporal concentrations of species in all experimental datasets acquired in the case of each reaction. Kinetic parameters are described in tables accompanying each fitted model along with their units. Rate constants are denoted as k_n and equilibrium constants as K_n which are defined as $\frac{k_n}{k_{-n}}$ in all cases.

S6.1 Reaction 1 Models

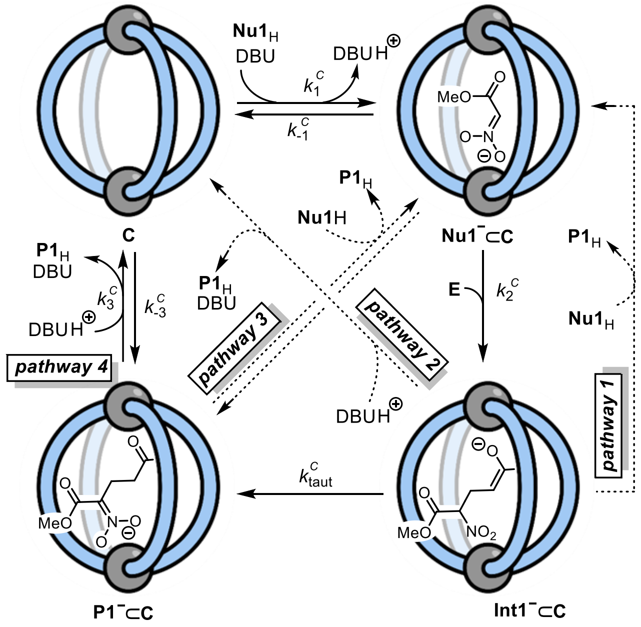

*Figure S39. All models considered in describing the kinetics of the DBU catalysed reaction between **Nu1**$_H$ and **E**, in the presence of **C**.*

*Table S3. Initial conditions for the reactions between **Nu1**$_H$ and **E** in the presence of **C** and DBU.*

Entry	$[E]_0$ / mM	$[Nu1_H]_0$ / mM	$[C]_0$ / mM	$[DBU]_0$ / mM
1	3.95	10.5	0.784	0.273
2	4.21	5.13	0.501	0.303

S6.1.1 Model 1

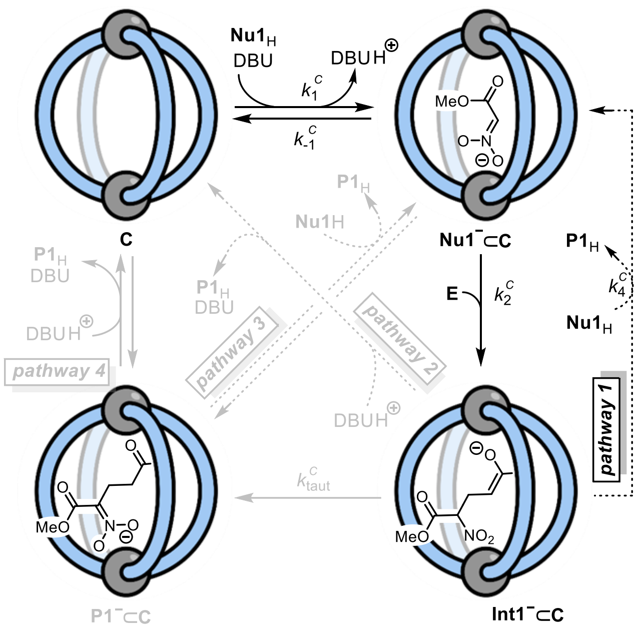

*Figure S40. Model 1 describing the DBU catalysed reaction between **Nu1**$_H$ and **E** in the presence of **C**.*

Table S4. Optimal kinetic and thermodynamic parameters fitted according to model 1.

Parameter / Units	Key relationships/thresholds
K_1^C / M^{-2}	$K_1^C > 10^4$
k_1^C / $M^{-2}s^{-1}$	$k_1^C > 10^4$
k_2^C / $M^{-1}s^{-1}$	$k_2^C = 5.95$

Associative deprotonation:

$$C + DBU + Nu1_H \underset{k_{-1}^{C}}{\overset{\mathrm{k}_1^C}{\rightleftharpoons}} Nu1^- \subset C$$

Conjugate addition:

$$E1 + Nu2^- \subset C \overset{k_2^C}{\rightarrow} Int^- \subset C$$

Direct displacement:

$$Nu1_H + P1^- \subset C \overset{k_4^C}{\rightarrow} P1_H + Nu1^- \subset C$$

S6.1.1.1 **Nu1**H Excess

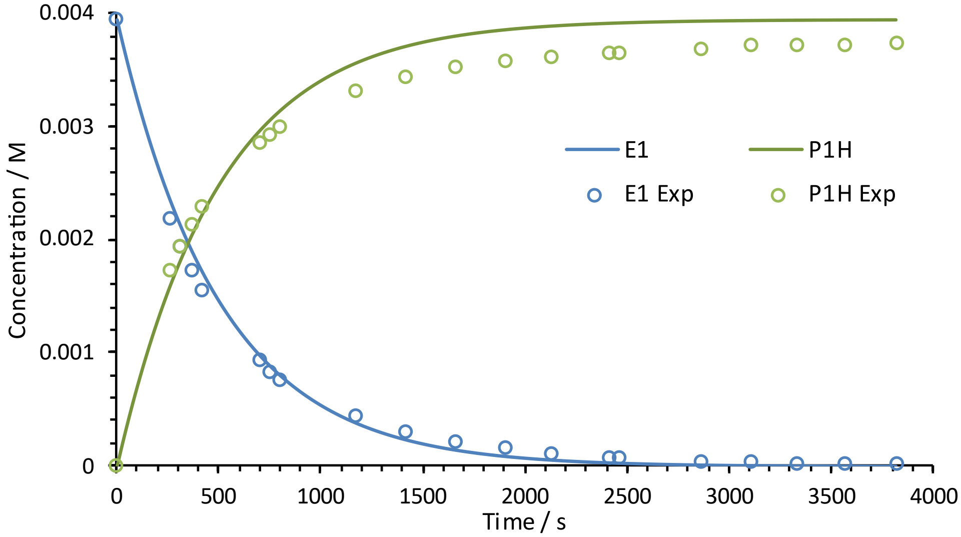

Figure S41. Experimental (open circles) and simulated (filled lines) temporal concentrations of limiting substrate and product where model 1 is used in the generation of the simulated data.

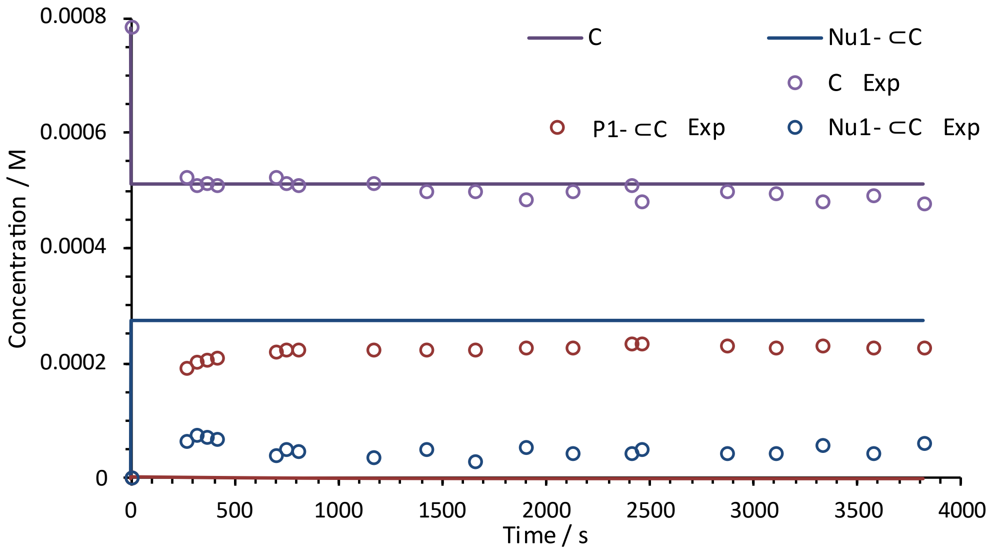

Figure S42. Experimental (open circles) and simulated (filled lines) temporal concentrations of ***C****-derived intermediates where model 1 is used in the generation of the simulated data.*

S6.1.1.2 Equimolar

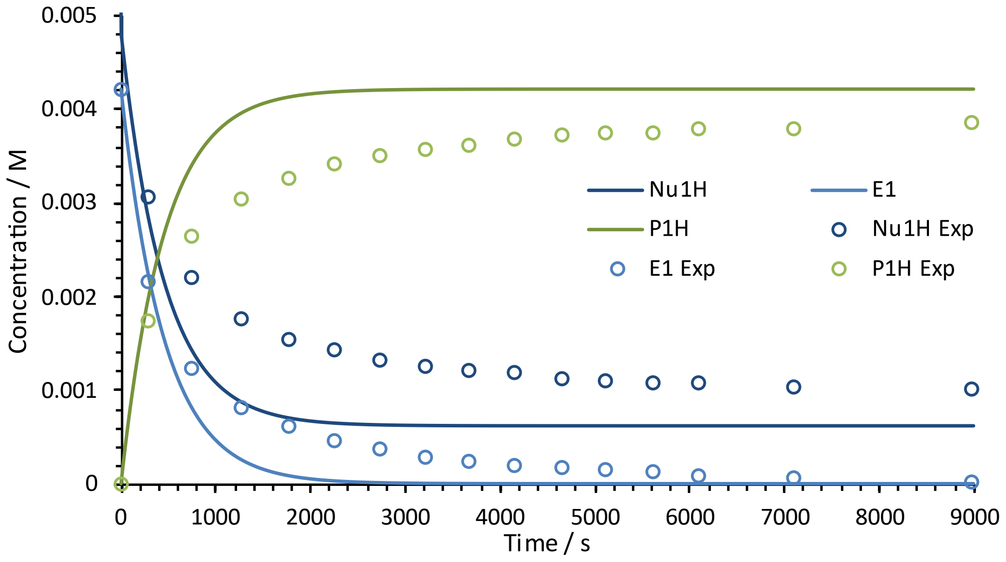

Figure S43. Experimental (open circles) and simulated (filled lines) temporal concentrations of limiting substrate and product where model 1 is used in the generation of the simulated data.

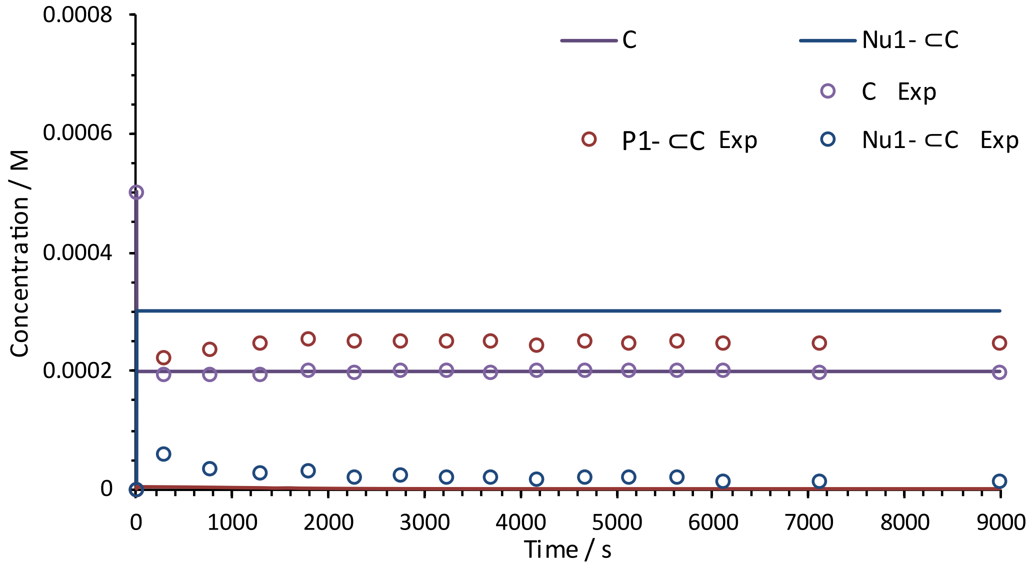

*Figure S44. Experimental (open circles) and simulated (filled lines) temporal concentrations of **C**-derived intermediates where model 1 is used in the generation of the simulated data.*

S6.1.2 Model 2

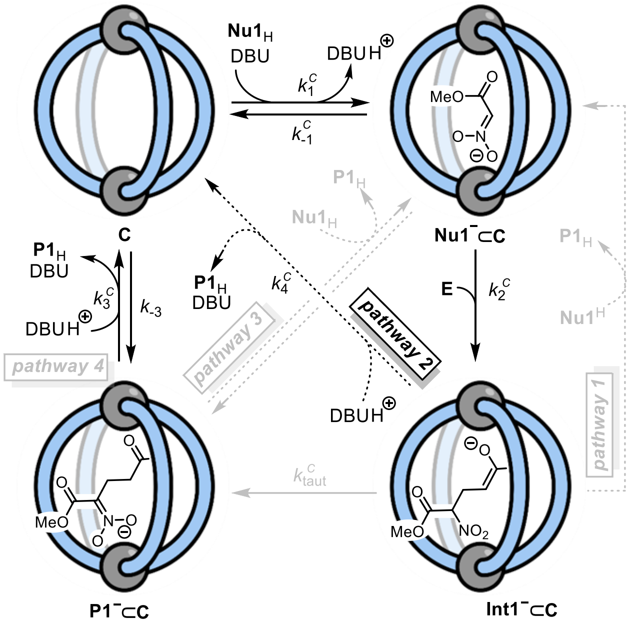

*Figure S45. Model 2 describing the DBU catalysed reaction between **Nu1**H and **E** in the presence of **C**.*

Table S5. Optimal kinetic and thermodynamic parameters fitted according to model 2.

Parameter / Units	Key thresholds/relationships
K_1^C / M^{-1}	$K_1^C > 100$
k_1^C / $M^{-2}s^{-1}$	$k_1^C > 5 \times 10^4$
k_2^C / M^{-1} s^{-1}	$k_2^C = 25.3$
K_{-3}^C / M^{-1}	$K_1^C / K_{-3}^C = 0.145$
k_{-3}^C / M^{-2} s^{-1}	$k_4^C > 5 \times 10^4$
k_4 / s^{-1}	$k_{taut}^C > 10$

Associative deprotonation:

$$C + DBU + Nu1_H \underset{k_{-1}^C}{\overset{k_1^C}{\rightleftharpoons}} Nu1^- \subset C$$

$$C + DBU + P1_H \underset{k_{-4}^C}{\overset{k_4^C}{\rightleftharpoons}} P1^- \subset C$$

Conjugate addition:

$$E1 + Nu2^{-} \subset C \overset{k_2^C}{\rightarrow} Int1^{-} \subset C$$

Direct deprotonation:

$$Int1^{-} \subset C \overset{k_3^C}{\rightarrow} P1_H + C + DBU$$

S6.1.2.1 **Nu1**$_H$ Excess

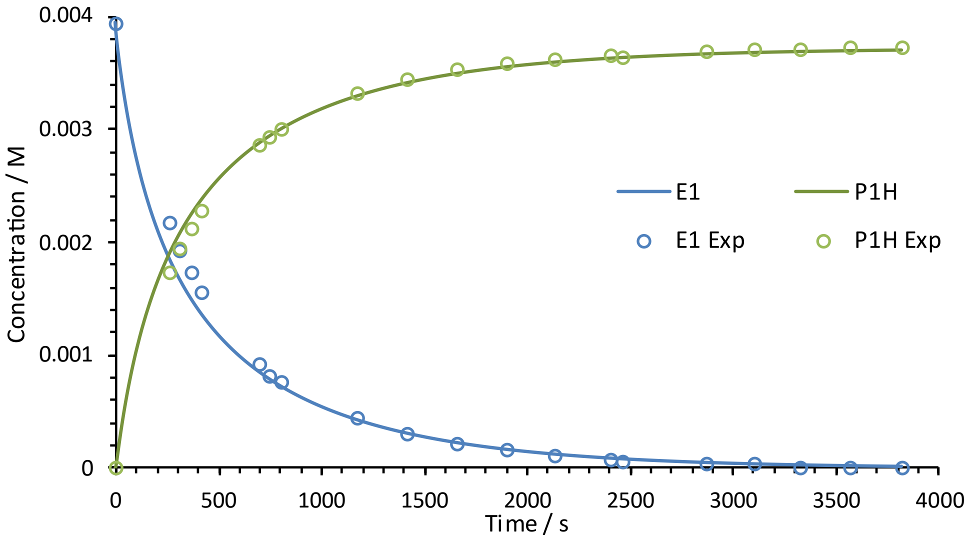

Figure S46. Experimental (open circles) and simulated (filled lines) temporal concentrations of limiting substrate and product where model 2 is used in the generation of the simulated data.

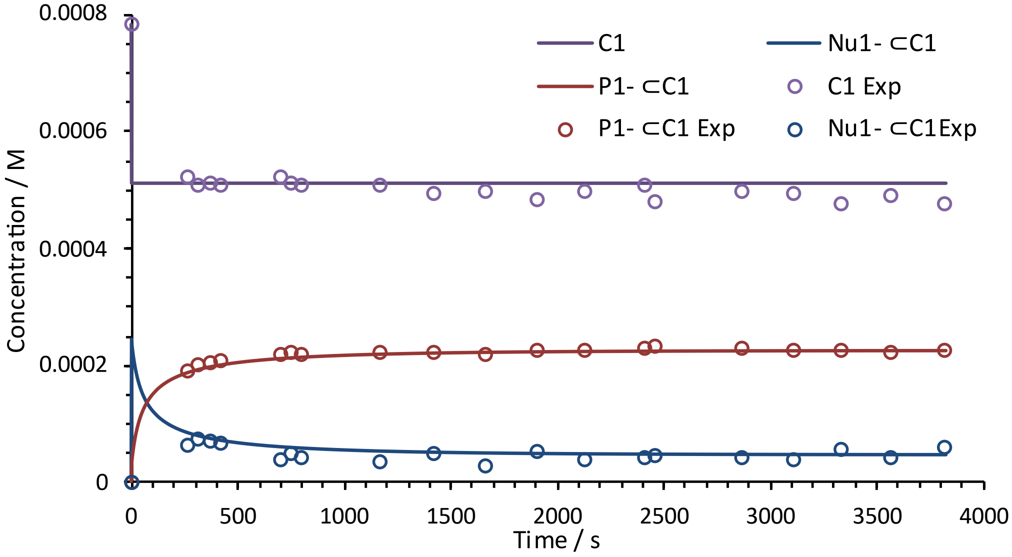

*Figure S47. Experimental (open circles) and simulated (filled lines) temporal concentrations of **C**-derived intermediates model 2 is used in the generation of the simulated data.*

S6.1.2.2 Equimolar

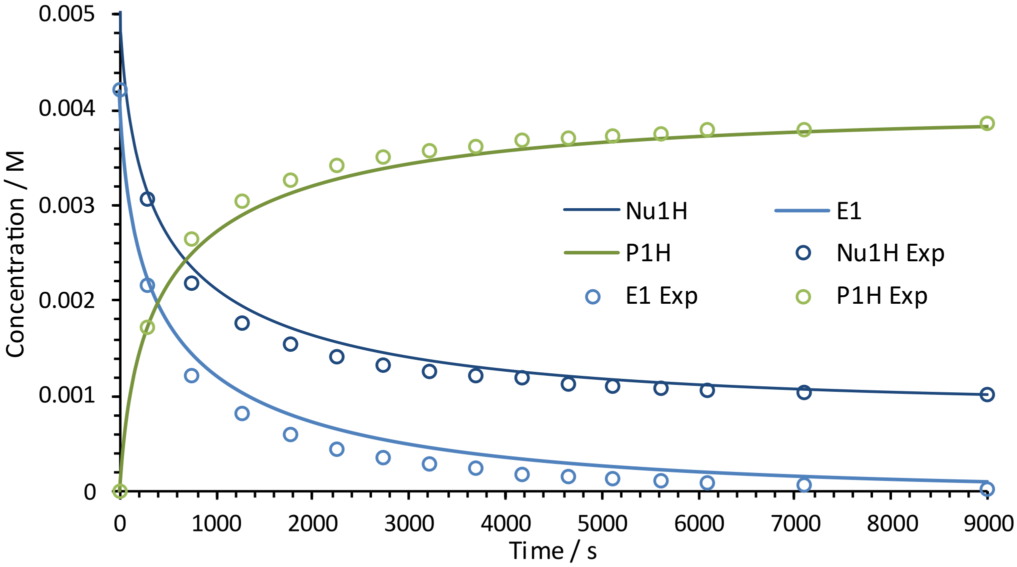

Figure S48. Experimental (open circles) and simulated (filled lines) temporal concentrations of limiting substrate and product where model 1 is used in the generation of the simulated data.

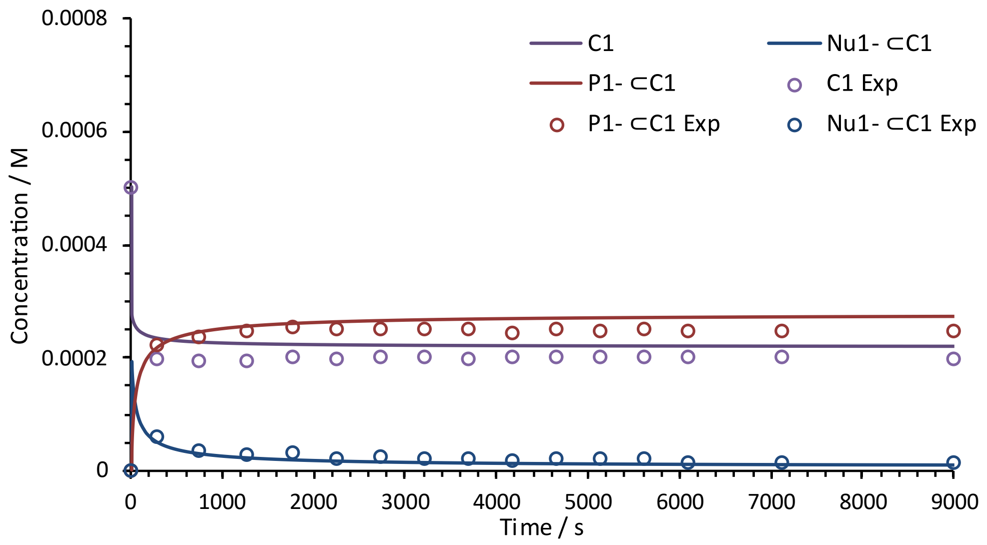

Figure S49. Experimental (open circles) and simulated (filled lines) temporal concentrations of limiting substrate and product where model 2 is used in the generation of the simulated data.

S6.1.3 Model 3

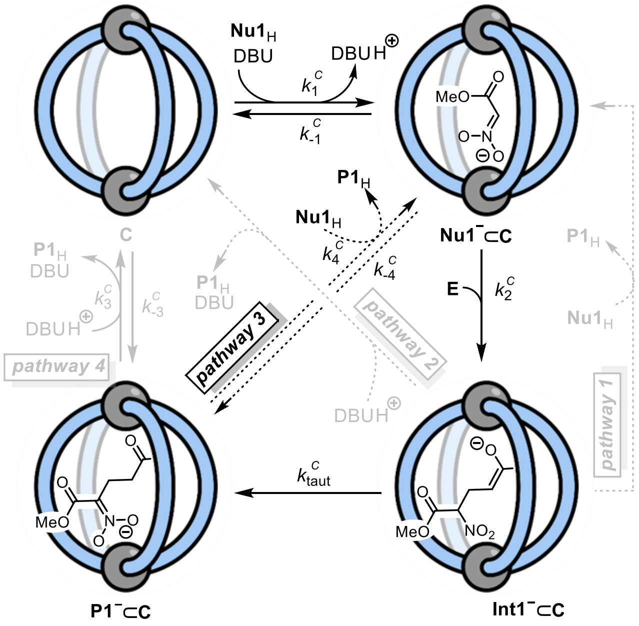

*Figure S50. Model 3 describing the DBU catalysed reaction between **Nu1**$_H$ and **E** in the presence of **C**.*

Table S6. Optimal kinetic and thermodynamic parameters fitted according to model 3.

Parameter / Units	Key thresholds/relationships
K_1^C / M^{-1}	$K_1^C > 5 \times 10^2$
k_1^C / $M^{-2}s^{-1}$	$k_1^C > 5 \times 10^3$
k_2^C / M^{-1} s^{-1}	$k_2^C = 23.9$
K_4^C / –	$K_4^C = 0.114$
k_4^C / M^{-1} s^{-1}	$k_4^C > 10$
k_{taut}^C / s^{-1}	$k_{taut}^C > 1$

Associative deprotonation:

$$C + DBU + Nu1_H \underset{k_{-1}^C}{\overset{k_1^C}{\rightleftharpoons}} Nu1^- \subset C$$

Conjugate addition:

$$E1 + Nu2^- \subset C \xrightarrow{k_2^C} Int1^- \subset C$$

Direct displacement:

$$Nu1_H + P1^- \subset C \underset{k_{-4}^C}{\overset{k_4^C}{\rightleftharpoons}} P1_H + Nu1^- \subset C$$

Tautomerisation:

$$Int1^- \subset C \xrightarrow{k_{taut}^C} P1^- \subset C$$

S6.1.3.1 **Nu1**$_H$ Excess

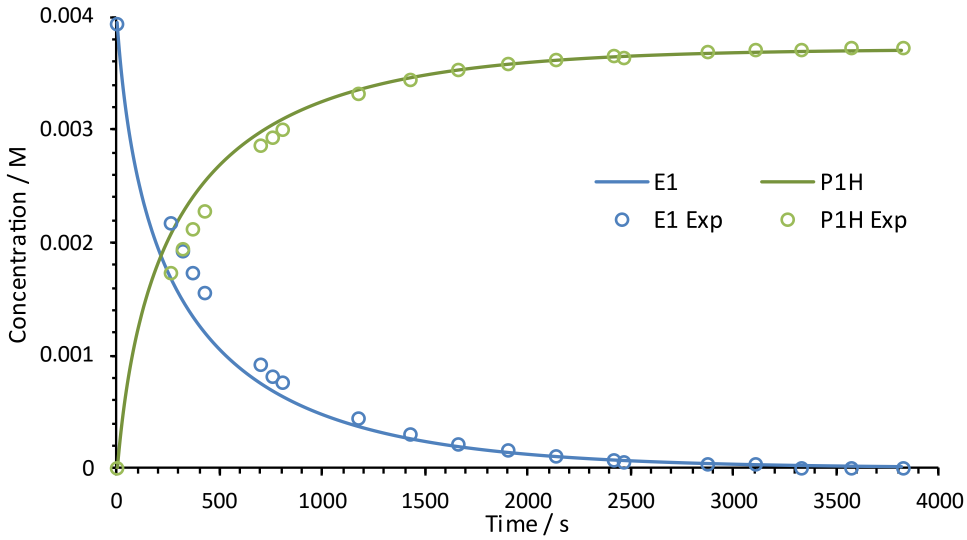

Figure S51. Experimental (open circles) and simulated (filled lines) temporal concentrations of limiting substrate and product where model 3 is used in the generation of the simulated data.

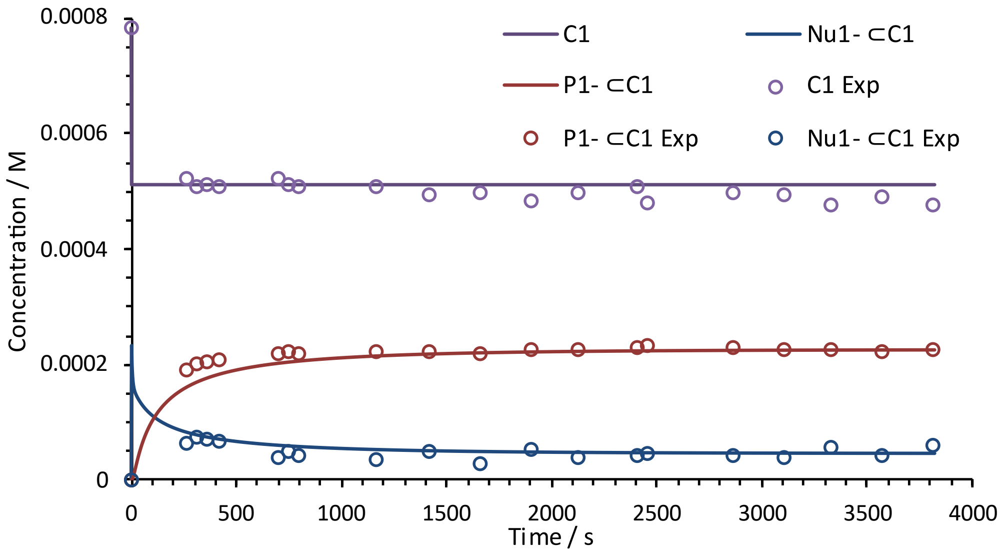

*Figure S52. Experimental (open circles) and simulated (filled lines) temporal concentrations of **C**-derived intermediates where model 3 is used in the generation of the simulated data.*

S6.1.3.2 Equimolar

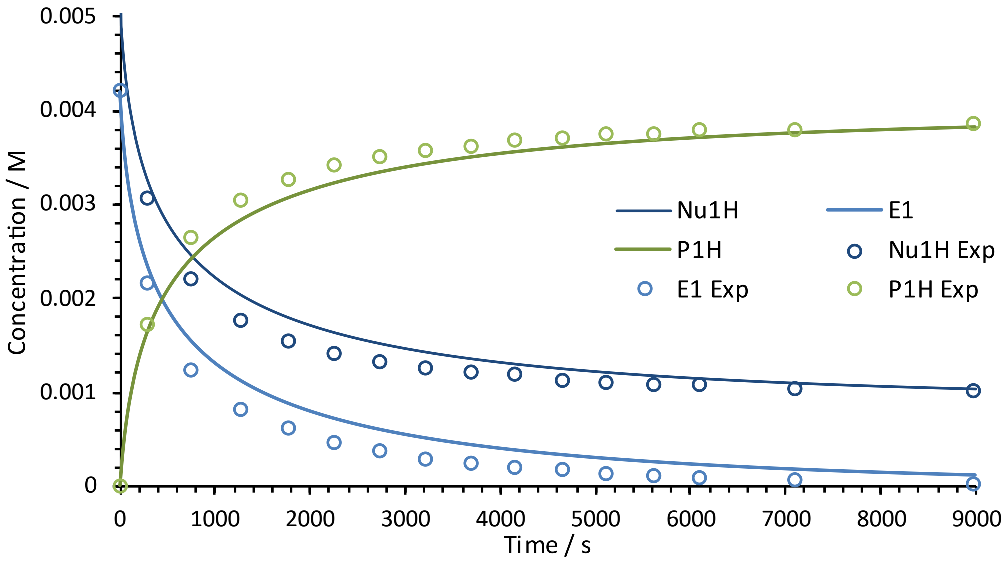

Figure S53. Experimental (open circles) and simulated (filled lines) temporal concentrations of limiting substrate and product where model 3 is used in the generation of the simulated data.

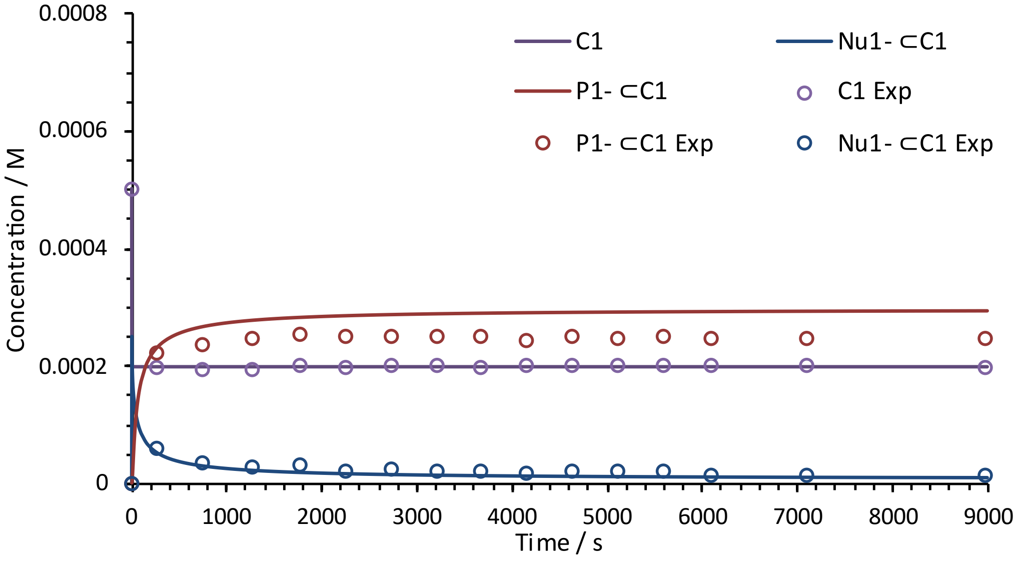

*Figure S54. Experimental (open circles) and simulated (filled lines) temporal concentrations of **C**-derived intermediates where model 3 is used in the generation of the simulated data.*

S6.1.4 Model 4

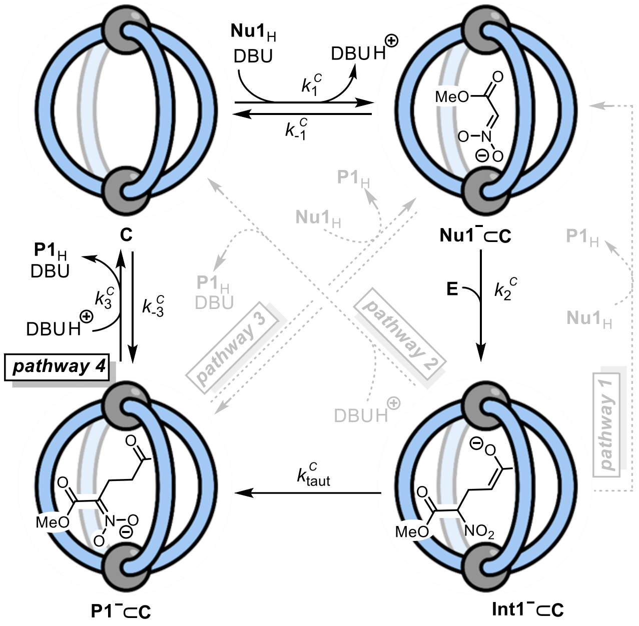

*Figure S55. Model 4 describing the DBU catalysed reaction between **Nu1**$_H$ and **E** in the presence of **C**.*

Table S7. Optimal kinetic and thermodynamic parameters fitted according to model 4.

Parameter / Units	Key thresholds/relationships
K_1^C / M^{-1}	$K_1^C > 10^4$
k_1^C / $M^{-2}s^{-1}$	$k_1^C > 10^8$
k_2^C / M^{-1} s^{-1}	$k_2^C = 27.4$
K_{-3}^C / M^{-1}	$K_1^C / K_{-3}^C = 0.131$
k_3^C / M^{-1} s^{-1}	$k_2^C / k_{-3}^C = 0.165$
k_{taut}^C / s^{-1}	$k_{taut}^C > 1$

Associative deprotonation:

$$C + DBU + Nu1_H \underset{k_{-1}^C}{\overset{k_1^C}{\rightleftharpoons}} Nu1^- \subset C$$

$$C + DBU + P1_H \underset{k_{-3}^C}{\overset{k_3^C}{\rightleftharpoons}} P1^- \subset C$$

Conjugate addition:

$$E + Nu2^- \subset C \xrightarrow{k_2^C} Int1^- \subset C$$

Tautomerisation:

$$Int1^- \subset C \xrightarrow{k_{taut}^C} P1^- \subset C$$

S6.1.4.1 $Nu1_H$ Excess

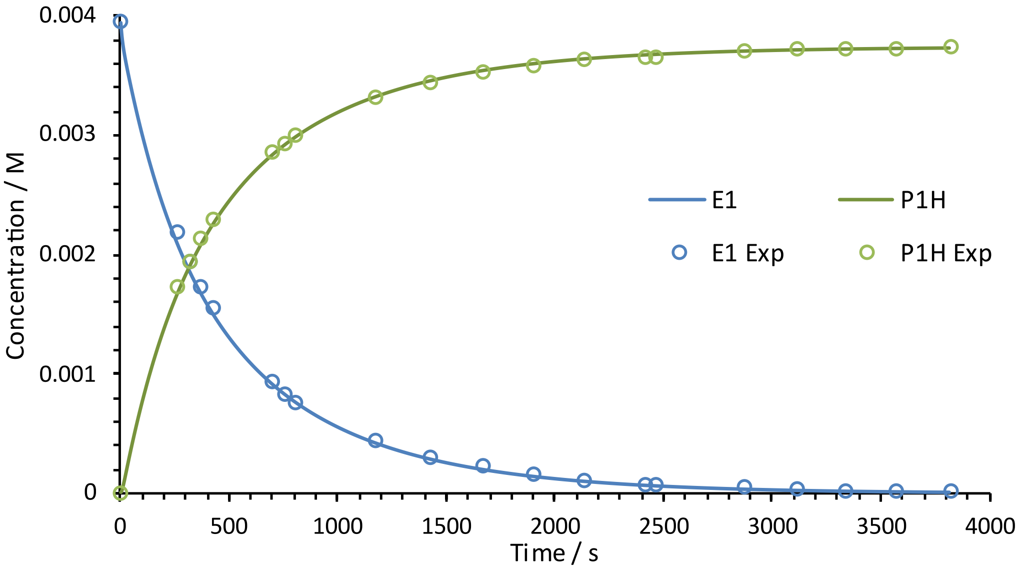

Figure S56. Experimental (open circles) and simulated (filled lines) temporal concentrations of limiting substrate and product where model 4 is used in the generation of the simulated data.

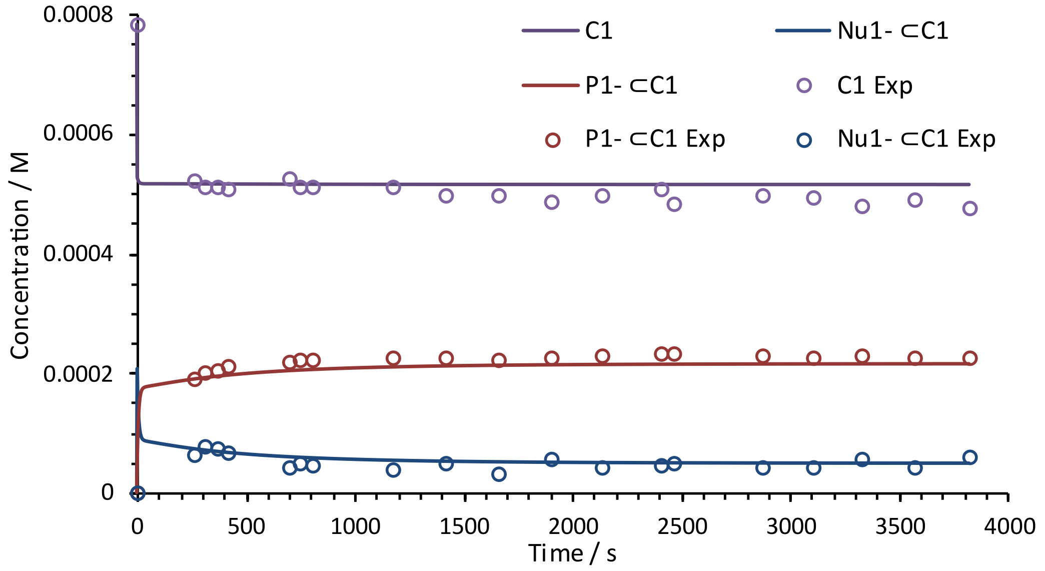

*Figure S57. Experimental (open circles) and simulated (filled lines) temporal concentrations of **C**-derived intermediates where model 4 is used in the generation of the simulated data.*

S6.1.4.2 Equimolar

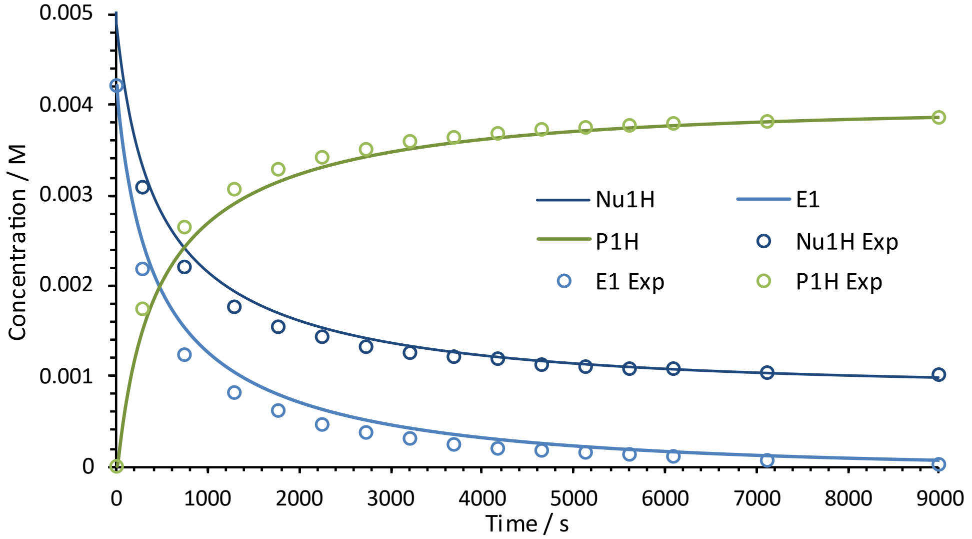

Figure S58. Experimental (open circles) and simulated (filled lines) temporal concentrations of limiting substrate and product where model 2 is used in the generation of the simulated data.

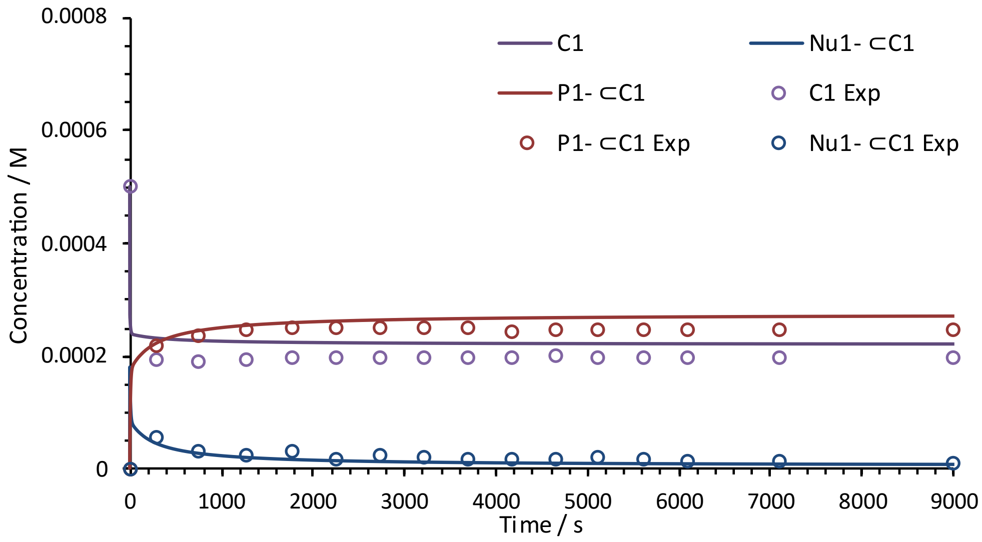

*Figure S59. Experimental (open circles) and simulated (filled lines) temporal concentrations of **C**-derived intermediates where model 4 is used in the generation of the simulated data.*

S6.2 Reaction 2 Model

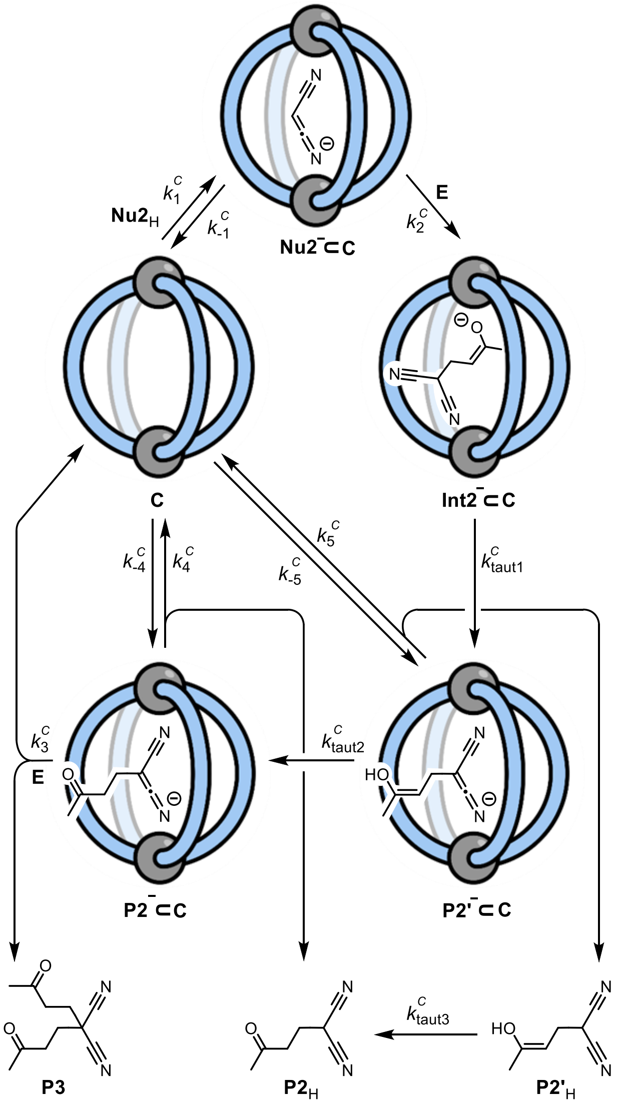

*Figure S60. Abridged model for the cage-mediated DBU catalysed reaction of **Nu2**$_H$ and **E**.*

Model

Associative deprotonation:

$$C + DBU + Nu2_H \underset{k_{-1}^C}{\overset{k_1^C}{\rightleftharpoons}} Nu2^- \subset C$$

$$C + DBU + P2_H \underset{k_4^C}{\overset{k_{-4}^C}{\rightleftharpoons}} P2^- \subset C$$

$$C + DBU + P2'_H \underset{k_5^C}{\overset{\mathrm{k}_{-5}^C}{\rightleftharpoons}} P2'^- \subset C$$

Conjugate addition:

$$E1 + Nu2^- \subset C \xrightarrow{k_2^C} Int2^- \subset C$$

$$E1 + P2^- \subset C \xrightarrow{k_3^C} P3 + C + DBU$$

Enol Isomerisation:

$$Int2^- \subset C \xrightarrow{k_{taut1}^C} P2'^- \subset C$$

$$P2'^- \subset C \xrightarrow{k_{taut2}^C} P2^- \subset C$$

$$P2'_H \xrightarrow{k_{taut3}^C} P2_H$$

Catalyst decomposition:

$$Nu2_H + Int2^- \subset C \xrightarrow{k_{dec}^C} X$$

Table S8. Optimal kinetic parameters obtained from fitting temporal concentrations from both datasets to model 4 mechanism outlined in Figure S60. Both the "E excess" and "Nu2$_H$ excess" datasets are fitted simultaneously. Temporal concentrations of P2$_H$, P3, P2'$_H$ and P3 are considered in both datasets, while only the limiting substrate is considered in either case.

Parameter	Units	Key thresholds/relationships
K_1^C	M^{-2}	$K_1^C > 10^6$
K_4^C	M^{-2}	$K_1^C/K_4^C = 43.3$
K_5^C	M^{-2}	$K_1^C/K_5^C = 26.9$
k_1^C	$M^{-2}s^{-1}$	$k_1^C > 10^9$
k_2^C	$M^{-1}s^{-1}$	$k_2^C = 3.33$
k_3^C	$M^{-1}s^{-1}$	$k_3^C/k_2^C = 1.82$
k_{-4}^C	$M^{-2}s^{-1}$	$k_1^C/k_{-4}^C = 2.69 \times 10^3$
k_{-5}^C	$M^{-2}s^{-1}$	$k_1^C/k_{-5}^C = 1.41 \times 10^3$
k_{taut}^C	s^{-1}	$k_{taut2}^C/k_{taut1}^C = 376$
k_{taut2}^C	s^{-1}	$k_{taut2}^C/k_{taut3}^C = 1.17 \times 10^4$
k_{taut3}^C	s^{-1}	
k_{Decomp}^C	$M^{-1}s^{-1}$	$k_{Decomp}^C/k_{taut1}^C = 1.37$

S6.2.1 E excess

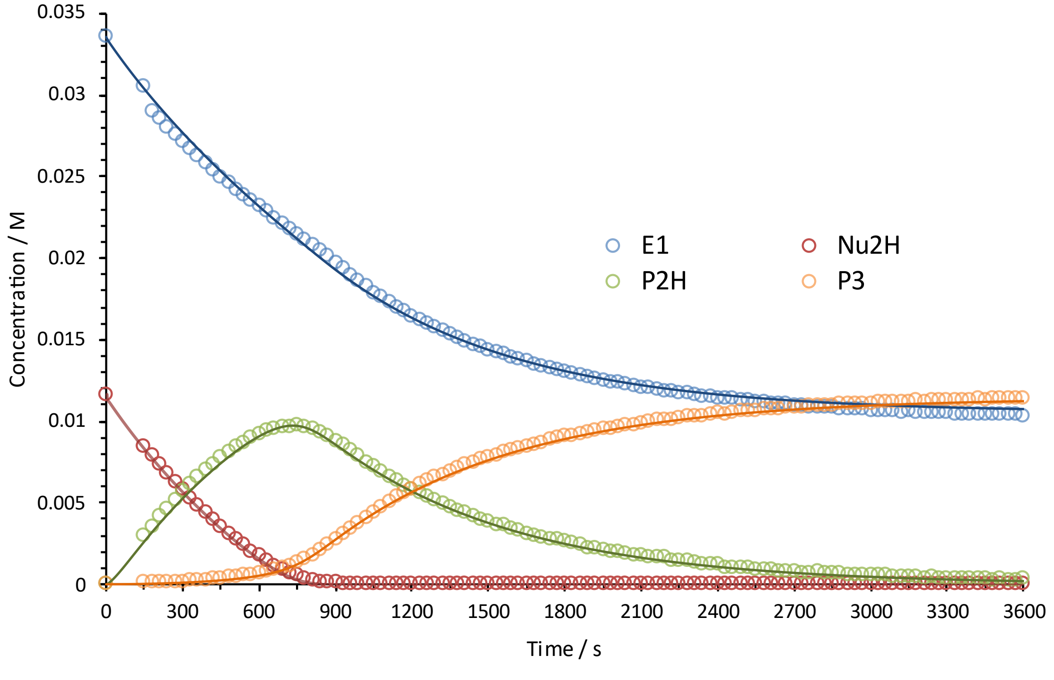

*Figure S61. Experimental (open circles) and simulated (filled lines) temporal concentrations of substrates **Nu2**$_H$, **E** and major products **P2**$_H$ and **P3** under **E** excess conditions.*

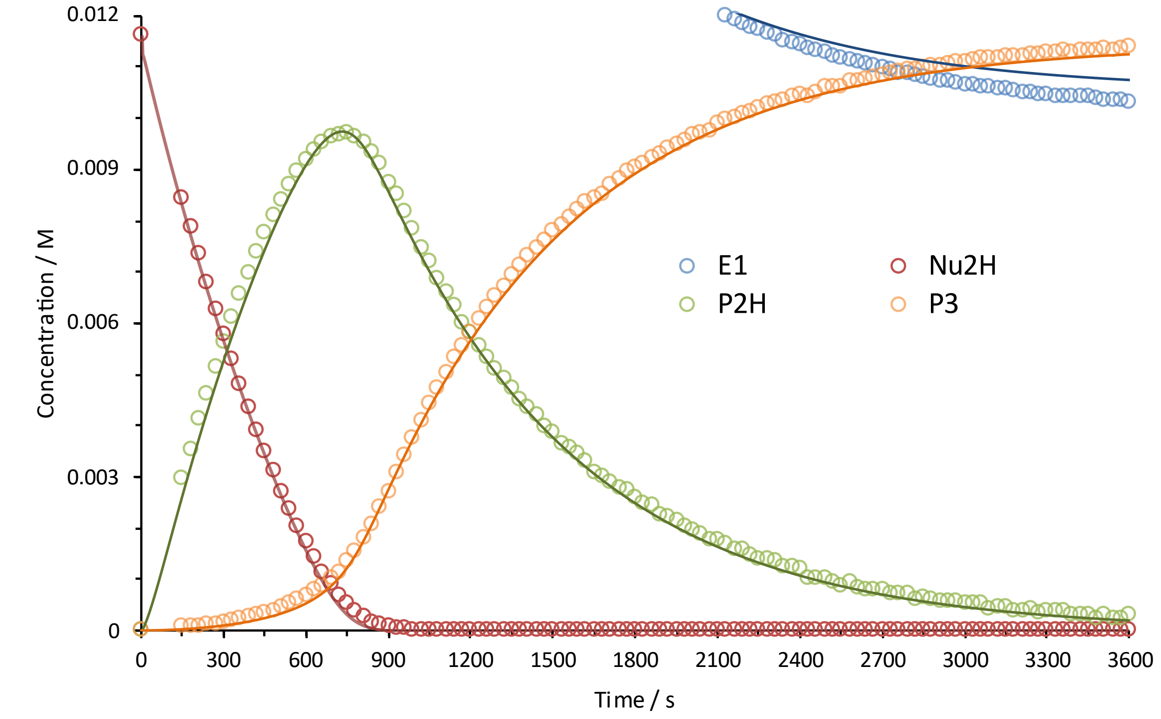

*Figure S62. Experimental (open circles) and simulated (filled lines) temporal concentrations of substrates **Nu2**$_H$, **E** and major products **P2**$_H$ and **P3** under **E** excess conditions.*

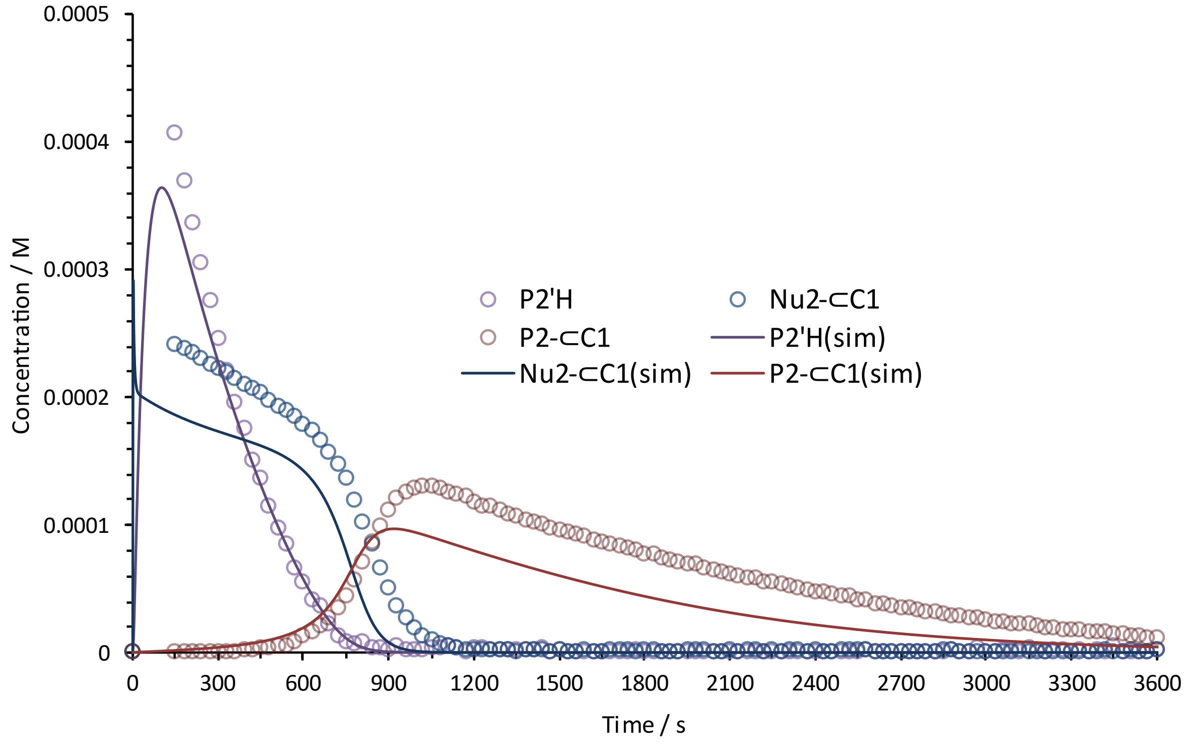

*Figure S63. Experimental (open circles) and simulated (filled lines) temporal concentrations of intermediates **P2'**$_H$, **Nu2**$^-$**⊂C** and **P2**$^-$**⊂C** under **E** excess conditions.*

S6.2.2 $Nu2_H$ Excess

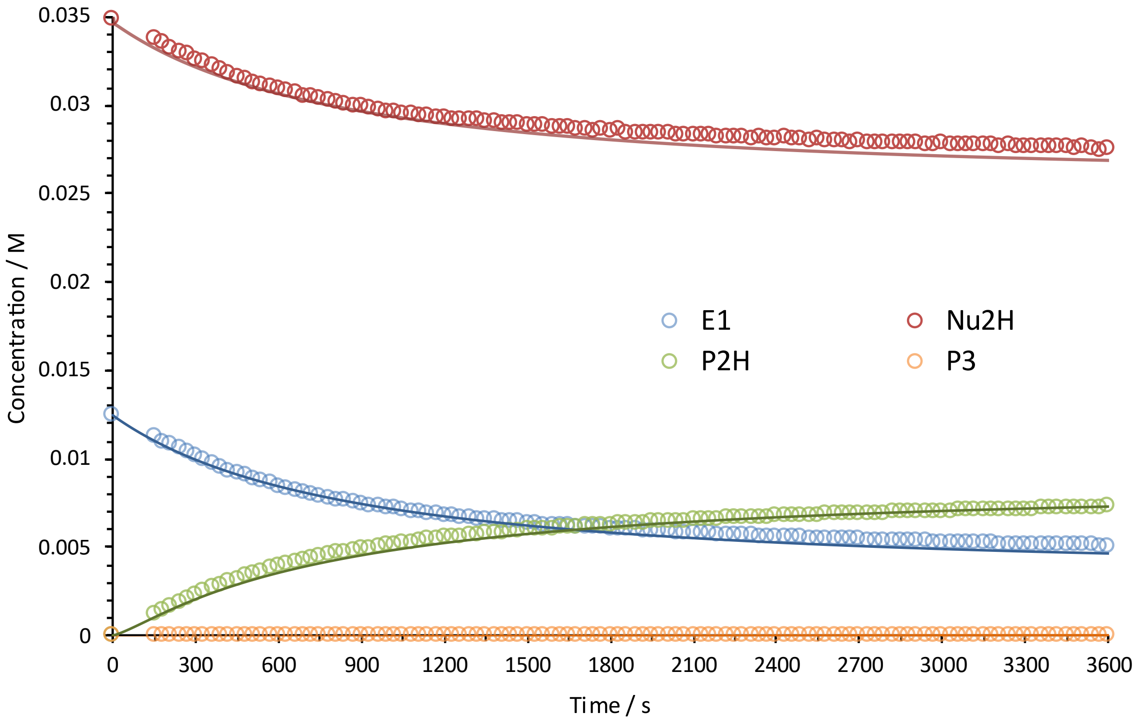

Figure S64. Experimental (open circles) and simulated (filled lines) temporal concentrations of substrates ***Nu2**$_H$*, **E*** *and major products* ***P2**$_H$* *and* ***P3*** *under* ***Nu2**$_H$* *excess conditions.*

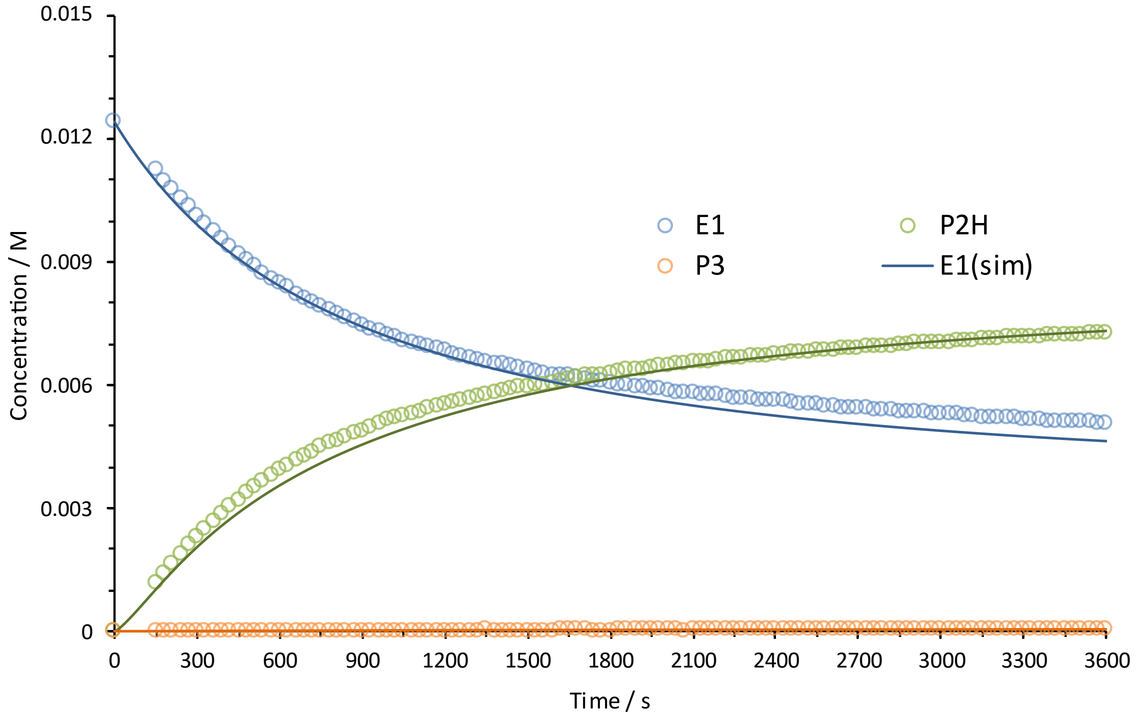

Figure S65. Experimental (open circles) and simulated (filled lines) temporal concentrations of substrate ***E*** *and major products* ***P2**$_H$* *and* ***P3*** *under* ***Nu2**$_H$* *excess conditions.*

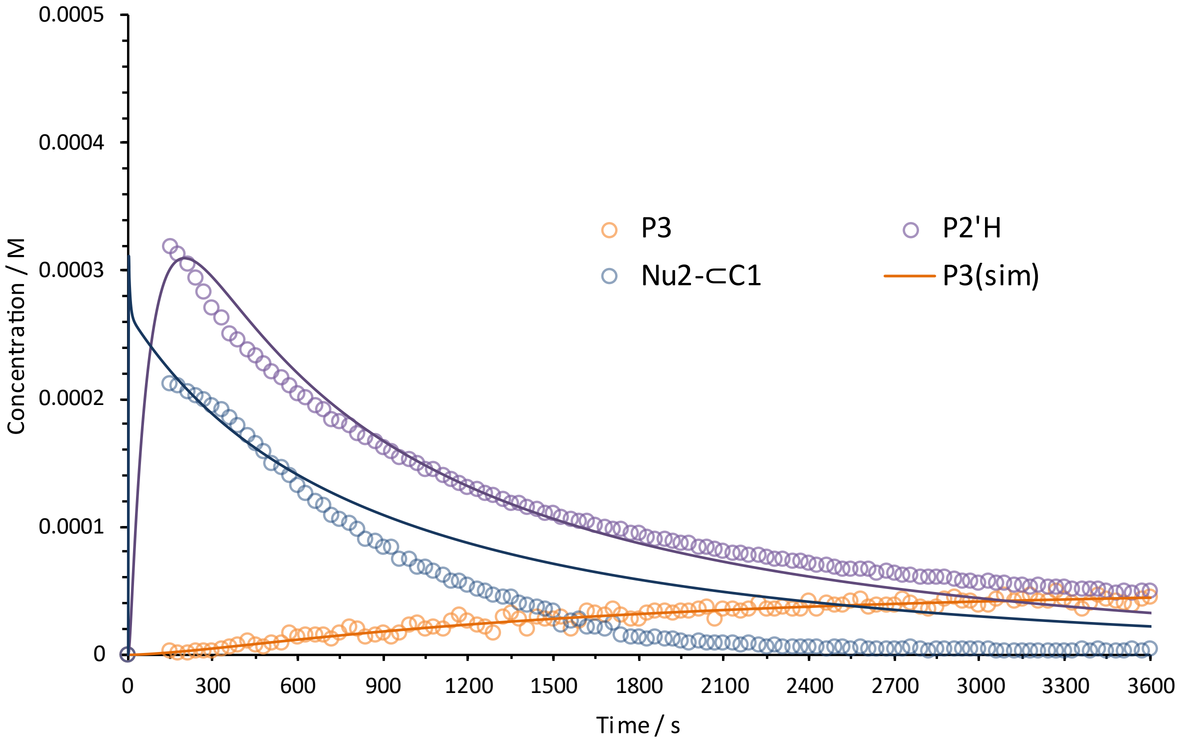

Figure S66. Experimental (open circles) and simulated (filled lines) temporal concentrations of intermediates ***P2'$_H$*** *and* ***Nu2$^-$⊂C*** *and product* ***P3*** *under* ***Nu2$_H$*** *excess conditions.*

S7. NMR titrations

NMR titrations were conducted using a titration NMR tube with a twist-top vial cap using a starting volume of 500 µL. In all cases, a solution of DBU (2 mM) and pronucleophile **NuX**$_{\mathrm{H}}$ (>10 mM, known excess) was titrated into a solution of DBU of the same concentration using a Hamilton syringe. After each addition, the sample was shaken to ensure complete mixing, and a ^{1}H spectrum was acquired. The change in chemical shift of a characteristic resonance was recorded and fitted to a 1:1 fast exchange binding model in Microsoft Excel using the solver add-in, according to the equations

$$H + G \rightleftharpoons HG$$

$$K = \frac{[HG]_i}{[H]_i[G]_i}$$

$$[G]_{T,i} = [G]_i + [HG]_i$$

$$[H]_0 = [H]_i + [HG]_i$$

$$\frac{\delta_0 - \delta_i}{\delta_0 - \delta_{max}} = \frac{[HG]_i}{[H]_0} = \frac{1}{2[H]_0}\left([H]_0 + [G]_{T,i} + \frac{1}{K}\right) - \frac{1}{2[H]_0}\sqrt{\left(-[H]_0 - [G]_{T,i} - \frac{1}{K}\right)^2 - 4[H]_0[G]_{T,i}}$$

Where δ_i is the chemical shift of the resonance of interest in the ith spectrum, δ_0 is the chemical shift of this resonance prior to the first addition, and δ_{max} is the chemical shift of this resonance at saturation. $[H]_0$ is the total concentration of DBU in the sample, which remains constant throughout the titration.

$[G]_{T,i}$ is the *total* concentration of pronucleophile **NuX**$_{\mathrm{H}}$ present in the ith spectrum, and is determined using

$$[G]_{T,i} = \left(\frac{\sum_{n=1}^{i} V_n}{V_0 + \sum_{n=1}^{i} V_n}\right)[G]_{max}$$

Where V_0 is the initial volume of the sample prior to addition of **NuX**$_{\mathrm{H}}$ (500 µL) and $\sum_{n=1}^{i} V_n$ denotes the total volume of the of titrant added to the sample directly before each acquisition, *up to and including* the ith spectrum. $[G]_{max}$ is the concentration of **NuX**$_{\mathrm{H}}$ in the titrant solution (alongside DBU).

S7.1 Titration of $Nu1_H$ with DBU in the absence of C

A solution of **Nu1**$_H$ (16.7 mM) and DBU (2 mM) was titrated into a 500 µL solution of DBU (2 mM) in 2.5, 5, or 10 µL aliquots.

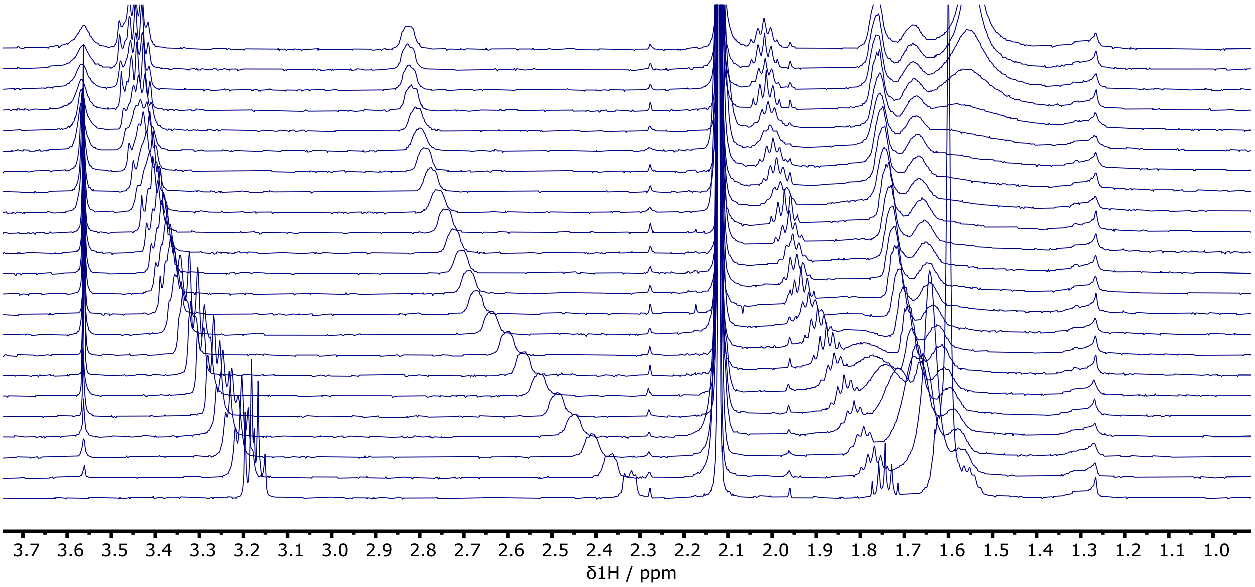

*Figure S67. Partial ^{1}H NMR spectra showing the change in resonances throughout the ^{1}H NMR titration of **Nu1**$_H$ and DBU. The DBU resonance used for model fitting begins at 2.325 ppm and approaches 2.820 ppm as **Nu1**$_H$ is added.*

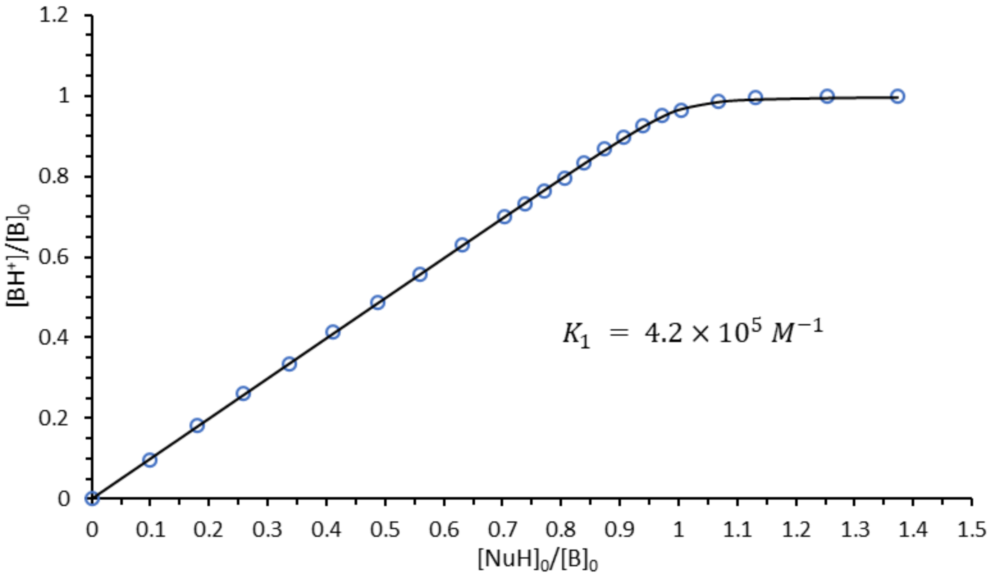

*Figure S68. Experimental (open circles) and fitted (filled line) change in DBU speciation as **Nu1**$_H$ is added, with the fitted value of the 1:1 equilibrium constant shown.*

S7.2 Titrations of Nu2$_H$ with DBU in the absence of C

A solution of **Nu2**$_H$ (21.6 mM) and DBU (2 mM) was titrated into a 500 µL solution of DBU (2 mM) in 2.5, 5, or 10 µL aliquots, with ^{1}H spectra measured after each addition.

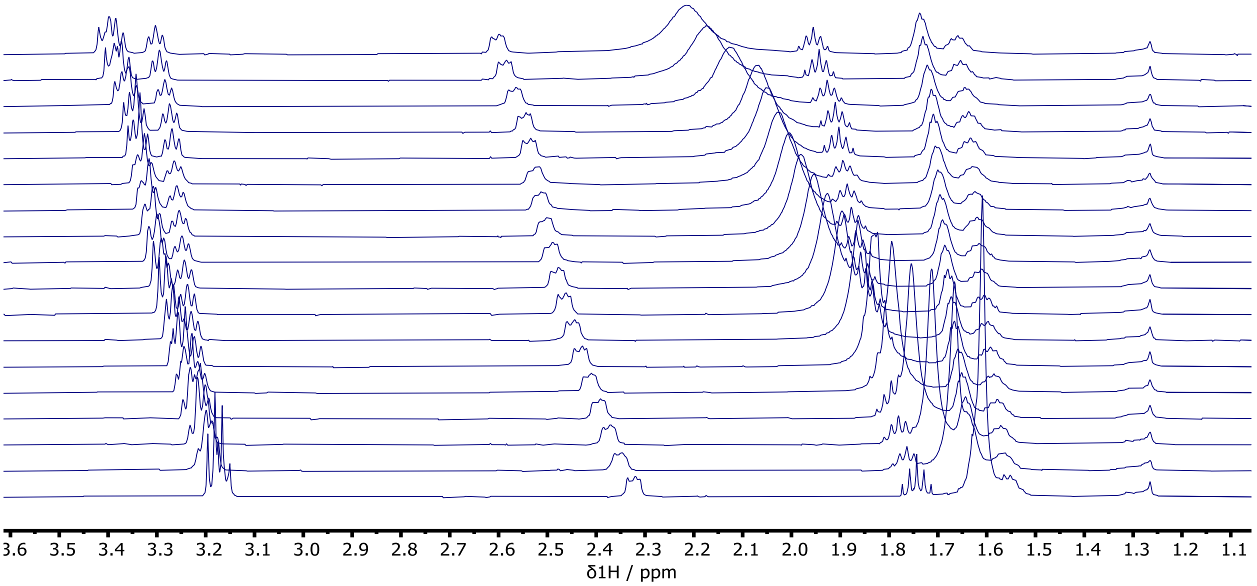

*Figure S69. Partial ^{1}H NMR spectra showing the change in resonances throughout the ^{1}H NMR titration of **Nu2**$_H$ and DBU. The DBU resonance used for model fitting begins at 2.325 ppm and approaches 2.750 ppm as **Nu1**$_H$ is added.*

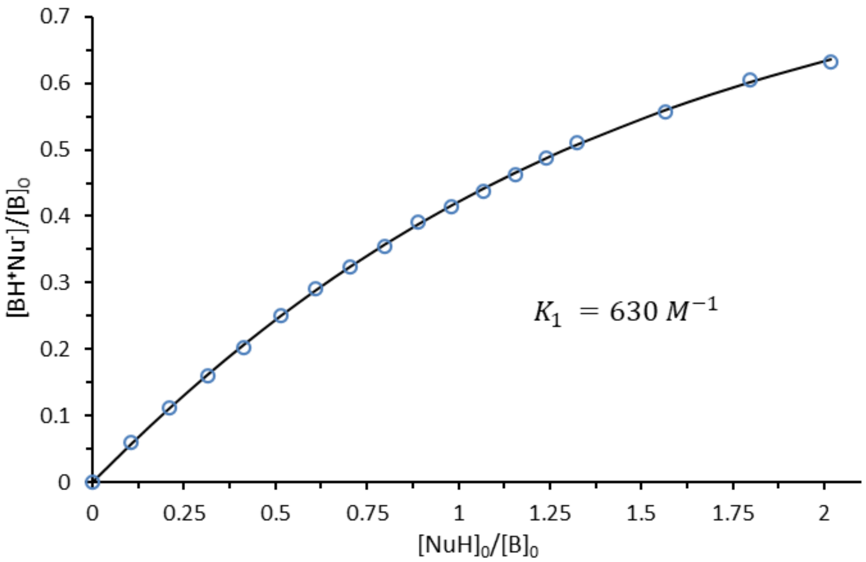

*Figure S70. Experimental (open circles) and fitted (filled line) change in DBU speciation as **Nu2**$_H$ is added, with the fitted value of the 1:1 equilibrium constant shown.*

S7.3 Titrations of $Nu1_H$ with DBU in the presence of C

S7.3.1 Competitive titration of DBU in the presence of Anthraquinone (AQ) and excess $Nu1_H$

Since the kinetic models suggest K_1^C values greater than $10^4\ M^{-1,}$ it was anticipated that measurement of this value by direct titration would be challenging due to saturation behaviour at NMR concentrations. To circumvent this, a competitive titration was undertaken in which the strong neutral guest (anthraquinone, **AQ**; $K_a = 5 \times 10^7\ M^{-1}$) would be displaced by **Nu1**$^-$, generated *in situ* by titration of a solution DBU into a solution containing pre-equilibrated **$Nu1_H$**, **C** and **AQ**. It is important to consider that while K_a and K_1^C have the same units (M^{-1}), the structures of the processes are fundamentally different: K_a describes a direct association while K_1^C describes an associative deprotonation (see section S6.1).

The titration was conducted by adding a concentrated solution of DBU in CD_2Cl_2 in 2 µL portions to a 500 µL CD_2Cl_2 solution of **C** (0.73 mM), Anthraquinone (**AQ**, 1.20 mM) and **$Nu1_H$** (>100-fold excess). A stacked set of partial NMR spectra is shown in Figure S71, and the corresponding concentration-concentration plot shown in figure S72. In the eighth spectrum (reading from bottom to top), the total concentration of DBU becomes equimolar with **C**. After this point free ligand resonances (8.56 ppm and 8.80 ppm) become well-resolved and increase linearly with addition DBU.

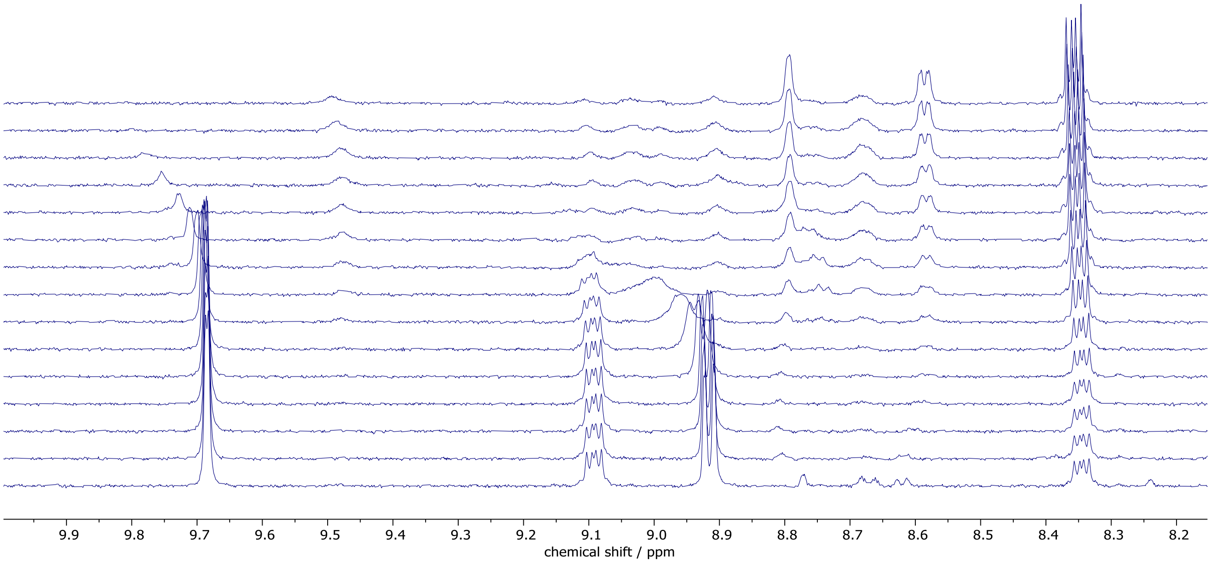

*Figure S71. Partial ^{1}H NMR Spectra (400 MHz) of the **C** and **AQ** resonances during a titration of DBU solution into a solution of **C** (0.73 mM) and AQ (1.2 mM) in the presence of excess **$Nu1_H$**. The resonance at 9.68 ppm corresponds to the cage interior ortho-pyridyl hydrogen atoms of the **AQ**⊂**C** complex. The resonance beginning at 8.91 ppm corresponds to the exterior ortho-pyridyl hydrogen atoms of the **AQ**⊂**C** complex. The resonances ending at 8.8 ppm and 8.56 ppm correspond to un complexed ligand resulting from decomposition of **C**. The resonance at 8.34 ppm corresponds to un-complexed **AQ**.*

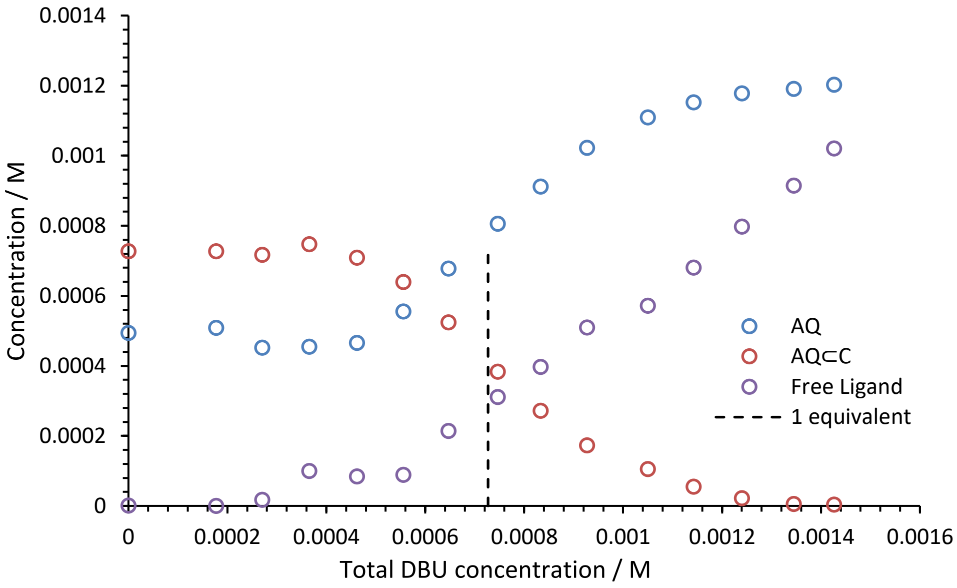

*Figure S72. Experimentally determined equilibrium concentrations of **C**, **AQ**, **AQ⊂C** and free ligand (open circles) as a function of total **DBU** concentration. Values are obtained by integrating and normalising the resonances shown in Figure S71.*

The speciation shown in Figure S72 highlights several points. The lack of response after the first four additions of DBU solution suggests that **Nu1**$^-$ is initially unable to outcompete the bound AQ. As more DBU is added, the **AQ** complex is depleted and unbound **AQ** is released into solution, with complete release apparent at ~2 equivalents of DBU with respect to **C**. However, it is interesting to note that despite the release of **AQ** from the complex, resonances corresponding to **Nu1**$^-$**⊂C** are not detectable in this titration (See section S5). Also of note is that after the addition of one equivalent of DBU with respect to **C**, a growing quantity of free ligand (constituent component of **C**) becomes detectable in solution. This suggests that **AQ** release during this titration is a result of decomposition rather than guest exchange.

S7.3.2 Direct titration of DBU into C in the presence of excess $Nu1_H$

To further examine the binding affinity of **Nu1**$^-$, a direct titration was conducted (without the presence of a competitor). This titration was conducted by adding a concentrated solution of DBU in CD_2Cl_2 in 2 µL portions to a 500 µL CD_2Cl_2 solution of **C** (0.57 mM) and $\mathbf{Nu1_H}$ (>100-fold excess).

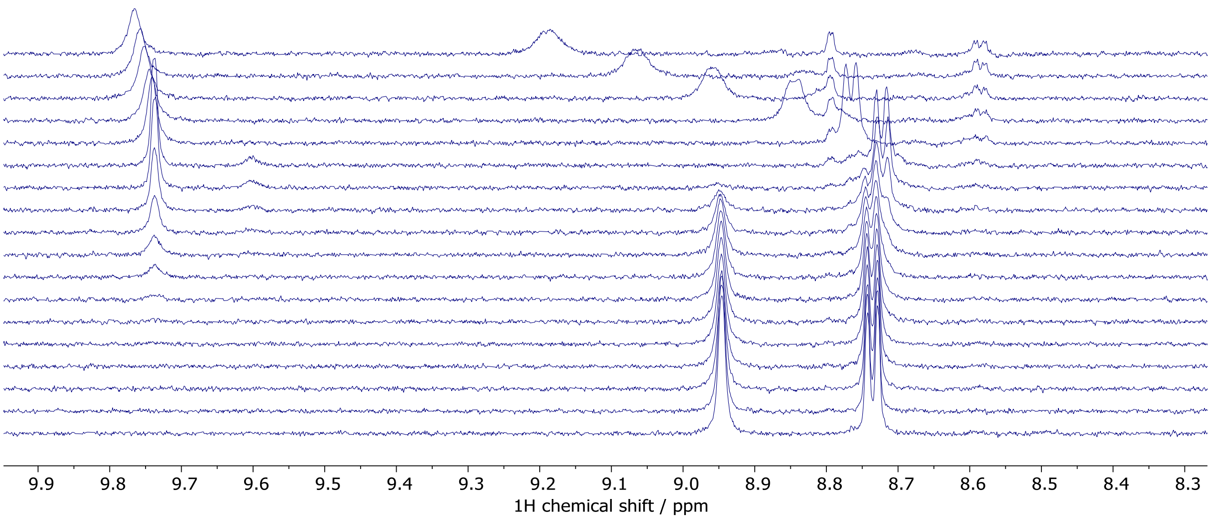

*Figure S73. Partial ^{1}H NMR Spectra (400 MHz) of the **C**, **Nu1**$^-$⊂**C**, and free ligand resonances during a titration of DBU solution into a solution of **C** (0.57 mM) and in the presence of excess $\mathbf{Nu1_H}$. The resonance at 9.75 ppm corresponds to the cage interior ortho-pyridyl hydrogen atoms of the **Nu1**$^-$⊂**C** complex. The resonance beginning at 8.74 ppm corresponds to the exterior ortho-pyridyl hydrogen atoms of **C**. The resonances ending at 8.8 ppm and 8.56 ppm correspond to un complexed ligand resulting from decomposition of **C**.*

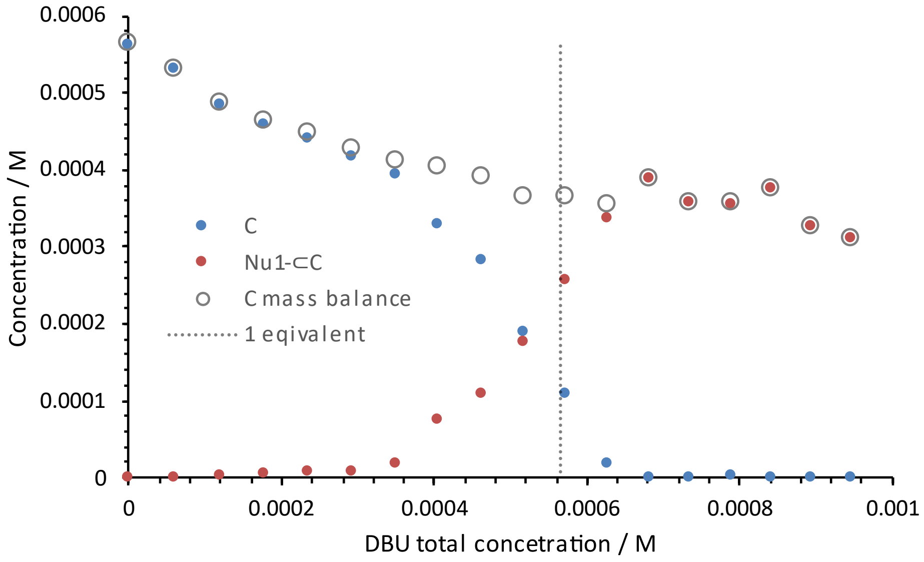

*Figure S74. Experimentally determined equilibrium concentrations of **C**, free ligand (filled circles) and C mass balance (open circles, sum of red and blue filled circles) as a function of total **DBU** concentration. Values are obtained by integrating and normalising the resonances shown in Figure S71.*

The speciation shown in Figure S74 exhibits some of the same characteristics present in the competitive titration. There is initial period in which addition of DBU does not appear to result in detectable **Nu1**$^-$**⊂C** (note the loss in C mass balance during this initial period). This could be the result of line broadening due to exchange rate since the first detection of **Nu1**$^-$**⊂C** resonance at 9.74 ppm (from spectrum 6 in Figure S73, reading from bottom to top) is broad and appears to sharpen as more DBU is added. Another feature of note in this titration is that from spectrum 11, (after which DBU becomes super-stoichiometric with respect to **C**) the resonance corresponding to the exterior ortho-pyridyl hydrogen atoms begins to move with each further addition of DBU, indicating the formation of complexes of the type **Nu1**$^-$**⊂C···Nu1**$^-$. This indicates, alongside further deterioration in the **C** mass balance and the appearance in the spectra of free ligand resonances that generation of excess **Nu1**$^-$.DBU$_H^+$ promotes capsule decomposition.

This titration, as well as the competitive titration in section S7.3.1, illustrate that due to decomposition of **C** when exposed to excess **Nu1**$^-$.DBU$_H^+$, it is very challenging to design an experiment which could be used to accurately determine K_1^C in isolation. It is important to note that when DBU is present in sub-stoichiometric quantities, no cage decomposition is observed, hence there is no adverse effect on the **Nu1**$_H$ + **E** reaction system or the kinetic modelling thereof, presented in section S6.1.

S8. DBU catalysed Michael addition in the absence of C

The DBU-catalysed Michael addition reaction of **Nu1**$_H$ and **E** in the absence of **C** was monitored *via* ^{1}H NMR according to the standard procedure outlined in section 4.1, with the provision that no **C** was added to the NMR tube prior to solvent. Due to the slow rate of reaction, the concentrations of the substrates and the catalyst were increased from conditions previously described.

S8.1 Reaction 1

*Table S9. Initial concentrations for the conditions under which reaction 1 was studied in the absence of **C**.*

Conditions	$[E]_0$ / mM	$[Nu1_H]_0$ / mM	$[C]_0$ / mM	$[DBU]_0$ / mM
"High base"	21.0	82.6	0	2.48
"Medium base"	21.1	82.6	0	1.63
"Low base"	21.6	81.3	0	0.80

S8.1.1 Spectra

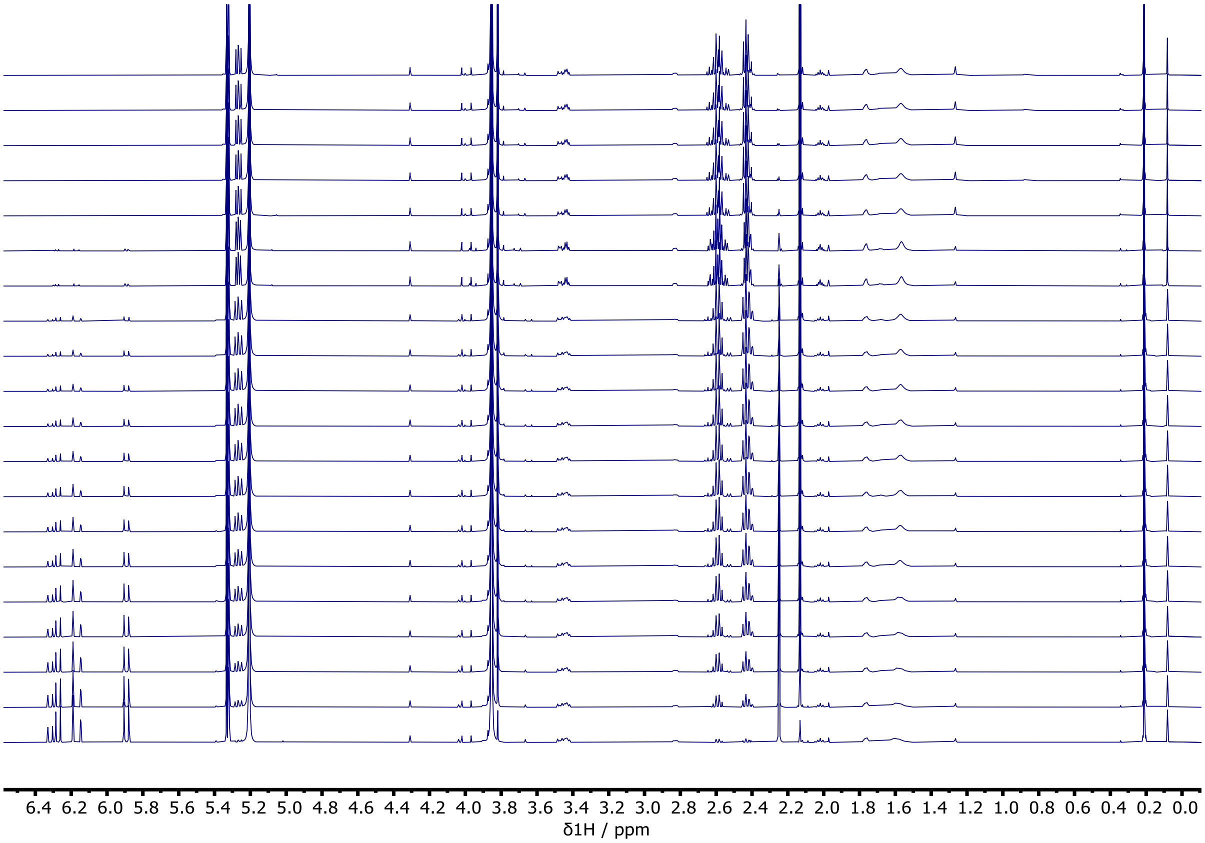

*Figure S75. Temporal ^{1}H NMR Spectra (600 MHz) of the DBU catalysed reaction of **Nu1**$_H$ and **E** under high base loading conditions.*

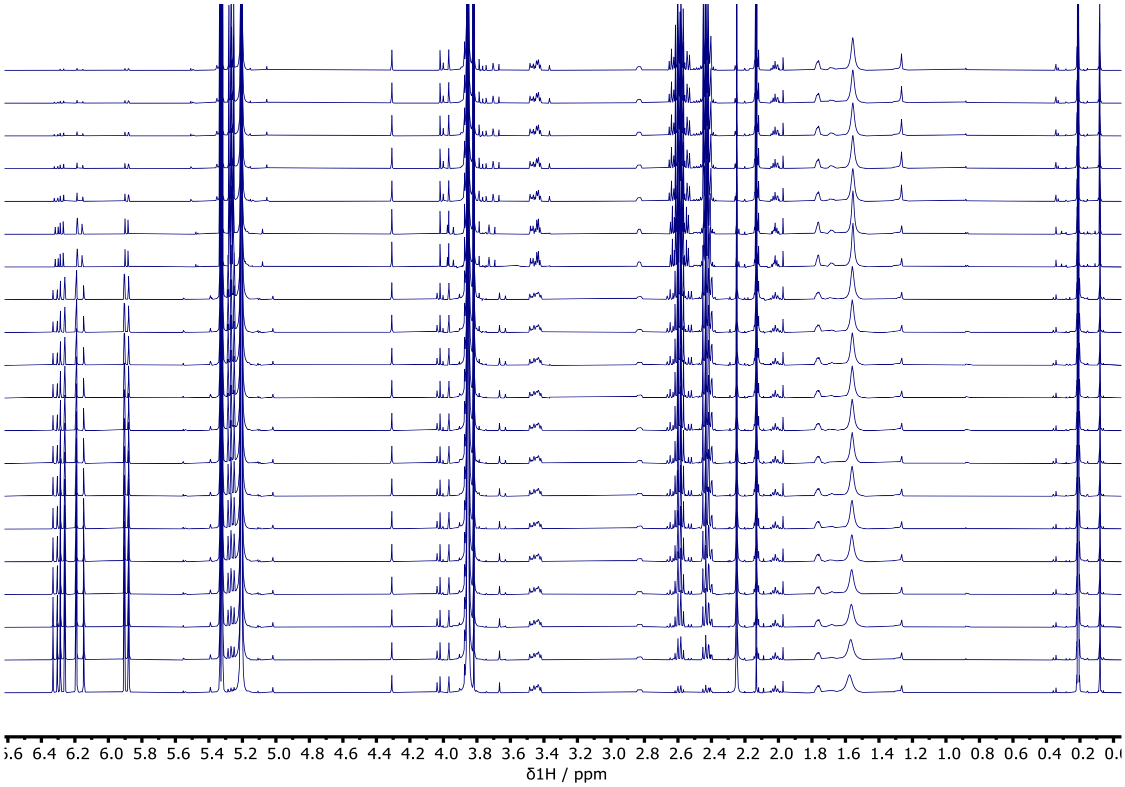

*Figure S76. Temporal ^{1}H NMR Spectra (600 MHz) of the DBU catalysed reaction of **Nu1**$_H$ and **E** under medium base loading conditions.*

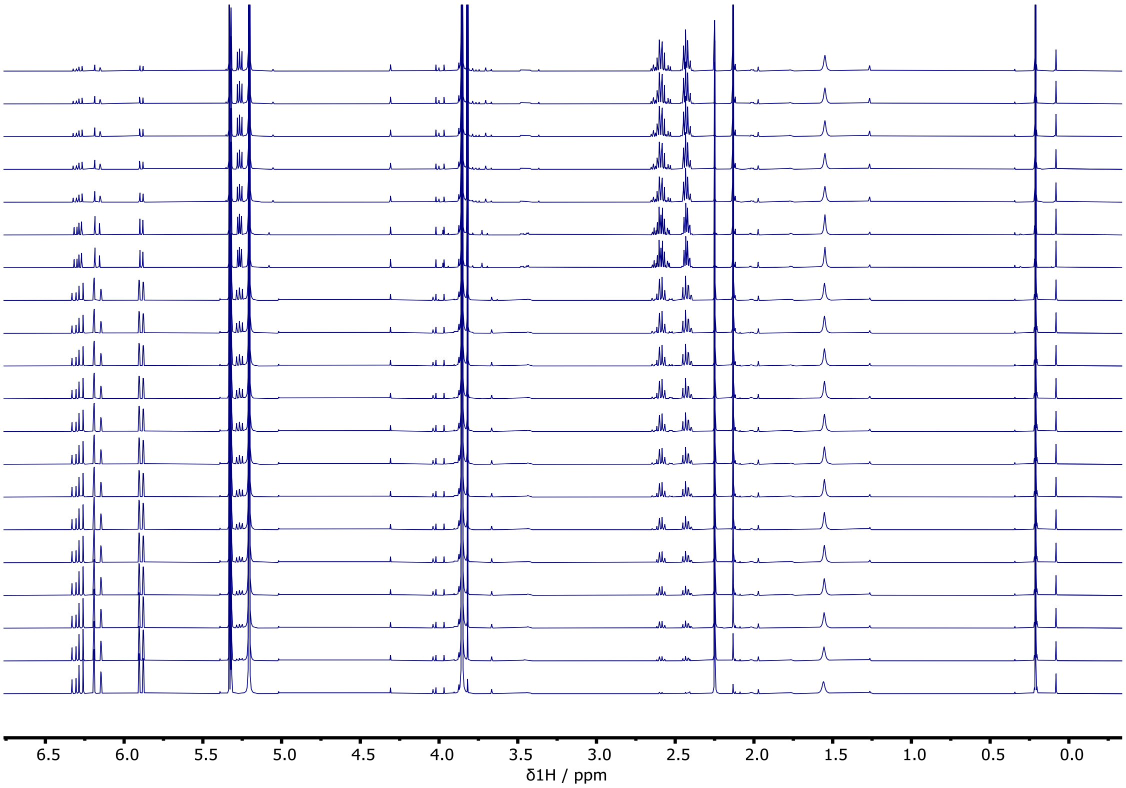

*Figure S77. Temporal ^{1}H NMR Spectra (600 MHz) of the DBU catalysed reaction of **Nu1**$_H$ and **E** under low base loading conditions.*

S8.1.2 Reaction 1 Model

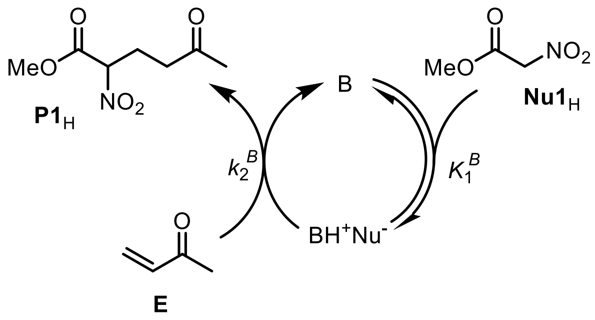

*Figure S78. Schematic representation of the base-catalysed Michael addition reaction between **Nu1**$_H$ and **E**.*

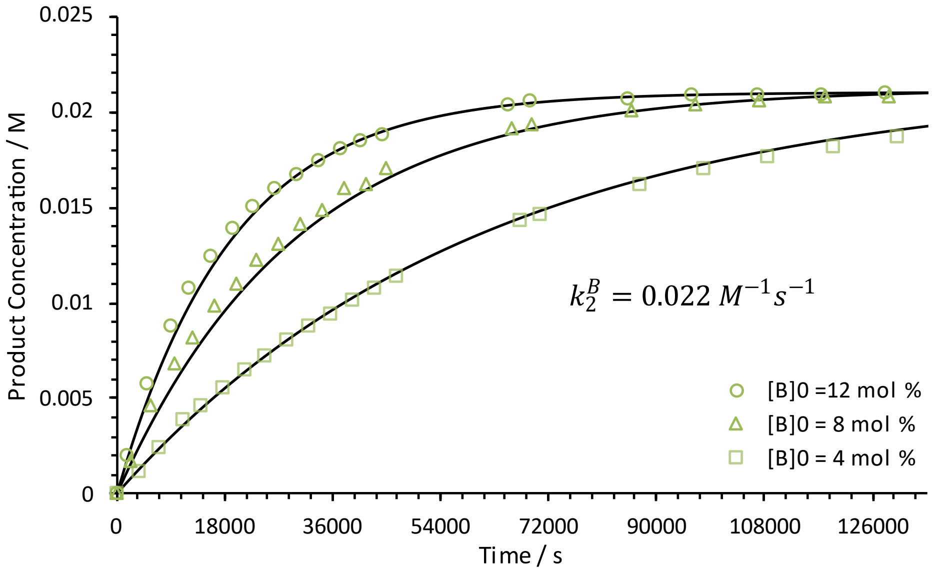

*Figure S79. Experimental (open shapes) and simulated (filled lines) temporal concentrations of **P1**$_H$, in the DBU catalysed reaction of **Nu1**$_H$ and **E** in the absence of **C**, under all conditions.*

*Table S10. Optimal kinetic and thermodynamic parameters fitted according to reaction 1 model in the absence of **C**. [a] value obtained from fitting titration data in section S7.1. [b] value assumed to ensure k_2 is rate-limiting. The value of k_2^B reflects an average of the value obtained in each of the three runs; the maximum deviation in k_2^B between any pair of runs is ~4%.*

Parameter / Units	Fitted value
K_1^B / $\mathrm{M^{-1}}$	$^{a}4.20 \times 10^5$
k_1^B / $\mathrm{M^{-1}s^{-1}}$	$^{b}10^6$
k_2^B / $\mathrm{M^{-1}s^{-1}}$	2.16×10^{-2}

S8.2 Reaction 2

*Table S11. Initial concentrations for the conditions under which reaction 2 was studied in the absence of **C**.*

Conditions	$[E]_0$ / mM	$[Nu1_H]_0$ / mM	$[C]_0$ / mM	$[DBU]_0$ / mM
"**E** Excess"	35.0	11.9	0	0.315
"**Nu2**$_H$ Excess"	9.98	29.5	0	0.338

S8.2.1 Spectra

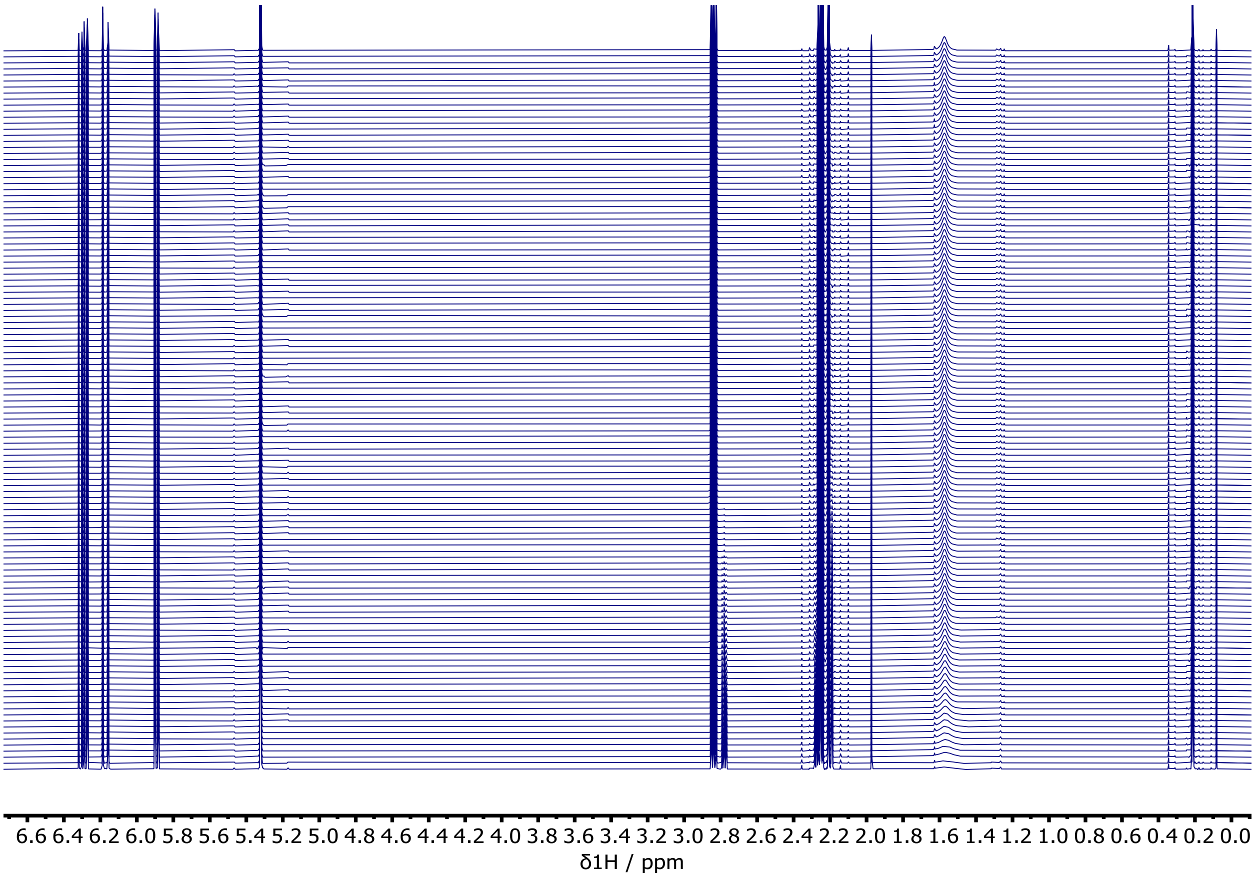

*Figure S80. Temporal ^{1}H NMR Spectra (600 MHz) of the DBU catalysed reaction of **Nu2**$_H$ and **E** under **E** excess conditions.*

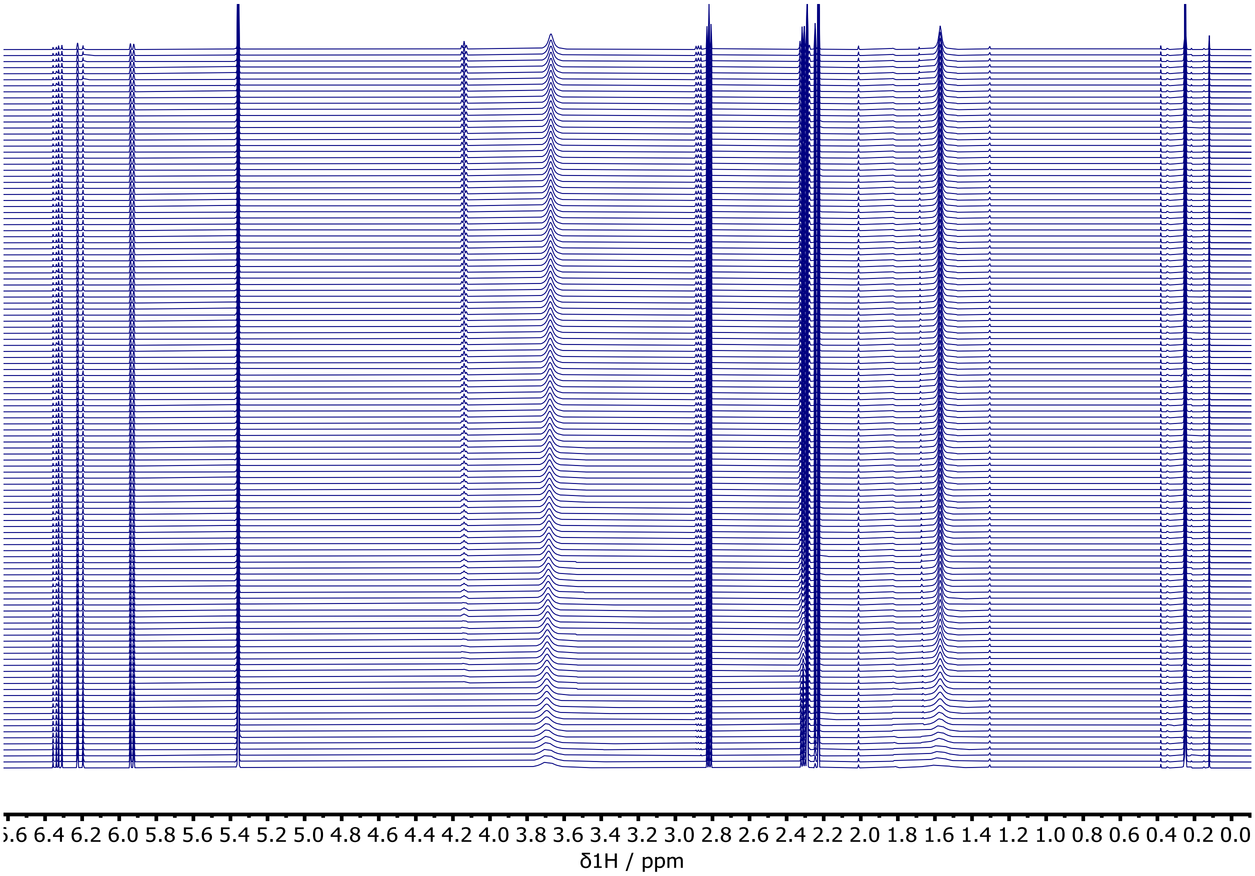

*Figure S81. Temporal ^{1}H NMR Spectra (600 MHz) of the DBU catalysed reaction of **Nu2**$_H$ and **E** under **Nu2**$_H$ excess conditions.*

S8.2.2 Discussion

Using the same strategy as for **Nu1**$_H$, we compared the kinetics of the cage-mediated base-catalysed to the kinetics of a reaction where no **C** is added. One of our immediate interests for this substrate pair was the cage's impact on the acidity of the pronucleophile substrate, since **Nu2**$_H$ is has a significantly lower aqueous pK_a than **Nu1**$_H$ ($\Delta pK_a > 5$). Using ^{1}H NMR titration, we extracted an association constant in CD_2Cl_2 for DBU and **Nu2**$_H$, finding that it amounts to $ca.\, 630\ M^{-1}$ (Figure S70).

We then sought to employ the same method as the background reaction with **Nu1**$_H$ to determine the extent of rate enhancement of the addition step(s). However, when monitoring this background reaction, it immediately became apparent that the DBU-only reaction shows unusual kinetic profiles, where the rate of reaction appeared to decrease significantly at high conversions. To rationalise these apparently inhibitory kinetics, we envisaged a scenario in which the first Michael addition product **P2**$_H$ might dominate substrate interactions with the catalyst, effectively inhibiting its own formation and that of **P3**. Such behaviour might be the result of the pendent ketone present in **P2**$_H$ promoting enhanced association with DBUH$^+$ in dichloromethane *via* chelation (Figure S78). To assess the potential for this product inhibition, a titration of **P2**$_H$ against DBU was attempted. Unfortunately, results were inconclusive due to decomposition, but did not suggest that there was a notable difference in the affinity for DBU between **Nu2**$_H$ and **P2**$_H$. Further, kinetic fits using this mechanism (Figure S78) failed to account for the kinetics of two different initial conditions. Additionally, this proposal would require a large difference in acidity between **Nu2**$_H$ and **P2**$_H$, something that is difficult to reconcile with their structural similarity.

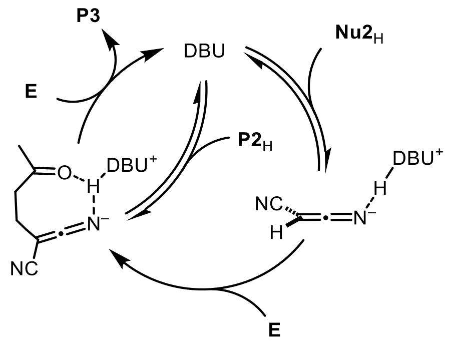

*Figure S82. Initial proposal for the mechanism of the reaction of **Nu2**$_H$ and **E** catalysed by DBU in the absence of **C**.*

Knowing that the rate reduction was unlikely to be caused by inhibition from **P2**$_H$, we considered other hypotheses. A second possibility for the rapidly slowing reaction was that, rather than being inhibited, the reaction is of high total order, greater than one in at least one component. A regular Michael addition reaction might be expected to be second order overall (first order in both substrates), but a reaction with an even higher total order would display an even more rapid rate reduction as the reaction progresses, similar to that of a product-inhibited reaction. With this revised outlook, we quickly found that the reaction was much better described by a mechanism second order in enone **E**, implying that one cycle of the catalyst system utilises two equivalents of **E**. To rationalise this peculiar result, we considered the

strong ambident nucleophilicity of **Nu2⁻**, and hypothesised that it could be uncovering the 1,2-addition reactivity of the enone electrophile **E** (Figure S79, step II), rather than the expected 1,4-addition reactivity characteristic of Michael addition reactions. We envisaged a mechanism in which the carbonyl of **E** is able to trap the activated nucleophile at its N-terminus, leading to a carbinol-ketenimine alkoxide. Such an intermediate might coordinate more strongly with the conjugate acid of DBU due to its increased H-bond donor capability. This intermediate (composed of DBU, **E** and **Nu2**$_H$) could then act as a masked **Nu2⁻**, delivering the reactive carbon centre on its ketenimine subunit *via* an addition-elimination step (Figure S79, step III). This addition to a second equivalent of **E** would be the anticipated 1,4-addition step, forming the product enolate with the correct C-C connectivity and releasing the first equivalent of **E** back into solution. It is also noteworthy that in contrast to the C-catalysed variant, no enol **P2'**$_H$ was detected during this reaction.

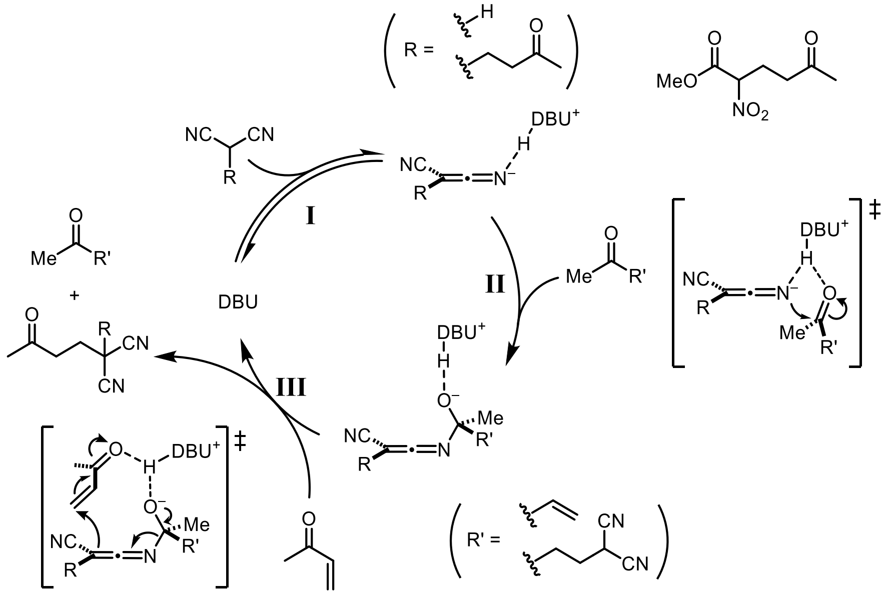

*Figure S83. Revised proposal for the mechanism of the reaction of **Nu2**$_H$ and **E** catalysed by DBU in the absence of **C**.*

While this reactivity pathway accounts for the initial second order dependence on **E**, it alone doesn't give a good fit for the full kinetic profile of both initial conditions when fit together. However, the fit can be greatly improved by considering the general case of this chemistry, where any of the carbonyls present in the solution might act as a "carrier" for the nucleophile *via* 1,2-addition. In fact, the net product of the first Michael addition reaction, **P2**$_H$, would likely be a better acceptor according to this reasoning, as it bears a carbonyl whose 1,2-reactivity isn't reduced by conjugation, as in the case of the enone substrate **E**. In practice, considering only **P2**$_H$ as an additional 1,2-acceptor gives a good fit to the experimental data (Figure S79 and Figure S80).

Deprotonation:

$$DBU + Nu2_H \underset{k_{-1}^{C}}{\overset{\mathrm{k}_1^{C}}{\rightleftharpoons}} Nu2^{-}DBUH^{+}$$

$$DBU + P2_H \underset{k_{-6}^{C}}{\overset{\mathrm{k}_6^{C}}{\rightleftharpoons}} P2^{-}DBUH^{+}$$

1,2-addition:

$$E + Nu2^{-}DBUH^{+} \overset{k_2^C}{\rightarrow} Int1^{-}DBUH^{+}$$

$$P1 + Nu2^{-}DBUH^{+} \overset{k_3^C}{\rightarrow} Int1a^{-}\ DBUH^{+}$$

$$E + P2^{-}DBUH^{+} \overset{k_7^C}{\rightarrow} Int2^{-}\ DBUH^{+}$$

$$P1 + P2^{-}DBUH^{+} \overset{k_8^C}{\rightarrow} Int2a^{-}\ DBUH^{+}$$

1,4-addition/elimination:

$$E + Int1^{-}DBUH^{+} \overset{k_4^C}{\rightarrow} P2H + E + DBU$$

$$E + Int1a^{-}DBUH^{+} \overset{k_5^C}{\rightarrow} P2H + P2H + DBU$$

$$E + Int2^{-}DBUH^{+} \overset{k_9^C}{\rightarrow} P3 + E + DBU$$

$$E + Int2a^{-}DBUH^{+} \overset{k_{10}^C}{\longrightarrow} P3 + P2H + DBU$$

Table S12. Optimal kinetic and thermodynamic parameters fitted according to the reaction 2 model.

Parameter	Key thresholds/relationships
K_1^B / M^{-1}	$K_1^B > 100$
K_6^B / M^{-1}	$K_1^B/K_6^B = 3.43$
k_1^B / $\mathrm{M}^{-1}\mathrm{s}^{-1}$	$k_1^B > 10^5$
k_6^B / $\mathrm{M}^{-1}\mathrm{s}^{-1}$	$k_6^B > 10^5$
k_2^B / $\mathrm{M}^{-1}\mathrm{s}^{-1}$	$k_2^B/k_3^B = 17.9$
k_3^B / $\mathrm{M}^{-1}\mathrm{s}^{-1}$	
k_7^B / $\mathrm{M}^{-1}\mathrm{s}^{-1}$	$k_2^B/k_7^B = 0.674$
k_8^B / $\mathrm{M}^{-1}\mathrm{s}^{-1}$	$k_2^B/k_8^B = 4.82$
k_4^B / $\mathrm{M}^{-1}\mathrm{s}^{-1}$	$k_4^B > 10^5$
k_5^B / $\mathrm{M}^{-1}\mathrm{s}^{-1}$	
k_9^B / $\mathrm{M}^{-1}\mathrm{s}^{-1}$	$k_9 > 10^5$
k_{10}^B / $\mathrm{M}^{-1}\mathrm{s}^{-1}$	

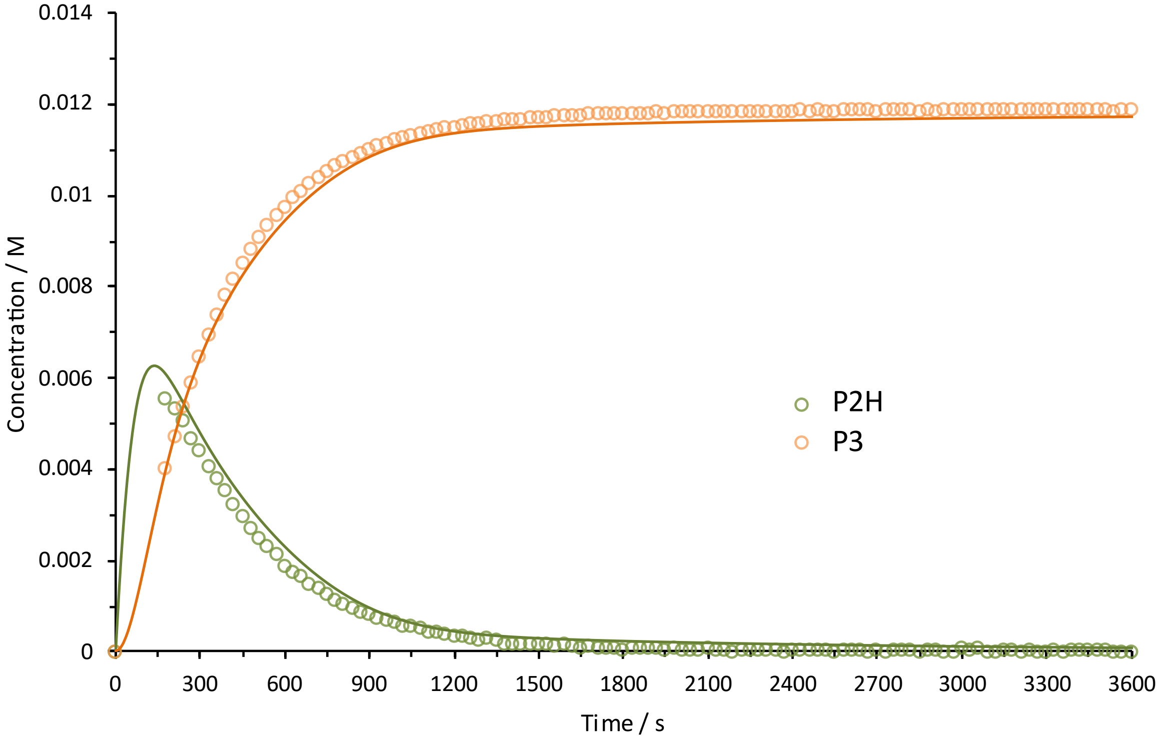

*Figure S84. Experimental (open shapes) and simulated (filled lines) temporal concentrations of **P2**$_H$ and **P3**, in the DBU catalysed reaction of **Nu2**$_H$ and **E** in the absence of **C**, under **E** Excess conditions.*

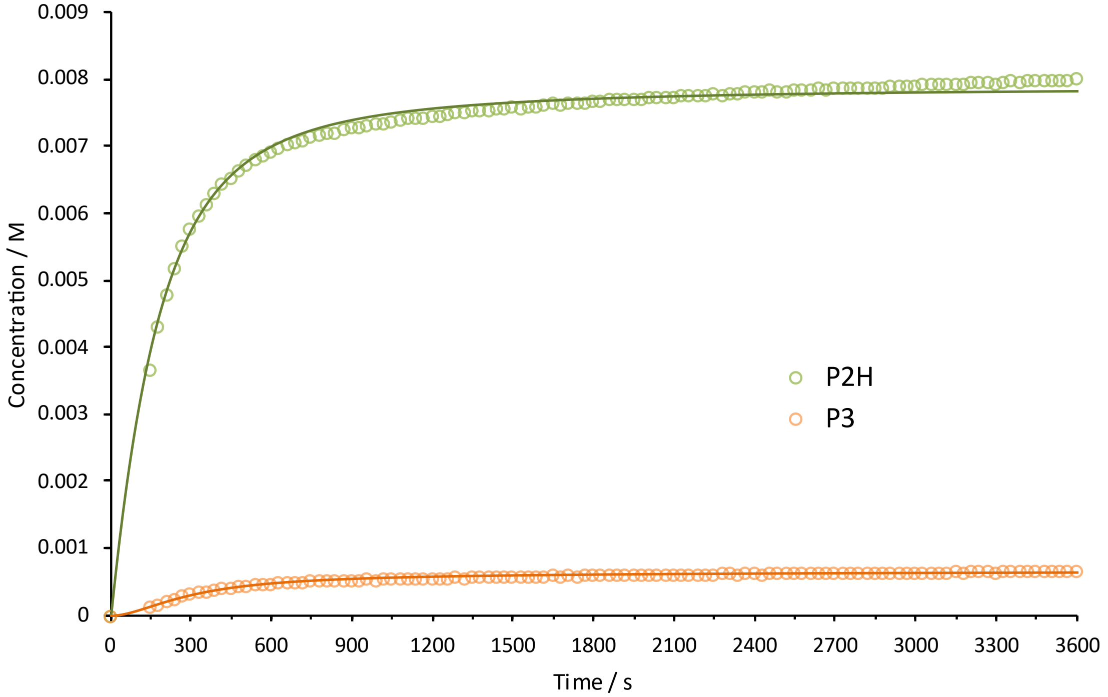

*Figure S85. Experimental (open shapes) and simulated (filled lines) temporal concentrations of **P2**$_H$ and **P3**, in the DBU catalysed reaction of **Nu2**$_H$ and **E** in the absence of **C**, under **Nu2**$_H$ excess conditions.*

S9. Eyring analysis

General Procedure:

Eyring analysis was performed on reaction 1 in which the **C** and DBU-only catalysed reactions were repeated at temperatures ranging from 273.15 to 298.15 K at least twice following the representative procedure outlined in section S4 with the concentrations shown in section Table S13 (cage catalysed) and Table S15 (DBU-only). The reactions were monitored using a pre-calibrated pseudo2D pulse sequence until reaction completion. All spectra were then processed in MNova version 14 with the following applied: automatic phase correction, Whittaker smoother baseline correction and solvent chemical shift referencing.

*Table S13: Initial concentrations of **E** and **Nu1**$_H$ used to acquire kinetics at different temperatures for Eyring analysis. Each row corresponds to a different experiment, and all experiments employ initial concentrations of 0.784 mM C and 0.273 mM DBU.*

T / K	**E** / mM	**Nu1**$_H$ / mM
273	1.22	6.35
273	1.99	5.62
278	1.84	8.53
278	2.74	9.81
283	1.71	8.02
283	2.55	10.0
288	1.79	7.71
288	2.14	8.13
293	1.30	7.00
293	2.23	8.75
298	1.38	6.51
298	2.11	8.65

The kinetics were measured at a range of temperatures by monitoring the formation of $P1_H$ and fitting to a modified pseudo 1st order rate equation:

$$y = A \cdot (1 - e^{-k_{obs}t}) + B$$

where k_{obs} is the observed rate constant (s^{-1}), A is the final product concentration and B is an offset for the initial concentration. The rate constant of the rate determining step k_2 could then be calculated based on the following assumptions:

$$rate = k_2[E][Nu1^- \subset C]$$

$$rate = k_{obs}[E]$$

$$k_{obs} = k_2[Nu1^- \subset C]$$

$$[Nu1^- \subset C] = [DBU]$$

For each run, the fitted rate constant k_{obs} was divided by the concentration of DBU used (0.273 mM) to obtain k_2. The subsequent analysis was conducted by linearising the Eyring equation and relating the activation parameters to the slope and intercept of a trendline fitted through each dataset, which is plotted as ln(k_2/T) vs 1/T.

$$k = \left(\frac{\kappa k_b T}{h}\right) e^{\frac{\Delta G^{\ddagger}}{RT}} \text{ and } \ln\frac{k}{T} = \left(\frac{-\Delta H^{\ddagger}}{R}\right)\frac{1}{T} + \left(\frac{\Delta S^{\ddagger}}{R} + \ln\frac{\kappa k_b T}{h}\right)$$

$$slope = \frac{-\Delta H^{\ddagger}}{R}$$

$$y\ intercept = \frac{\Delta S^{\ddagger}}{R} + \ln\left(\frac{k_b}{h}\right)$$

The Gibbs free energy of activation $\Delta G^{\ddagger}$ can be reconstructed using the relationship $\Delta G^{\ddagger} = \Delta H^{\ddagger} - T\Delta S^{\ddagger}$.

S9.1 Cage Catalysed Reaction

Table S13. The k_2 values and associated ln(k_2/T) values for subsequent Eyring analysis.

T / K	T^{-1} / K	k_{obs} / s^{-1}	k_2 / M^{-1} s^{-1}	ln(k_2/T)	average ln(k_2/T)
273	0.0037	2.60E-04	0.953	-5.658	
273	0.0037	1.50E-04	0.550	-6.208	-5.933
278	0.0036	3.81E-04	1.395	-5.295	
278	0.0036	4.32E-04	1.583	-5.168	-5.232
283	0.0035	6.04E-04	2.214	-4.851	
283	0.0035	0.00107	3.919	-4.280	-4.565
288	0.0035	8.54E-04	3.129	-4.522	
288	0.0035	9.99E-04	3.661	-4.365	-4.444
293	0.0034	0.00122	4.469	-4.183	
293	0.0034	0.00173	6.337	-3.834	-4.008
298	0.0034	0.00277	10.147	-3.380	
298	0.0034	0.00359	13.150	-3.121	-3.250

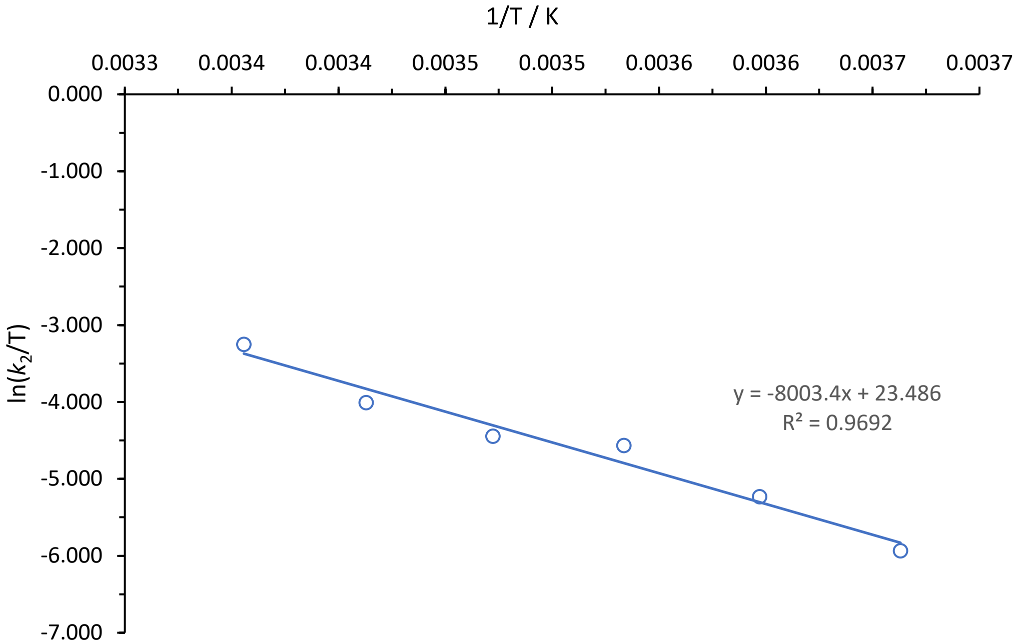

Figure S86. Linearised Eyring plot (ln(k_2/T) vs 1/T) of cage catalysed reaction 1 giving activation parameters $\Delta H^{\ddagger}$ = 15.9 kcal mol^{-1}, $T\Delta S^{\ddagger}$ = −1.25 kcal mol^{-1}, $\Delta G^{\ddagger}$ = 17.2 kcal mol^{-1} where T = 298.15 K.

S9.2 DBU-only reaction

The cage-free reaction was carried out according to a modified version of the procedure outlined in section S4. In order to monitor the cage-free reaction on a more convenient timescale, the concentrations of all components were increased 100-fold. The concentration of product **P1**$_H$ formed over time was again fitted to a modified pseudo 1st order rate equation, and the rate constant k_{obs} then divided by the concentration of DBU used (27.3 mM) to obtain k_2 for subsequent Eyring Analysis.

*Table S15: Initial concentrations of **E** and **Nu1**$_H$ used to acquire kinetics at different temperatures for Eyring analysis. Each row corresponds to a different experiment, and all experiments employ initial concentrations of 0.784 mM C and 0.273 mM DBU.*

T / K	**E** / mM	**Nu1**$_H$ / mM
273	311	1230
273	265	593
278	33.3	97.9
278	202	646
283	155	518
283	229	604
288	228	473
288	250	700
293	308	502
293	231	649
298	150	484
298	272	673

Table S14. The k_2 values values and associated ln(k_2/T) values for subsequent Eyring analysis.

T / K	T^{-1} / K	k_{obs} / s^{-1}	k_2/ M^{-1} s^{-1}	ln(k_2/T)	average ln(k_2/T)
273	0.0037	5.87E-05	2.15E-03	-11.75	
273	0.0037	9.12E-05	3.34E-03	-11.31	-11.5
278	0.0036	7.14E-05	2.61E-03	-11.57	
278	0.0036	1.40E-04	5.11E-03	-10.90	-11.2
283	0.0035	1.50E-04	5.48E-03	-10.85	
283	0.0035	1.89E-04	6.92E-03	-10.62	-10.7
288	0.0035	2.36E-04	8.63E-03	-10.42	
288	0.0035	2.73E-04	1.00E-02	-10.27	-10.3
293	0.0034	3.28E-04	1.20E-02	-10.10	
293	0.0034	3.04E-04	1.11E-02	-10.18	-10.1
298	0.0034	4.61E-04	1.69E-02	-9.78	
298	0.0034	6.10E-04	2.24E-02	-9.50	-9.6

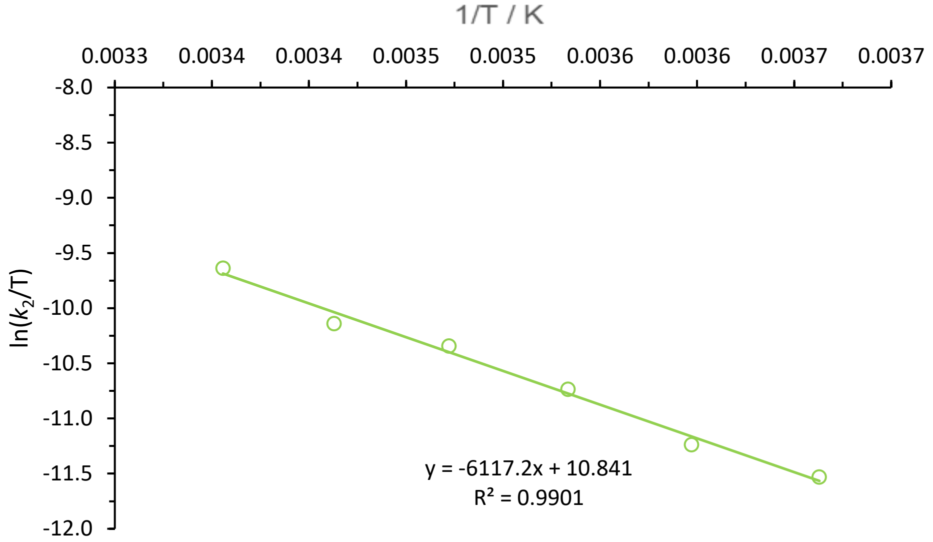

Figure S87. Linearised *Eyring plot* (ln(k_2/T) vs 1/T) *of cage catalysed reaction 1* giving activation parameters $\Delta H^{\ddagger}$ *= 12.2 kcal mol*$^{-1}$*,* $T\Delta S^{\ddagger}$ *= −8.75 kcal mol*$^{-1}$*,* $\Delta G^{\ddagger}$ *= 20.9 kcal mol*$^{-1}$ *where T = 298.15 K.*

S9.3 Error Analysis

To estimate the error associated with the activation parameters and establish the difference between the cage and cage-free reactions, a Monte-Carlo approach was taken in which a ±20% random error was applied to the measured k_2 values and a ±1% random error was applied to the temperature (K). This approach was used to generate an ensemble of 100 simulated ln(k_2/T) vs 1/T datasets. These simulated datasets were then subjected to the same linearised Eyring analysis, giving a distribution of activation parameters $\Delta H^{\ddagger}$ and $\Delta S^{\ddagger}$. From this collection, the standard error (SE) was calculated according to

$$SE_Y \approx \frac{\sigma_Y}{\sqrt{n}}$$

Where σ_Y is the measured standard deviation of a parameter Y and n the sample size, 100. Assuming $\Delta H^{\ddagger}$ and $\Delta S^{\ddagger}$ are uncorrelated, the error associated with $\Delta G^{\ddagger}$ (at 298.15 K) can be propagated using

$$SE_{\Delta G^{\ddagger}} = \sqrt{SE_{\Delta H^{\ddagger}}^2 + T^2 SE_{\Delta S^{\ddagger}}^2}$$

where T = 298.15 K. Since the standard errors reflect only one standard deviation from the mean value (~68% of a normal distribution) they can be multiplied by 3 to give an approximately total standardized error, which reflects ~99.7% of the distribution.

S9.3.1 Cage catalysed reaction 1

Table S15. Activation parameters and associated errors for cage catalysed reaction 1 where T = 298.15 K. Values designated with a superscript "a" are derived from the error propagation defined in section 9.3.

Quantity	$\Delta H^{\ddagger}$ / kcal mol^{-1}	$\Delta S^{\ddagger}$ / e.u	$T\Delta S^{\ddagger}$ / kcal mol^{-1}	$\Delta G^{\ddagger}$ / kcal mol^{-1}
Calculated value	15.92	-0.55	-0.16	16.08
σ_Y (Monte-Carlo)	1.39	4.90	1.46	
SE (68%)	0.14	0.49	0.15	[a]0.20
$3SE$ (99.7%)	0.42	1.47	0.44	[a]0.60

S9.3.2 DBU-only reaction 1

Table S16. Activation parameters and associated errors for DBU-only reaction 1 where T = 298.15 K. Values designated with a superscript "a" are derived from the error propagation defined in section 9.3.

Quantity	$\Delta H^{\ddagger}$ / kcal mol^{-1}	$\Delta S^{\ddagger}$ / e.u	$T\Delta S^{\ddagger}$ / kcal mol^{-1}	$\Delta G^{\ddagger}$ / kcal mol^{-1}
Calculated value	12.17	-25.70	-7.66	19.83
σ_Y (Monte-Carlo)	1.24	4.32	1.29	
SE (68%)	0.12	0.43	0.13	0.18
$3SE$ (99.7%)	0.37	1.30	0.39	0.54

S9.3.3 Summary of Eyring analysis

Table S17. Experimentally calculated $\Delta H^{\ddagger}$, $T\Delta S^{\ddagger}$ (where T = 298.15 K) and $\Delta G^{\ddagger}$ and associated errors for cage-catalysed and base-catalysed reaction 1.

Reaction	$\Delta H^{\ddagger}$ / kcal mol^{-1}	$T\Delta S^{\ddagger}$ / kcal mol^{-1}	$\Delta G^{\ddagger}$ / kcal mol^{-1}
Cage-catalysed	15.9 ± 0.4	-0.2 ± 0.4	16.1 ± 0.6
DBU-only	12.2 ± 0.4	-7.7 ± 0.4	19.8 ± 0.5

S10. Effect of alternative bases and a description of rate enhancement by cage

S10.1 Estimation of K_1^B and K_1^C

In order to estimate the extent to which **C** modulates the acidity of nucleophile **Nu1**$_H$, a baseline measurement of the acid-base equilibrium in the absence of **C** is needed. Using DBU as a base, the magnitude of this equilibrium constant for can be directly calculated from ^{1}H NMR titration (section 7.1). For the weaker bases diethyl aniline and di-(tert-butyl)-pyridine this is not the case due to much lower expected values of K_1^B.

To estimate the values of K_1^B for **Nu1**$_H$ and these bases, the initial rate of base-catalysed Michael addition of **Nu1**$_H$ and **E** was used. By assuming a similar mechanism to the DBU-catalysed Michael addition (Figure S74) and applying the pre-equilibrium approximation (k_1^B , $k_{-1}^B \gg k_2^B$), a rate equation can be defined.

$$K_1^B = \frac{[BH^+Nu1^-]}{[Nu1_H][B]}$$

$$K_1^B \approx \frac{[BH^+Nu1^-]}{[Nu1_H]_0([B]_T - [BH^+Nu1^-])}$$

$$\frac{[BH^+Nu1^-]}{[B]_T} \approx \frac{K_1^B[Nu1_H]_0}{1 + K_1^B[Nu1_H]_0}$$

$$v_0^B = k_2^B[BH^+Nu1^-]_0[E]_0$$

$$v_0^B \approx \frac{K_1k_2[Nu1_H]_0[E1]_0[B]_T}{1 + K_1^B[Nu1_H]_0}$$

If k_2^B is assumed to be consistent between the different bases (i.e. variation in basicity between the bases is the only contributor to any variation in initial rate), the approximate rate equation can be rearranged to give K_1^B as a function of v_0, $[Nu1_H]_0$, $[E]_0$, $[B]_T$ and k_2^B.

$$K_1^B = \frac{v_0^B}{[Nu1_H]_0\,(\,k_2[E]_0[B]_T - v_0^B\,)}$$

Table S20. Experimental initial rates of Michael additions between ***Nu1****$_H$ and* ***E*** *catalysed by different bases in the presence and absence of cage. In the cases where no cage is present the values for K_1^B are obtained from the equation above. In the case where cage is present, the K_1 values are obtained from fitting to the kinetic model 4 described in section S6.1.4.*

Basic species	Initial rate / M s^{-1}	$[Nu1_H]_0$ / M	$[E]_0$ / M	$[B]_T$ / M	K_1 / M^{-1}
DBU	1.23×10^{-4}	0.083	0.021	0.0016	4.2×10^{5}
Diethylaniline	7.15×10^{-8}	1.05	0.37	0.027	3.1×10^{-4}
Di-(tert-butyl)-pyridine	4.90×10^{-9}	1.00	0.39	0.027	2.1×10^{-5}
Cage + DBU	-	0.0105	0.00395	0.000237	3.8×10^{6}
Cage + Diethylaniline	-	0.00697	0.00316	0.00027	6.3×10^{2}
Cage + Di-(tert-butyl)-pyridine	-	0.0083	0.0030	0.00027	2.4×10^{2}

General Procedure:

The reactions in the presence and absence of **C** were carried out according to the representative procedure outlined in section SS4, with the reactant concentrations shown in section SS4.2.1 and section S4.2.2. The reactions were monitored using a pre-calibrated pseudo2D pulse sequence. All spectra were processed in MNova version 14 with the following applied: automatic phase correction, baseline correction *via* a Whittaker smoother method and solvent chemical shift referencing.

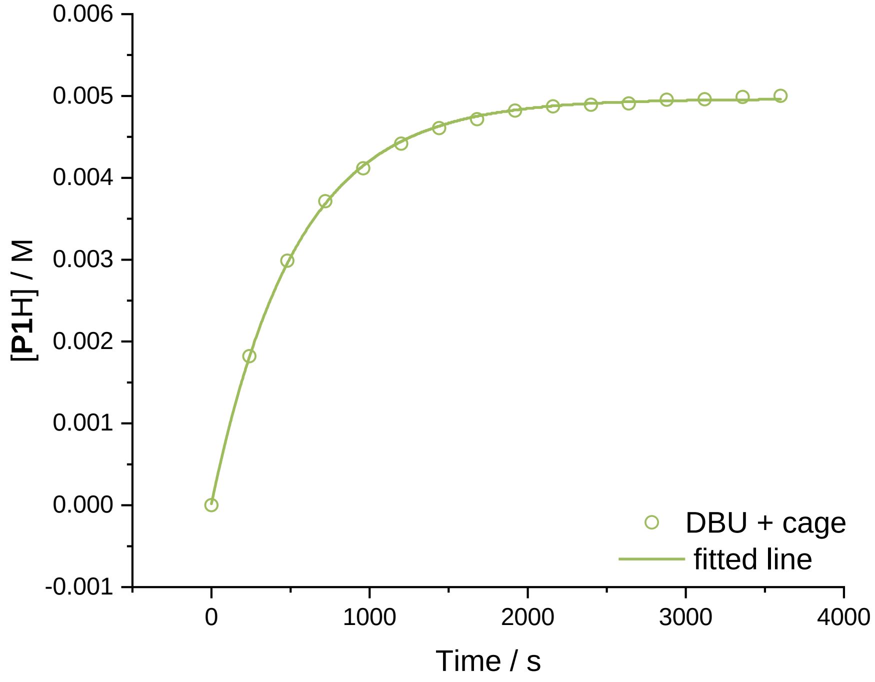

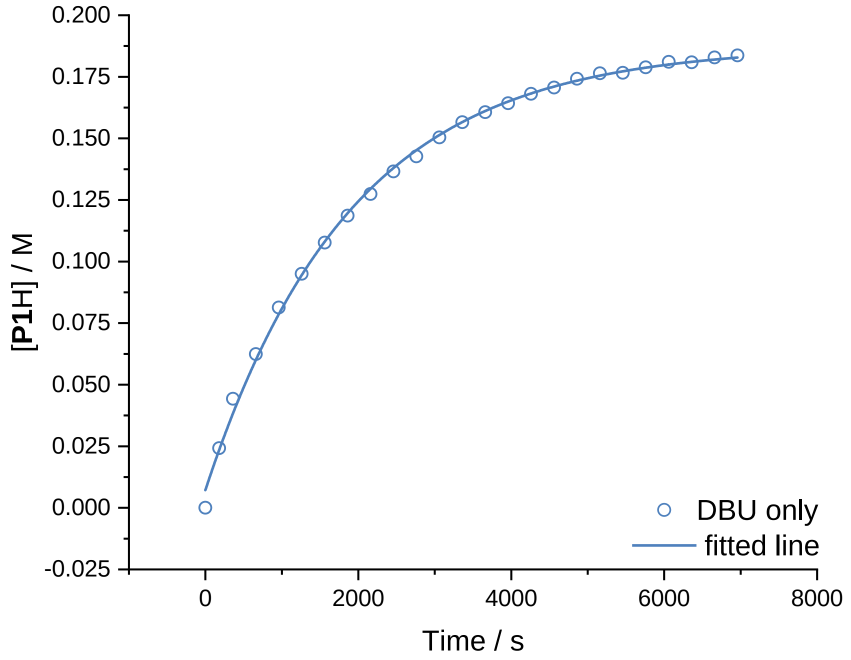

*Figure S88. Experimental (open shapes) and fitted (filled line) concentrations of **P1**$_H$, in the cage (top) and DBU only (bottom) catalysed reaction of **Nu1**$_H$ and **E**.*

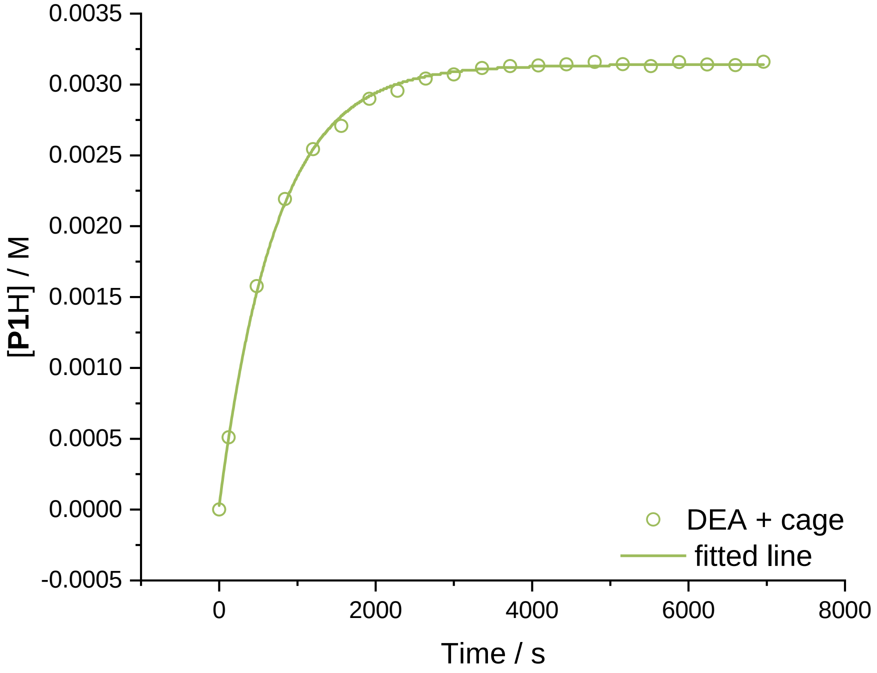

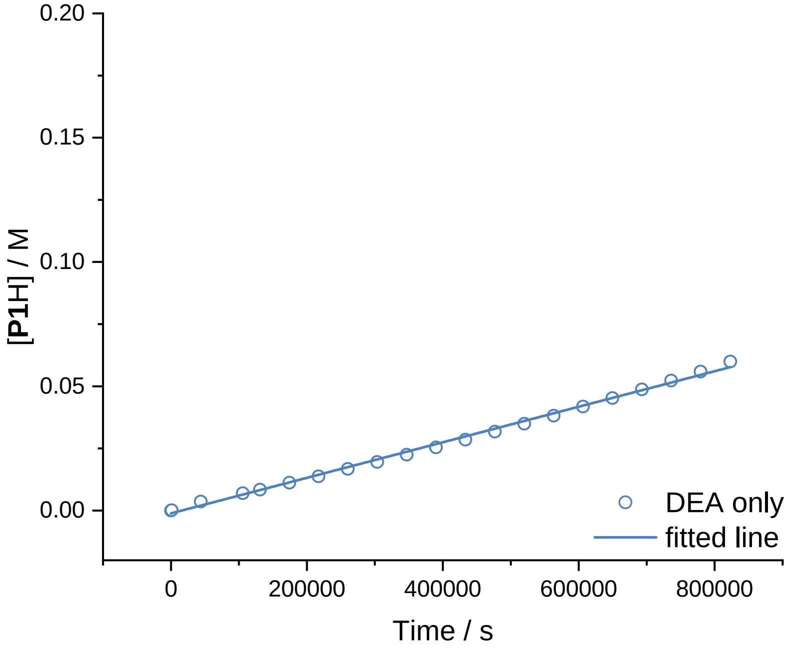

*Figure S89. Experimental (open shapes) and fitted (filled line) concentrations of **P1**$_H$, in the cage (top) and DEA only (bottom) catalysed reaction of **Nu1**$_H$ and **E**.*

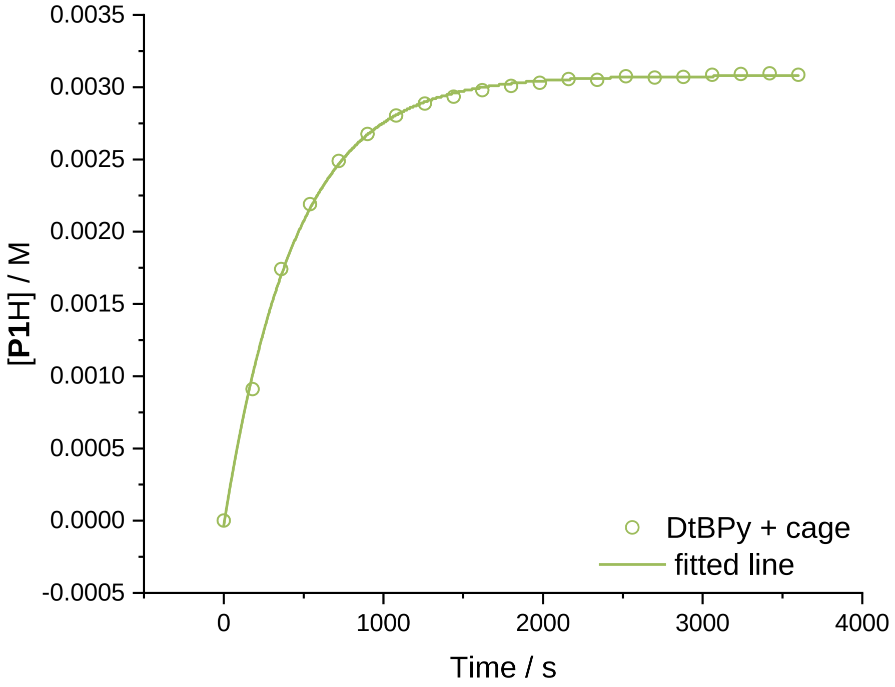

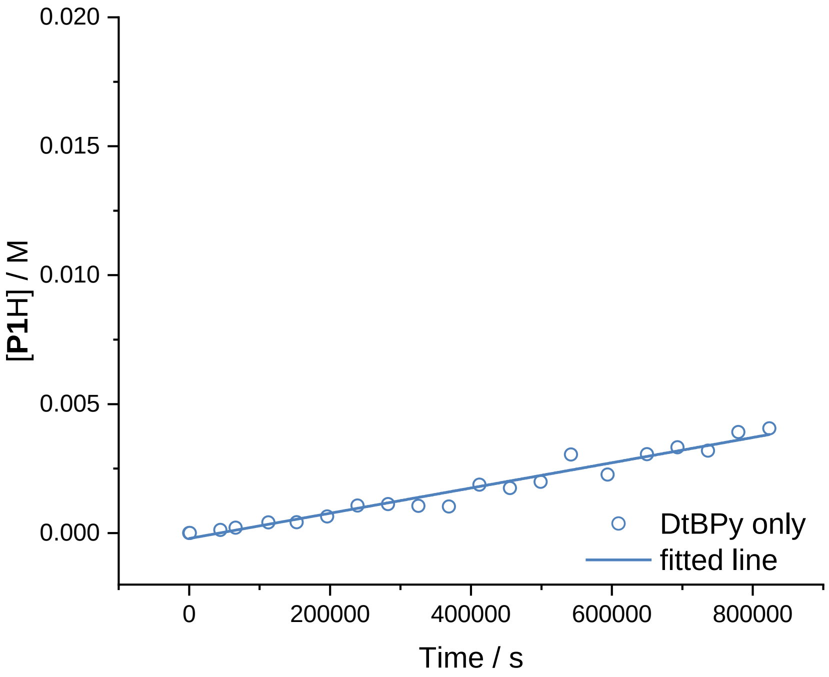

*Figure S90. Experimental (open shapes) and fitted (filled line) concentrations of **P1**$_H$, in the cage (top) and Di-tert-butylpyridine only (bottom) catalysed reaction of **Nu1**$_H$ and **E**.*

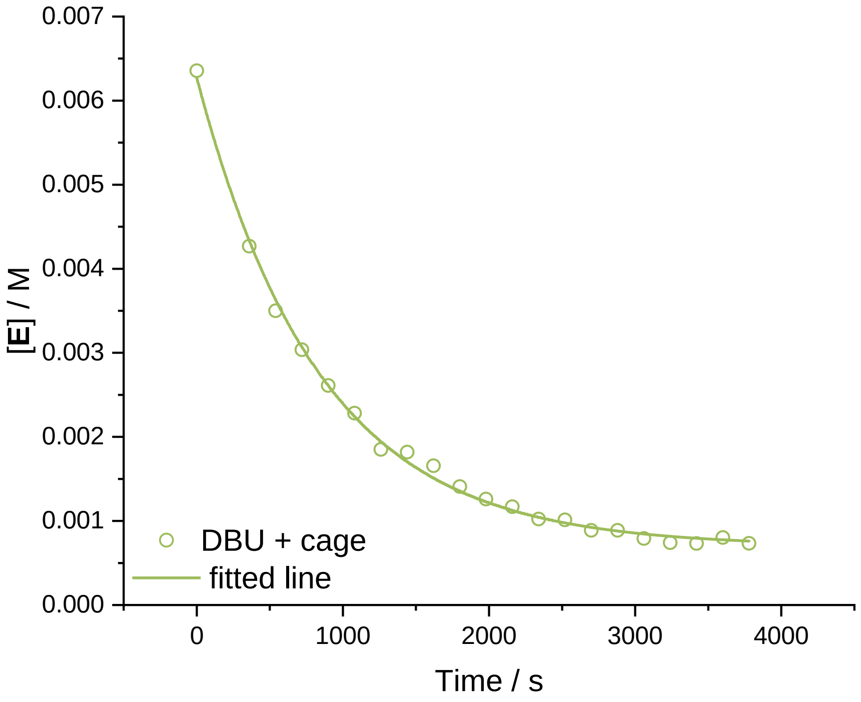

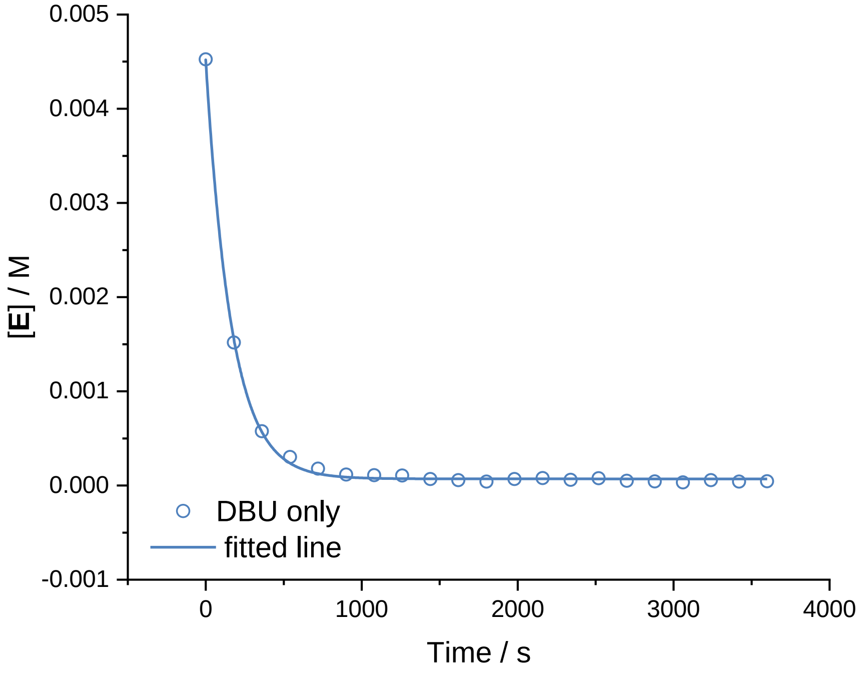

*Figure S91. Experimental (open shapes) and fitted (filled line) concentrations of **E**, in the cage (top) and DBU only (bottom) catalysed reaction of **Nu2**$_H$ and **E**.*

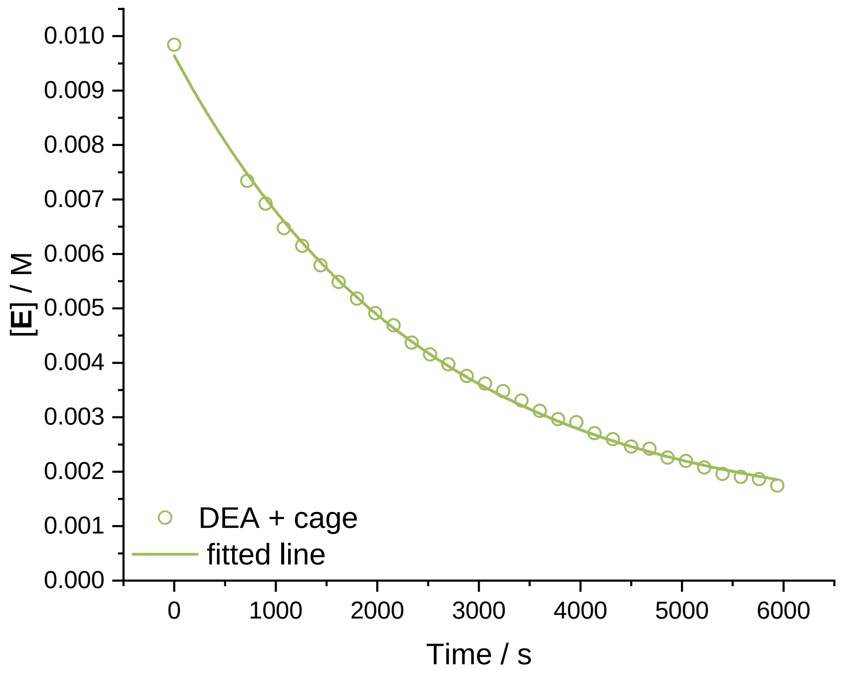

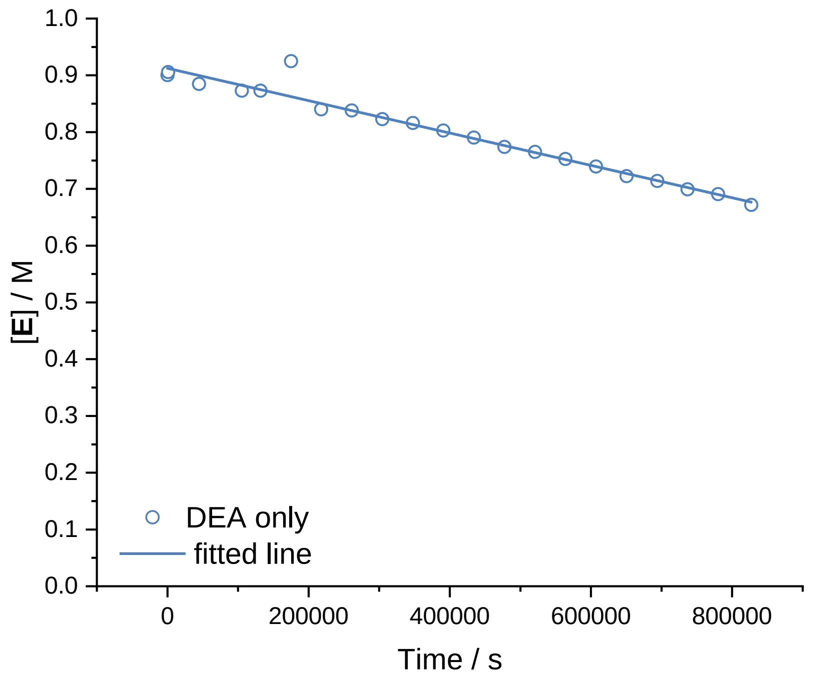

*Figure S92. Experimental (open shapes) and fitted (filled line) concentrations of **E**, in the cage (top) and DEA only (bottom) catalysed reaction of **Nu2**$_H$ and **E**.*

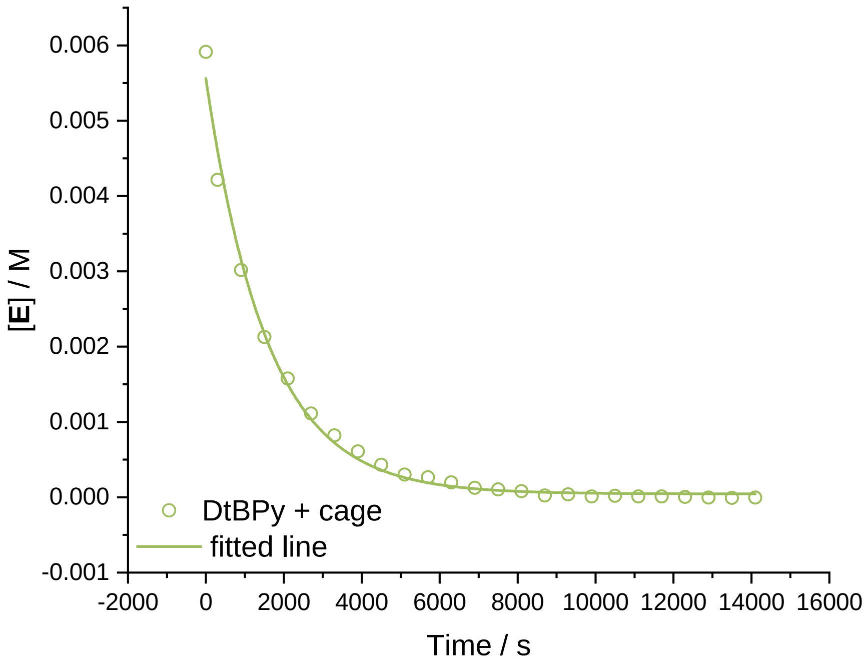

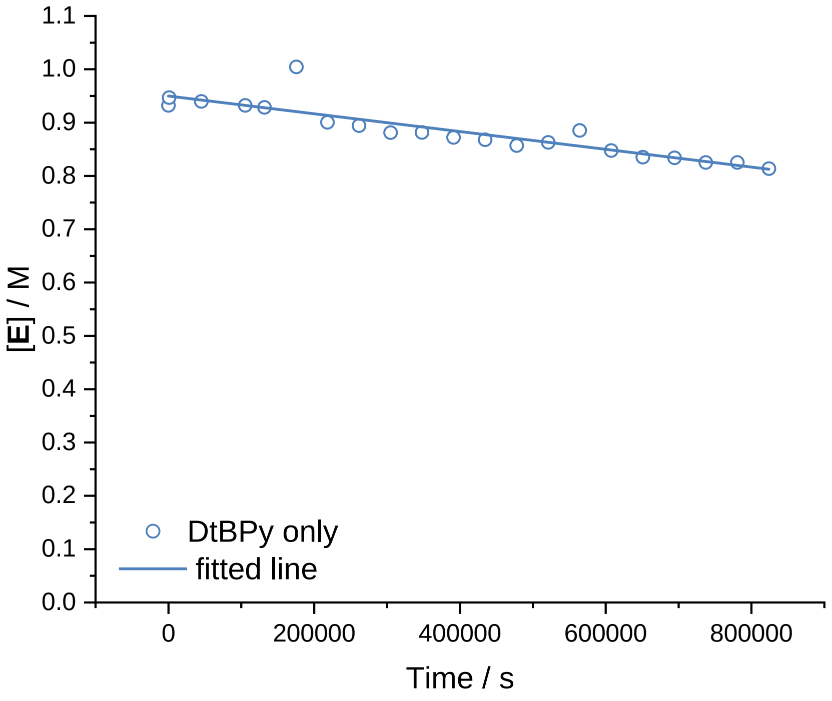

*Figure S93. Experimental (open shapes) and fitted (filled line) concentrations of **E**, in the cage (top) and Di-tert-butylpyridine only (bottom) catalysed reaction of **Nu2**$_H$ and **E**.*

S10.2 Extent of acceleration k_{rel}^{max} for cage-catalysed Michael additions

As discussed in the manuscript, there are two mechanisms by which cage C can enhance the rate of Michael addition reactions: Acceleration of the addition step ($k_2^C > k_2^B$) and enhancement of the acidity of pronucleophiles by anion complexation ($K_1^C > K_1^B$). In order to quantify the extent to which adding cage enhances the rate by both processes, the rates of reaction must be compared in the presence and absence of cage.

If equilibration is assumed to be rapid, an approximate rate law can be derived for the mechanism in the presence of cage (denoted with a superscript C),

$$v^C \approx \frac{K_1^C k_2^C [Nu1_H][E][Cage]_{total}}{\frac{[BH^+]}{[B]} + K_1^C[Nu1_H] + K_{-3}^C[P1_H]}$$

When considering only the initial rates, product inhibition can be assumed to be negligible leading to a simplified equation,

$$v^C \approx \frac{K_1^C k_2^C [Nu1_H][E][Cage]_{total}}{\frac{[BH^+]}{[B]} + K_1^C[Nu1_H]}$$

To approximate the effect of base speciation on the rate law, the following system of equilibrium equations are solved for $[B]$, $[BH^+]$, $[Nu1^- \subset C]$ and $[C]$,

$$[B]_{total} = [B] + [BH^+]$$

$$[C]_{total} = [C] + [Nu1^- \subset C]$$

$$[BH^+] = [Nu1^- \subset C]$$

$$K_1^C = \frac{[BH^+][Nu1^- \subset C]}{[C][Nu1_H][B]}$$

Since the system is quadratic, there are a pair of solutions for each species, but only one of the solutions guarantees positive real solutions for positive real parameters. These solutions for $[B]$ and $[BH^+]$ are,

$$[B] = \frac{[B]_{total}(K_1^C[Nu1_H] - 2) - K_1^C[Nu1_H][C]_{total} + \sqrt{K_1^C[Nu1_H](4[B]_{total}[C]_{total} + K_1^C[Nu1_H]([B]_{total} - [C]_{total})^2)}}{2K_1^C[Nu1_H] - 2}$$

$$[BH^+] = \frac{K_1^C[Nu1_H]([B]_{total} + [C]_{total}) - \sqrt{K_1^C[Nu1_H](4[B]_{total}[C]_{total} + K_1^C[Nu1_H]([B]_{total} - [C]_{total})^2)}}{2K_1^C[Nu1_H] - 2}$$

Substituting into the expression for v^C gives a new rate equation,

$$v^C \approx \frac{K_1^C k_2^C[Nu1_H][E]([B]_{total} + [C]_{total}) - k_2^C[E]\sqrt{K_1^C[Nu1_H](4[B]_{total}[C]_{total} + K_1^C[Nu1_H]([B]_{total} - [C]_{total})^2)}}{2K_1^C[Nu1_H] - 2}$$

For comparison with the base catalysed Michael addition, a pre-equilibrium rate law can be derived for the reaction (superscript B denotes base only)

$$v^B \approx \frac{K_1^B k_2^B [Nu1_H][E][B]_{total}}{1 + K_1^B [Nu1_H]}$$

In the case of a cage and base catalysed Michael addition, the sensitivity of the initial rate to base concentration $[B]_{total}$ saturates when the amount of base exceeds the concentration of the cage catalyst used, while the rate in the absence of cage v^B (where the base itself is the limiting catalytic species) continues to increase.

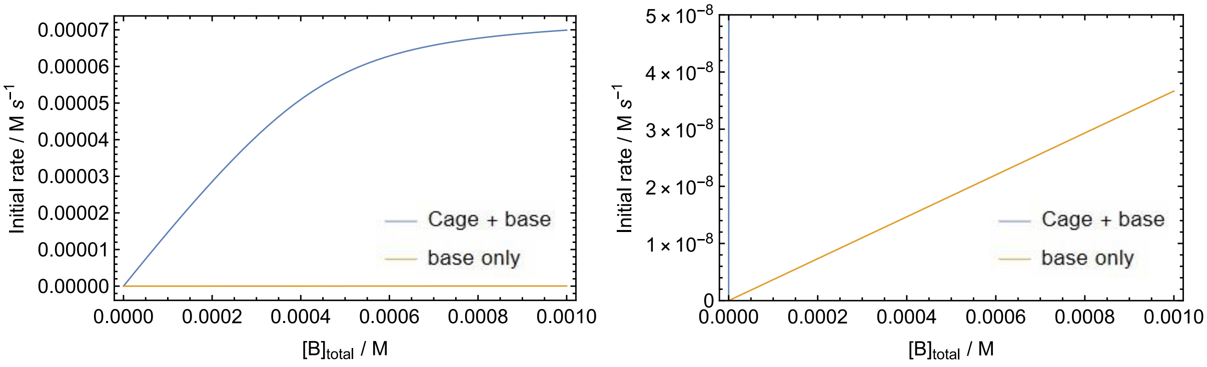

Figure S94: Simulated rate vs concentration profiles for rate expressions v^B and v^C ($[C]_{total}$ = 0.0005M) showing saturation behaviour in the cage-mediated process and first order behaviour in the cage-free process.

To reconcile the different roles of the base between the two scenarios, the initial gradients $\left(\frac{dv_0^C}{d[B]_{total}} \; and \; \frac{dv_0^B}{d[B]_{total}}\right)$ of the rate concentration plots are taken as a measure of the sensitivity to base concentration in each case. In the cage catalysed process, this is the furthest point from catalyst saturation, and describes the full extent to which the cage enhances the rate of a given base catalysed Michael addition reaction. Differentiating the expressions for v^C and v^B with respect to $[B]_{total}$ gives

$$\frac{dv_0^C}{d[B]_{total}} \approx k_2^C [E]$$

$$\frac{dv_0^B}{d[B]_{total}} \approx \frac{K_1^B k_2^B [Nu1_H][E]}{1 + K_1^B [Nu1_H]}$$

The maximum enhancement of cage k_{rel}^{max} can then be expressed as the ratio $\frac{dv_0^C}{d[B]_{total}} \Big/ \frac{dv_0^B}{d[B]_{total}}$, written as $\frac{dv_0^C}{dv_0^B}$

$$k_{rel}^{max} = \frac{dv_0^C}{dv_0^B} \approx \left(\frac{k_2^C}{k_2^B}\right)\left(1 + \frac{1}{K_1^B [Nu1_H]}\right)$$

S10.3 Alternative k_{rel}^{max} derivation by approximation

Starting with the same initial rate expression for the cage-catalysed reaction.

$$v^C \approx \frac{K_1^C k_2^C [Nu1_H][E][Cage]_{total}}{\frac{[BH^+]}{[B]} + K_1^C [Nu1_H]}$$

Noting that $\frac{[BH^+]}{[B]} = \frac{K_1^C [Nu1_H][C]}{[Nu1^- \subset C]}$, substitution into the expression for v^C gives

$$v^C \approx \frac{K_1^C k_2^C [Nu1_H][E][Cage]_{total}}{\frac{K_1^C [Nu1_H][C]}{[Nu1^- \subset C]} + K_1^C [Nu1_H]}$$

Cancelling $K_1^C [NuH]$ gives

$$v_0^C \approx \frac{k_2^C [E][Cage]_{total}}{\left(\frac{[C]}{[Nu1^- \subset C]} + 1\right)}$$

When $[B]_{total} \ll [Cage]_{total}$ the fraction $\frac{[C]}{[Nu1^- \subset C]}$ is very large, and the denominator can be truncated

$$v_0^C \approx \frac{k_2^C [E][Cage]_{total}}{\left(\frac{[C]}{[Nu1^- \subset C]}\right)}$$

$$v_0^C \approx \frac{k_2^C [E][Cage]_{total}[Nu1^- \subset C]}{[C]}$$

When $[B]_{total} \ll [Cage]_{total}$ and $K_1^C \gg 1$, $[C] \approx [Cage]_{total}$ and $[Nu1^- \subset C] \approx [B]_{total}$

$$v_0^C \approx \frac{k_2^C [E][Cage]_{total}[B]_{total}}{[Cage]_{total}}$$

Cancelling $[Cage]_{total}$ gives an approximate expression for v_0^C

$$v_0^C \approx k_2^C [E][B]_{total}$$

Using the previously derived v_0^B expression, k_{rel}^{max} can be defined by taking the ratio v_0^C/v_0^B , resulting in the same equation as above

$$v_0^B \approx \frac{K_1^B k_2^B [Nu1_H][E][B]_{total}}{1 + K_1^B [Nu1_H]}$$

$$k_{rel}^{max} = \frac{v_0^C}{v_0^B} \approx \left(\frac{k_2^C}{k_2^B}\right)\left(1 + \frac{1}{K_1^B [Nu1_H]}\right)$$

S11. Computational methods

S11.1 Quantum calculations

QM calculations were done using ORCA (v 5.0.3).[2,3] Geometry optimisations were performed with the PBE0 functional[3] and the Ahlrichs def2-SVP basis set.[5] Dispersion effects were accounted for by the Becke-Johnson damped D3 dispersion correction (D3BJ).[6,7] Single-point calculations were carried out using M06-2X functional[7] with a def2-TZVP basis set. The selection of this functional was based on its demonstrated good performance when applied to supramolecular complexes,[9] as well as on our benchmark study for the uncatalysed reactions using the CPCM(DCM)-DLPNO-CCSD(T)/def2-TZVPP as a reference (RMSE = 1.9 kcal mol^{-1}; We also used tested CPCM(DCM)-ωB97X/def2-TZVP, which performs slightly worse than the M06-2X. and **Table**). Solvent effects were accounted for by using the CPCM implicit solvent model[10] with parameters corresponding to dichloromethane (DCM); further comparisons were also made with the SMD model, yielding similar results (**Table**). Thermal contributions were computed at 298 K using the quasi-rigid-rotor-harmonic oscillator on CPCM(DCM)-PBE0-D3BJ/def2-SVP optimised geometries.[11] For the cage-free reactions, the transition state (TS) was located using *autode*.[12] For the reaction within the cage, a guess TS was generated by docking the product state inside the cage using *cgbind*.[13] Subsequently, a 1D scan was performed along the C-C forming bond at the CPCM(DCM)-PBE0-D3BJ/def2-SVP level of theory. The highest energy point in this profile was further optimised to a TS at the same level of theory. A possible reactant complex was also identified by following the C-C displacement in the direction of the reactants.

S11.2 Molecular dynamics

MD simulations were performed with GROMACS (v 2019.4).[14,15] The systems were simulated in a cubic box containing approximately 500 DCM molecules, resulting in a box size of ~ 4.0 nm × 4.0 nm × 4.0 nm. DCM parameters were obtained from VirtualChemistry.org.[16] Additionally, 17 OPC[17] water molecules were added as impurities to DCM. The systems were neutralised with BArF$^-$as a counter ion and energy-minimised for 5000 steps or until the maximum force was below 10 kJ mol^{-1} nm^{-1}. Three replicas were initiated from the minimised system by assigning random velocities at 300.0 K. Each replicate was

subjected to 20 ps (2 fs stepsize) NVT and 20 ps (2 fs stepsize) NPT equilibration at 300.0 K and 1.0 bar before a production run of 100 ns (2 fs stepsize), during which coordinates were saved every 10 ps. During equilibrations, temperature and pressure were maintained using the Berendsen thermostat and barostat.[18] Production runs were performed using the v-rescale thermostat[19] and Parinello-Rahman barostat.[20] Long-range electrostatics were treated using smooth particle mesh Ewald.[21,22] For van der Waals interactions and short-range electrostatics, a 1.0 nm cut-off was used.

Analyses were performed using MDAnalysis.[23,24] To quantify the number of solvent molecules inside the cage cavity, a dynamic cut-off from the centre of mass (CoM) of the cage was defined, and all molecules with CoM within this cut-off were considered within the cavity. Given that the cage cavity changes in size and shape during simulation, we applied a dynamic cut-off, defined as the minimum distance between the cage CoM and any of its atoms, which during the simulations is in the range of 3.18−3.85 Å (**Figure**).

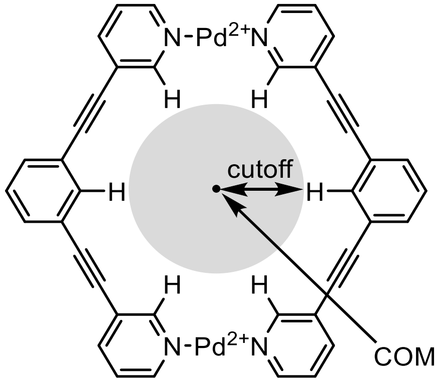

Figure S95. The solvent molecules are considered inside the cage if their CoM is within the cut-off from cage CoM. The cut-off is defined as the distance from CoM to the cage's closest atom.

Cage parameters were obtained using antechamber[25] and MCPB.py protocols,[26] which uses GAFF force-field. Parameters for the organic molecules *$Nu1_H$, $Nu2_H$, E and H^+DBU* were obtained using antechamber with GAFF parameters. Initial parameters for *$Nu1^-$ and $Nu2^-$ w*ere also obtained in this manner. However, their geometries were found to differ significantly from those optimised using QM calculations. Therefore, bonds and angles were obtained using Cole's modified Seminario method[27] from the Hessian matrix obtained at the PBE0-D3BJ/def2-SVP level of theory. Additionally, for $Nu1^-$, the dihedral angle involving the nitrate was corrected to match the QM potential computed at the PBE0-D3BJ/def2-SVP level of theory. Initial parameters for $BArF^-$ were obtained by antechamber for the isoelectronic neutral structure CArF, where the boron atom is substituted for carbon. The carbon parameters were replaced with boron parameters obtained from the literature,[28] initially developed for MM2 force-field[29] and later adapted and tested with AMBER force-field. Subsequently, bonds and angles were updated for optimised $BArF^-$ structure by the Seminario method (analogously to $Nu1^-$ and $Nu2^-$). For all molecules, partial charges were obtained by restrained electrostatic potential (RESP).[30] The force-field parameters are included as part of SI.

S12. Additional computational results

Cavity solvation. The MD simulations of cage in DCM solvent showed that on average 2 DCM molecules inside the cavity (**Figure a,b**).

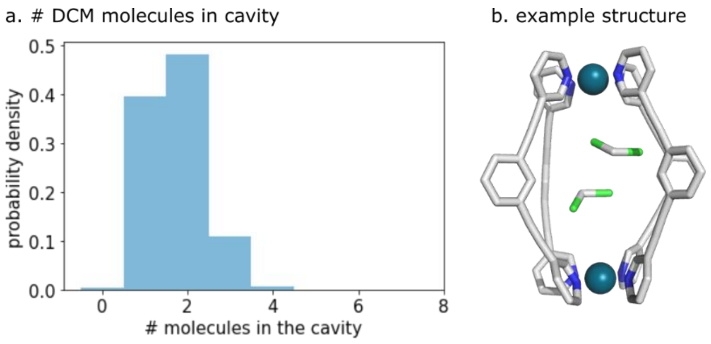

Figure S96. (a) Histogram of a number of solvent (DCM) molecules inside the cage. (b) Example structure of cage with 2 DCM molecules

S12.1 Reaction 1

S12.1.1Cage-free reaction

MD simulations were conducted to study the behaviour of the substrate(s) in solution and determine their interaction with the solvent (**Figure**), which indicates a strong interaction of the nucleophile with water molecules (**Figure**). The radial distribution function (RDF) analysis between Nu1⁻ oxygen atoms and water oxygens revealed a peak at approximately 2.8 Å and the first minimum at 3.4 Å. Beyond this distance, the solvent structure around the nucleophile became less well-defined. Therefore, a cut-off of 3.4 Å was used to determine whether water interacted with Nu1⁻ during the simulation. The analysis showed that, on average, Nu1⁻ directly interacts with four water molecules (**Figure b**).

Subsequently, four frames were extracted from the MD simulations, each containing Nu1⁻ interacting with four water molecules. QM free energy calculations indicate that the formation of the complex, Nu1⁻ · 4 H_2O, was endergonic relative to the separated species (formation of lowest energy complex ΔG = 11.9 kcal mol^{-1}; **Figure c; Table**). Consequently, Nu1⁻ was identified as the reactive species for further DFT calculations.

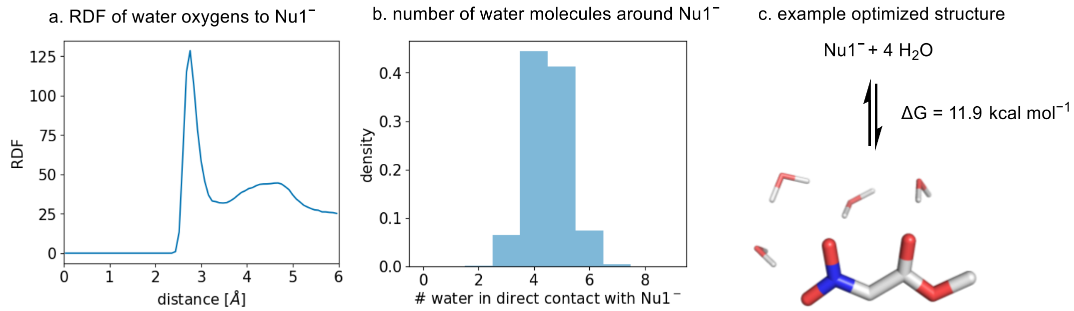

Figure S97. Coordination of Nu1− by water obtained from MD simulations. (a) RDF of oxygen atoms from Nu1− and water. (b) Histogram of a number of water molecules within 3.4 Å from Nu1⁻ oxygen atoms. (c) Lowest conformer of Nu1⁻ · 4 H_2O complex and its free energy of formation calculated by DFT.

Reaction barrier. Analysis of the free energy profile revealed an activation barrier of $\Delta G^{\ddagger}$ = 19.2 kcal mol^{-1} relative to the separated species, Nu1⁻ and E (**Table** and **Figure**). This value is consistent with the barrier obtained from kinetic studies of 20 kcal mol^{-1} at 298 K reported in this work. The formation of the reactant complex was found to be unfavourable (ΔG=8.0 kcal mol^{-1}) and accounted for about 40% of the energy

required to reach the TS. As we will see later, an important contribution from the cage is reducing this entropic cost.

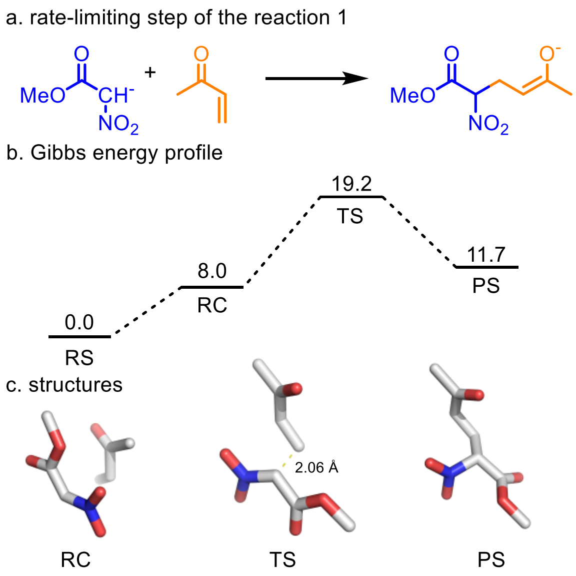

Figure S98. QM analysis of the rate-limiting step of DBU-catalyzed reaction. (a) studied reaction, (b) Gibbs energy profile in kcal/mol, (c) optimised structures of reactant complex (RC), transition state (TS), and product state (PS). Calculations on CPCM(DCM)-M06-2X/def2-TZVP//CPCM(DCM)-PBE0-D3BJ/def2-SVP level of theory.

Table S21. QM calculations of the cage-free ***reaction 1****. Geometries optimisations and thermochemical contributions were calculated on the CPCM(DCM)-PBE0-D3BJ/def2-SVP level of theory.*

	CPCM(DCM)-DLPNO-CCSD(T)/def2-TZVPP	CPCM(DCM)-ωB97X/def2-TZVP	CPCM(DCM)-M06-2X/def2-TZVP			
	ΔE [kcal mol^{-1}]	ΔE [kcal mol^{-1}]	ΔE [kcal mol^{-1}]	ΔH [kcal mol^{-1}]	−TΔS [kcal mol^{-1}]	ΔG [kcal mol^{-1}]
RS (Nu1− + E)	0	0.0	0.0	0.0	0.0	0.0
RC (Nu1− · E)	-4.3	-2.9	-3.4	-2.1	10.1	8.0
TS	7.7	10.1	6.7	7.7	11.5	19.2
PS	-5.5	-2.3	-2.4	-0.2	11.9	11.7
	MAE	2.4	1.7			
	RMSE	2.5	2.0			

S12.1.2Benchmark studies

We validated the choice of the CPCM(DCM)-M06-2X/def-TZVP level of theory for single-point calculations by comparing electronic energies with reference method CPCM-DLPNO-CCSD(T)/def2-TZVPP (**Table**). We also used tested CPCM(DCM)-ωB97X/def2-TZVP, which performs slightly worse than the M06-2X. CPCM was used as it enabled the calculation of analytical frequencies in ORCA v 5.0.3, which was not available for the SMD model in this version. Still, a comparison of electronic energies for **reaction 1** obtained using this model compared to the SMD model, which we have used previously[31] revealed a small difference (**Table**).

We also compared single-point energies calculated using def2-TZVP and def2-QZVP (**Table**), which showed a small difference attributed to basis set superposition error (RMSE = 0.6 kcal/mol). As a result, all calculations were done with def2-TZVP.

Table S22. Comparison of different implicit solvent models and size of the basis set for DFT calculations of ***reaction 1****.*

	CPCM(DCM)-M06-2X/def2-TZVP//CPCM(DCM)-PBE0-D3BJ/def2-SVP	SMD(DCM)-M06-2X/def2-TZVP	CPCM(DCM)-M06-2X/def2-QZVP
	ΔE [kcal mol^{-1}]	ΔE [kcal mol^{-1}]	ΔE [kcal mol^{-1}]
RS (Nu1− + E)	0	0.0	0.0
RC (Nu1− · E)	-3.4	-3.2	-2.8
TS	6.7	6.3	7.2
PS	-2.4	-4.3	-1.8
	MAE	0.9	0.5
	RMSE	1.1	0.6

S12.1.3Catalysed reaction

To determine the dynamic behaviour of the cage in solution, including its ability to coordinate solvent molecules prior to the reaction, we performed a 3×100 ns MD simulation for a solvated cubic box containing 500 DCM and 17 water molecules as well as the substrates ($Nu1^-$ and E), cage, and H^+DBU. The starting configuration was obtained by placing molecules randomly in the simulation box.

The simulations revealed that $Nu1^-$ predominantly resides in the cage during the simulation time. This was confirmed by visual inspection and analysis of the COM distance between the substrates and the cage (53 %; **Figure a**). Sporadically, $Nu1^-$ is also observed on the interface between the cavity and the bulk solvent, as seen by the small second peak in distance distribution (**Figure a,b**). Moreover, the binding of $Nu1^-$ within the cage is usually accompanied by the coordination of 1−2 water molecules or one DCM molecule within the cage (**Figure c**). In contrast, E remained outside of the cage.

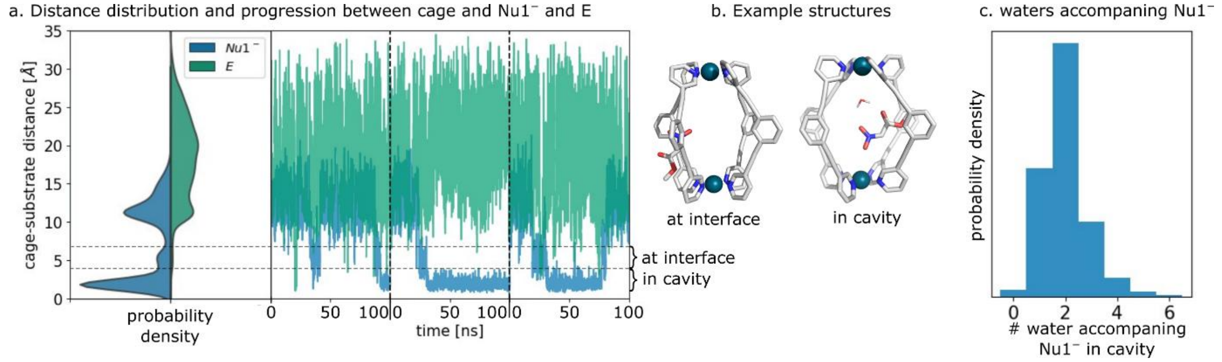

Figure S99. (a) The distribution and progression of the distance between centres of masses of cage and $Nu1^-$ (blue) and cage and E (green) from 3x100 ns MD simulations. (b) Example structures of $Nu1^-$ on the interface and in the cavity extracted from the MD simulations. (c) Histogram of the number of water molecules accompanying $Nu1^-$ inside the cavity.

Representative frames from MD simulations of the **$Nu1^-$ · DCM⊂C**, **$Nu1^-$ · H_2O⊂C**, and **$Nu1^-$ ·** 2 **H_2O⊂C** complexes were extracted and subjected to QM calculations. Additionally, the **$Nu1^-$ · E⊂C** complex was obtained from DFT optimisation by following reactive coordinate from the TS structure towards reactants (see methods). A comparison of the four complexes revealed that the **$Nu1^-$ · DCM⊂C** is higher energy than the others (> 3 kcal mol^{-1}), while the differences in energies among the last three complexes are small (<

1.1 kcal mol^{-1}), with **Nu1⁻ · H₂O** having lowest energy (**Figure**). The Boltzmann distribution obtained from free energies shows a population ratio of 0:1:0.4:0.2, suggesting that the last three configurations, **Nu1⁻ · H₂O⊂C**, **Nu1⁻ ·** 2 **H₂O⊂C**, and **Nu1⁻ · E⊂C,** would co-exist in solution.

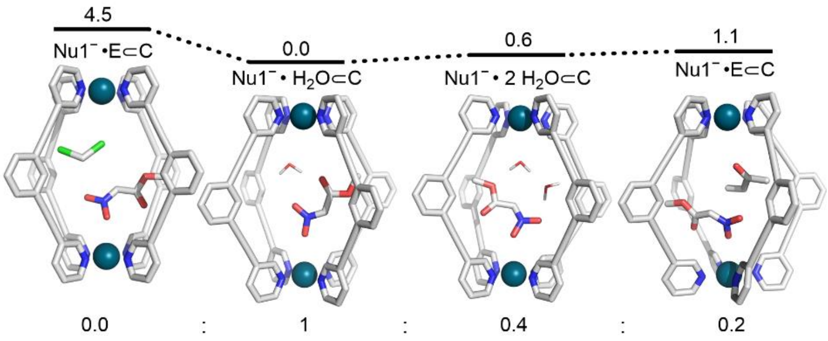

*Figure S100. Free energies and Boltzmann rations of the four complexes, **Nu1⁻ · DCM⊂C**, **Nu1⁻ · H₂O⊂C**, **Nu1⁻ ·** 2 **H₂O⊂C**, and **Nu1⁻ · E⊂C** from DFT calculations.*

Interestingly, while the free energies of **Nu1⁻ · H₂O⊂C**, **Nu1⁻ ·** 2 **H₂O⊂C**, and **Nu1⁻ · E⊂C** complexes are similar, their enthalpic-entropic contributions significantly differ (**Table**). Relative to **Nu1⁻ · H₂O**, the two other complexes **Nu1⁻ · E⊂C** and **Nu1⁻ ·** 2**H₂O⊂C** are enthalpically more stabilised, which is compensated by their unfavourable entropic contributions.

*Table S23. Energies of found reactant states for **reaction 1** with the cage. The effective reactant state is calculated as their Boltzmann average of Nu1⁻ · H₂O⊂C, Nu1⁻ · 2 H₂O⊂C and Nu1⁻ · E⊂C. Calculations on CPCM(DCM)-M06-2X/def2-TZVP//CPCM(DCM)-PBE0-D3BJ/def2-SVP level of theory.*

	ΔH [kcal mol^{-1}]	−TΔS [kcal mol^{-1}]	ΔG [kcal mol^{-1}]	Boltzmann factor	probability
Nu1– · DCM⊂C	2.5	1.8	4.3	0.00	0.00
Nu1– · H2O⊂C	2.1	-2.4	-0.3	1.64	0.65
Nu1– · 2 H2O⊂C	-5.1	5.4	0.3	0.60	0.24
Nu1– · E⊂C	-0.7	1.5	0.8	0.27	0.11
effective reactive state (Boltzmann average)	0	0	0		
TS	10.4	3.6	14.0		
PS	-0.3	3.9	3.6		

The cage-catalysed free energy barrier ($\Delta G^{\ddagger}$=14.0 kcal mol^{-1}) was calculated relative to the effective reactive state, i.e., the Boltzmann weighted energy of the three complexes: **Nu1⁻ · H₂O⊂C**, **Nu1⁻ ·** 2 **H₂O⊂C**, and **Nu1⁻ · E⊂C** (**Table**). This value is significantly lower than the cage-free reaction ($\Delta G^{\ddagger}$= 19.2 kcal mol^{-1}), mainly due to a significant decrease in entropy.

The TS structure inside the cage is similar to the one obtained for the cage-free reaction. A late TS is obtained in both cases, with a C-C distance of 2.05/2.04 Å inside the cage and solution, respectively. To understand the origin of the catalytic activity, we performed a distortion/interaction (D/I) analysis.[32] Here, relative electronic energies of separate species Nu1⁻, E (and cage) were calculated in the geometry of TS relative to their geometries in ground states. Typically, D/I analysis is performed for reactions along selected reactive coordinate(s), and as a result, the reactant and transition states consist of the same molecules. Unfortunately, the D/I analysis could not be applied directly because the composition of the

structures with the lowest energy and their respective TSs are different for both cage-free and cage-catalysed reactions. Therefore, we perform the D/I analysis for a part of the entire process: the transformation of the reactant complexes, **Nu1⁻ · E** and **Nu1– · E⊂C,** into their respective TSs (**Figure a**). While such a comparison is insightful, it is essential to note that this is only a fragment of the process.

The analysis reveals that the confinement of the cage results in higher distortion energy of the **Nu1⁻ · E** complex inside the cage compared to the solution ($\Delta\Delta E^{\ddagger}_{strain}$ = 2.8 kcal mol^{-1}; **Figure b**). However, this increase in energy is partially compensated by a stronger interaction energy inside the cage ($\Delta\Delta E^{\ddagger}_{int}$ = −1.6 kcal mol^{-1}). Consequently, this leads to an electronic activation barrier, which is 1.2 kcal mol^{-1} higher inside the cage. This suggests that catalysis originates from entropic contributions rather than the electronic stabilisation of TS.

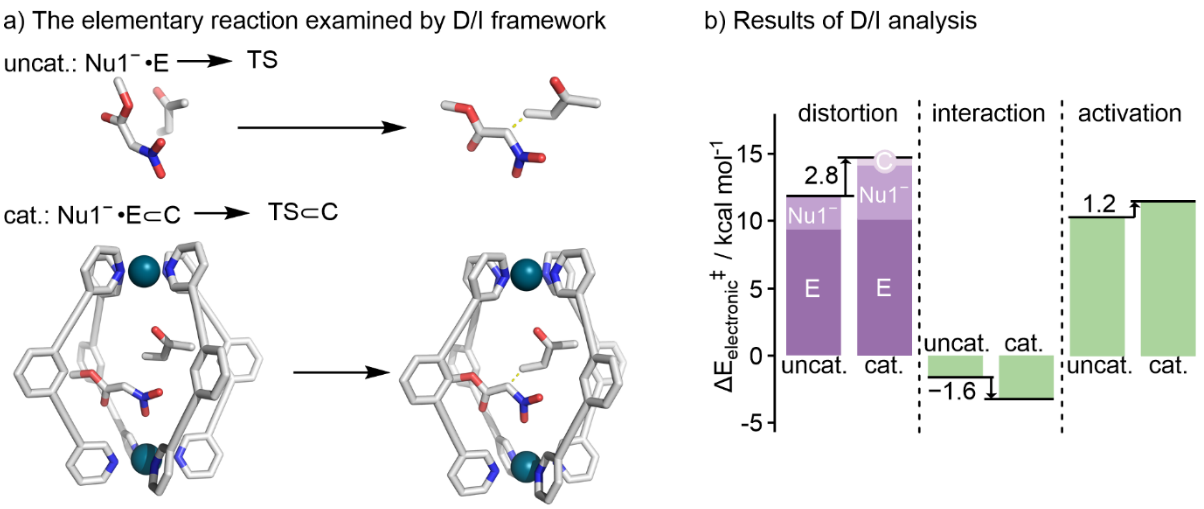

*Figure S101. D/I analysis of **reaction 1** fragment with cage and cage-free. (a) The studied reaction fragment starts from the Nu1–E reactant complex to TS with the cage and cage-free. (b) Results of D/I analysis.*

S12.2 Reaction 2

S12.2.1Cage-free reaction

Reaction 2 was analysed using the same methodology as described for the reaction above. The activation energy of the reaction is 18.3 kcal mol^{-1}(**Figure** ; **Table**), similar to the first reaction. Again, to reach the transition state, both substrates must form a reactive complex, which requires 6.9 kcal/mol (~40 % of the total activation barrier). Similar to **reaction 1**, the CPCM(DCM)-M06-2X/def-TZVP level of theory was validated against the CPCM-DLPNO-CCSD(T)/def2-TZVPP reference method (**Table**).

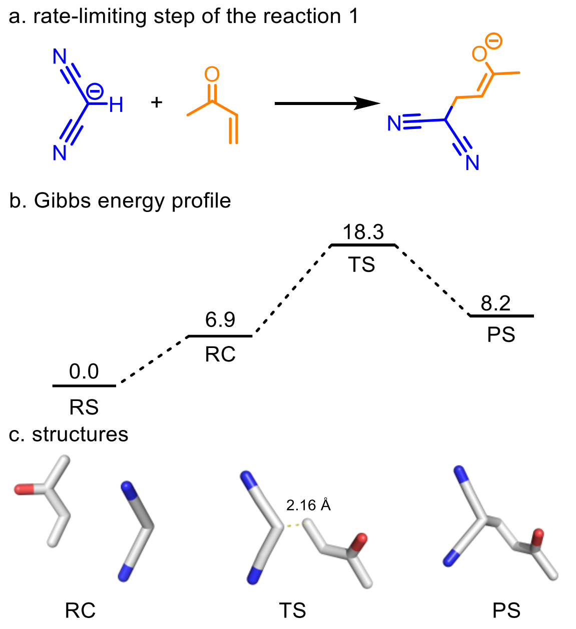

*Figure S102. (a) Gibbs free energy profile of the C-C formation in **reaction 2** in DCM. (b) Optimised structures of reactant complex, transition state, and product. Calculations on CPCM(DCM)-M06-2X/def2-TZVP//CPCM(DCM)-PBE0-D3BJ/def2-SVP level of theory.*

*Table S24. QM calculations of cage-free **reaction 2** and comparison between different levels of theories. The geometry optimisations and thermal contributions were calculated on the CPCM(DCM)-PBE0-D3BJ/def2-SVP level of theory.*

	CPCM-DLPNO-CCSD(T)/def2-TZVPP	CPCM-wB97X/def2-TZVP	CPCM(DCM)-M06-2X/def2-TZVP			
	ΔE [kcal mol^{-1}]	ΔE [kcal mol^{-1}]	ΔE [kcal mol^{-1}]	ΔH [kcal mol^{-1}]	−TΔS [kcal mol^{-1}]	ΔG [kcal mol^{-1}]
RS (Nu2− + E)	0	0	0	0	0	0
RC (Nu2− · E)	-3.1	-3.3	-2.8	-1.5	8.4	6.9
TS	9.0	10.3	7.2	8.0	10.3	18.3
PS	-7.6	-5.0	-5.0	-3.1	11.3	8.2
	MAE	1.4	1.6			
	RMSE	1.7	1.9			

MD simulations. We performed a 3×100 ns MD simulation of the Nu2$^-$, E, cage, H$^+$DBU and 17 water molecules in a cubic box with explicit DCM similar to **reaction 1**. The Nu2$^-$ spends considerable time inside the cage (**Figure a**). In contrast to Nu1$^-$, Nu2$^-$ spends significant time at the interface of the cage, as seen

by the large height of the second peak in distance distribution (**Figure a,b**). Moreover, the amount of water accompanying the Nu2⁻ inside the cage is larger than for Nu1⁻ (3–4 water molecules; **Figure c**).

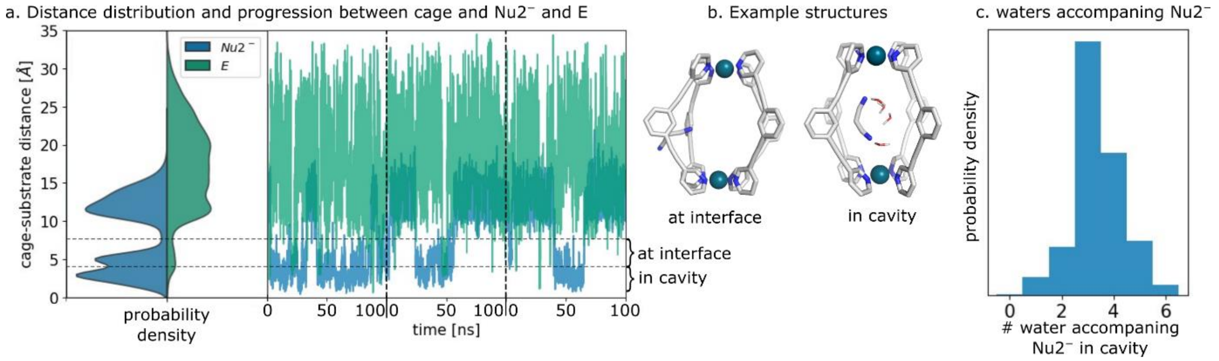

Figure S103. (a) The distribution and progression of the distance between centres of masses of cage and Nu2⁻ (blue), and cage and E (green) from 3x100 ns MD simulations. (b) Example structures of Nu2⁻ on the interface and in the cavity extracted from the MD simulations. (c) Histogram of the number of water molecules accompanying Nu2⁻ inside the cavity.

The resultant MD trajectories reveal the dynamic behaviour of Nu2⁻ bound in a cage, which frequently changes orientations and positions. This contrasts with the results for **reaction 1**, where the MD trajectory showed only a couple of persisting binding modes. To describe the conformational space of Nu2⁻ inside the cage, we used two collective variables (**Figure a**):

a) the distance between CoMs of the cage and Nu2⁻, which indicates the relative Nu2⁻ position to the cage,
b) the angle between the vector connecting the two cage's palladium atoms and the vector of Nu2⁻ from the central carbon to hydrogen, indicating the molecule's orientation relative to the cage.

The resultant space was clustered using agglomerative clustering (implemented in the scikit-learn package[33]) with 30 clusters (**Figure b**). A representative structure was extracted for each cluster with water molecules directly connected to the Nu2⁻, from which four (with 2–4 water molecules) were selected for further DFT optimisation (**Figure c**). The energies of the obtained structures were > 4 kcal mol^{-1} higher than the reactant complex inside the cage (**Table**).

The higher energy of complexes with water might be due to overestimating translational entropy contributions for reactions in which molecules are released,[33,34] especially affecting the cage with four water molecules. To check if this is the case, we have reduced the configurations to contain only single water molecules, **Nu2⁻ · H_2O⊂C,** matching the molarity of **Nu2⁻ · E⊂C**. Even after this manipulation, the resultant free energies of **Nu2⁻ · H_2O⊂C** complexes were higher than **Nu2⁻ · E⊂C** (but with a smaller difference ~1 kcal mol^{-1}; **Table**), which supports that the **Nu2⁻ · E⊂C** complex is in the energetic minimum.

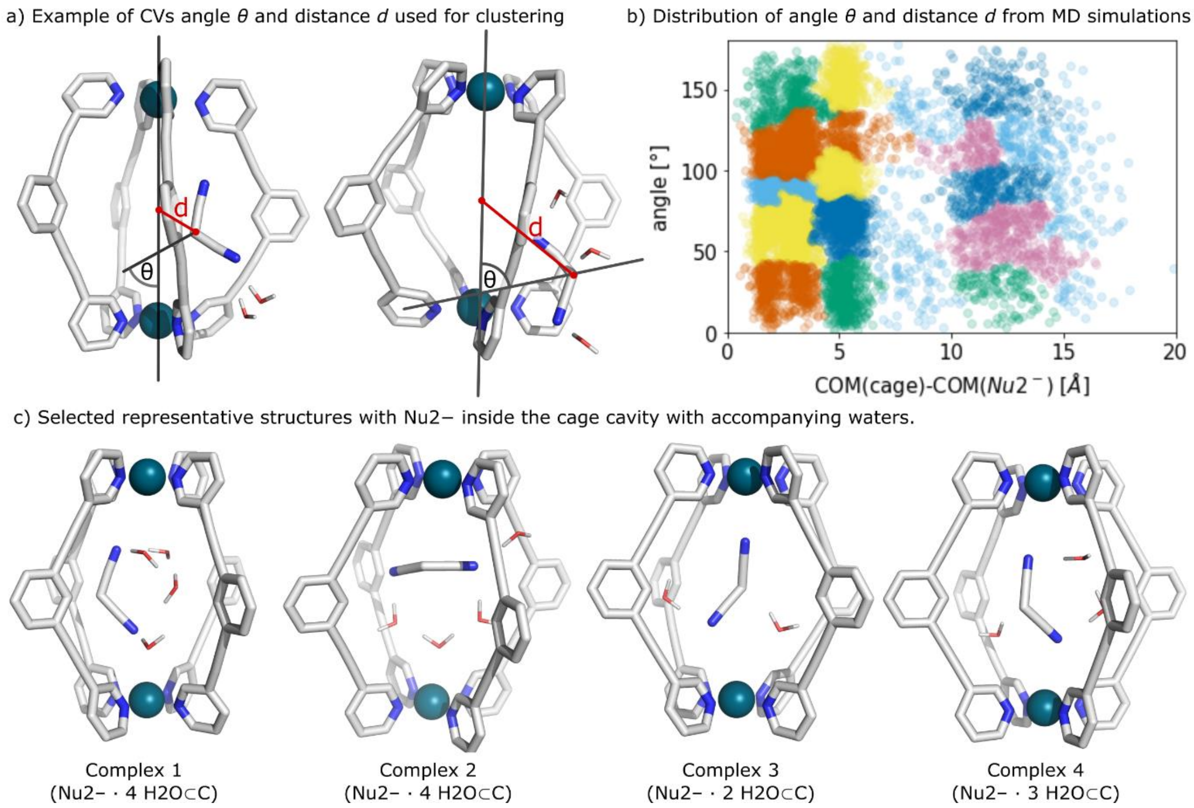

Figure S104. Analysis of conformational space of ***reaction 2*** *based on MD trajectory. (a) Collective variables used for analysis: distance between the centre of masses of molecule and cage, and angle between the Nu2− and the cage (black). (b) scatterplot of the values of angle and the distance obtained from MD trajectories. Different colours represent the membership to the cluster. (c) Selected frames of Nu2⁻ inside the cage accompanied by waters.*

S12.2.2Catalysed reaction

The catalysis inside the cage has been analysed similarly to **reaction 1**: the product was bound using *cgbind,* and the transition state and reactant complex were found using a one-dimensional scan along the formed bond. The activation barrier was calculated relative to the effective reactant state consisting of the two lowest energy complexes, **Nu2⁻ · E⊂C** and **Nu2⁻ · H_2O⊂C**, weighted by their Boltzmann probabilities (**Table**). The obtained activation barrier ($\Delta G^{\ddagger}$=13.8 kcal mol^{-1}; **Table** Error! Not a valid bookmark self-reference.) shows a significant decrease in height to cage-free reaction ($\Delta G^{\ddagger}$=18.2 kcal mol^{-1}), with a considerable energy reduction originating from entropic contribution.

Table S25. QM analysis of the ***reaction 2*** *MA of Nu2⁻ and E inside the cage. The effective reactant state is calculated as their Boltzmann average of Nu2⁻ · H_2O⊂C and Nu1⁻ · E⊂C. Calculations on CPCM(DCM)-M06-2X/def2-TZVP//CPCM(DCM)-PBE0-D3BJ/def2-SVP level of theory.*

	ΔH [kcal mol^{-1}]	−TΔS [kcal mol^{-1}]	ΔG [kcal mol^{-1}]	probability
Nu2– · H2O⊂C	2.6	-2.2	0.4	0.29
Nu2– · E⊂C	-1.1	0.9	-0.2	0.71
Effective ground state (Boltzmann average)	0.0	0.0	0.0	
TS	9.6	4.2	13.8	
PS	-3.5	4.3	0.8	

Absolute energies

Table S26. Single point calculation and thermal contributions for the systems studied in this work.

	Optimisation level of theory	CPCM(DCM)-PBE0-D3BJ/def2-SVP								
	Single point level of theory	CPCM(DCM)-PBE0-D3BJ/def2-SVP				CPCM(DCM)-M06-2X/def2-TZVP	CPCM(DCM)-DLPNO-CCSD(T)/def2-TZVPP	CPCM(DCM)-ωB97X/def2-TZVP	CPCM(DCM)-M06-2X/def2-QZVP	SMD(DCM)-M06-2X/def2-TZVP
		E [Eh]	H-E [Eh]	G-E [Eh]	Im. freq. [cm^{-1}]	E [Eh]	E [Eh]	E [Eh]	E [Eh]	E [Eh]
	DCM	-959.0372	0.0339	0.0058	None	-959.6997				
	H_2O	-76.2853	0.0252	0.0067	None	-76.4358				
	Nu1⁻	-471.5603	0.0899	0.0516	None	-472.4308	-471.7333	-472.4972	-472.4692	-472.421953
	Nu2–	-224.0793	0.0374	0.0080	None	-224.5199	-224.1364	-224.5363	-224.538102	-224.5173748
	E	-959.0372	0.0962	0.0636	None	-231.2251	-230.8542	-231.2680	-231.2462	-231.2271483
Reaction 1	1 (Nu1– · 4 H2O)	-776.7767	0.2016	0.1401	None	-778.2157				
	2 (Nu1– · 4 H2O)	-776.7900	0.2028	0.1443	None	-778.2196				
	3 (Nu1– · 4 H2O)	-776.7859	0.2026	0.1433	None	-778.2193				
	4 (Nu1– · 4 H2O)	-776.7788	0.2017	0.1400	None	-778.2168				
	cage-free									
	RC	-702.3772	0.1883	0.1333	None	-703.6614	-702.5944	-703.7699	-703.7199	-703.6541748
	TS	-702.3670	0.1877	0.1351	-347.85	-703.6452	-702.5752	-703.7492	-703.7038	-703.6389913
	PS	-702.3820	0.1896	0.1377	None	-703.6597	-702.5963	-703.7690	-703.7183	-703.6559736
	with cage									
	Nu1– · DCM⊂C	-5194.6026	1.2662	1.0683	-3.14	-5202.2102				
	Nu1– · H2O⊂C	-4311.8490	1.2578	1.0629	None	-4318.9473				
	Nu1– · 2 H2O⊂C	-4388.1654	1.2861	1.0852	-2.06	-4395.3978				
	Nu1– · E⊂C	-4466.3726	1.3289	1.1261	None	-4473.7412				-4473.818521
	TS	-4466.3636	1.3284	1.1289	-358.02	-4473.7231				-4473.8003
	PS	-4466.3794	1.3310	1.1321	None	-4473.7426				
	D/I analysis									
	Nu1⁻ in geometry of Nu1– · E					-472.428532				
	Nu1⁻ in geometry of TS(Nu1– · E)					-472.424737				
	Nu1⁻ in geometry of Nu1– · E⊂C					-472.430416				
	Nu1⁻ in geometry of TS(Nu1– · E⊂C)					-472.424102				
	E in geometry of Nu1– · E					-231.224862				
	E in geometry of TS(Nu1– · E)					-231.209913				
	E in geometry of Nu1– · E⊂C					-231.224783				
	E in geometry of TS(Nu1– · E⊂C)					-231.208745				
	C in geometry of Nu1– · E⊂C					-3770.00774				
	C in geometry of TS(Nu1– · E⊂C)					-3770.00684				
Re	cage-free									

action 2	RC	-454.8940	0.1356	0.0870	None	-455.7495	-454.9954	-455.8095	-455.788382	-455.7481284
	TS	-454.8811	0.1350	0.0893	-335.41	-455.7336	-454.9762	-455.7878	-455.772753	-455.7307932
	PS	-454.9016	0.1365	0.0926	None	-455.7530	-455.0027	-455.8122	-455.792264	-455.7499833
	with cage									
	Nu2– · E⊂C	-4218.8706	1.2756	1.0760	-4.11	-4225.8177				
	Complex (various number of water molecules)									
	1 (Nu2– · 4 H2O⊂C)	-4293.2670	1.2889	1.0850	-	-4300.3600				
	2 (Nu2– · 4 H2O⊂C)	-4293.2688	1.2881	1.0822	-26.79	-4300.3648				
	3 (Nu2– · 2 H2O⊂C)	-4140.6447	1.2320	1.0338	-18.84	-4147.4646				
	4 (Nu2– · 3 H2O⊂C)	-4216.9553	1.2608	1.0612	-	-4223.9097				
	Truncated to single water molecules									
	1 (Nu2– · H2O⊂C)	-4064.3482	1.2049	1.0161	-	-4071.0232				
	2 (Nu2– · H2O⊂C)	-4064.3393	1.2045	1.0145	-4.67	-4071.0173				
	3 (Nu2– · H2O⊂C)	-4064.3445	1.2046	1.0141	None	-4071.0225				
	4 (Nu2– · H2O⊂C)	-4064.3480	1.2051	1.0163	-	-4071.0230				
	TS	-4218.85935	1.2753	1.0808	-294.16, -1.60	-4225.8003				
	PS	-4218.8796	1.2780	1.0837	-3.84	-4225.8239				

S13. References

(1) August, D. P.; Nichol, G. S.; Lusby, P. J. Maximizing Coordination Capsule-Guest Polar Interactions in Apolar Solvents Reveals Significant Binding. *Angew. Chemie Int. Ed.* **2016**, *55* (48), 15022–15026. https://doi.org/10.1002/anie.201608229.

(2) Neese, F.; Wennmohs, F.; Becker, U.; Riplinger, C. The ORCA Quantum Chemistry Program Package. *J. Chem. Phys.* **2020**, *152* (22). https://doi.org/10.1063/5.0004608.

(3) Neese, F. Software Update: The ORCA Program System—Version 5.0. *Wiley Interdiscip. Rev. Comput. Mol. Sci.* **2022**, *12* (5), 1–15. https://doi.org/10.1002/wcms.1606.

(4) Adamo, C.; Barone, V. Toward Reliable Density Functional Methods without Adjustable Parameters: The PBE0 Model. *J. Chem. Phys.* **1999**, *110* (13), 6158–6170. https://doi.org/10.1063/1.478522.

(5) Weigend, F.; Ahlrichs, R. Balanced Basis Sets of Split Valence, Triple Zeta Valence and Quadruple Zeta Valence Quality for H to Rn: Design and Assessment of Accuracy. *Phys. Chem. Chem. Phys.* **2005**, *7* (18), 3297–3305. https://doi.org/10.1039/b508541a.

(6) Grimme, S.; Antony, J.; Ehrlich, S.; Krieg, H. A Consistent and Accurate Ab Initio Parametrization of Density Functional Dispersion Correction (DFT-D) for the 94 Elements H-Pu. *J. Chem. Phys.* **2010**, *132* (15), 1–19. https://doi.org/10.1063/1.3382344.

(7) Grimme, S.; Ehrlich, S.; Goerigk, L. Effect of the Damping Function in Dispersion Corrected Density Functional Theory. *J. Comput. Chem.* **2011**, *32* (7), 1456–1465. https://doi.org/10.1002/jcc.

(8) Zhao, Y.; Truhlar, D. G. The M06 Suite of Density Functionals for Main Group Thermochemistry, Thermochemical Kinetics, Noncovalent Interactions, Excited States, and Transition Elements: Two New Functionals and Systematic Testing of Four M06-Class Functionals and 12 Other Function. *Theor. Chem. Acc.* **2008**, *120* (1–3), 215–241. https://doi.org/10.1007/s00214-007-0310-x.

(9) Sure, R.; Grimme, S. Comprehensive Benchmark of Association (Free) Energies of Realistic Host-Guest Complexes. *J. Chem. Theory Comput.* **2015**, *11* (8), 3785–3801. https://doi.org/10.1021/acs.jctc.5b00296.

(10) Barone, V.; Cossi, M. Conductor Solvent Model. *J. Phys. Chem. A* **1998**, *102* (97), 1995–2001.

(11) Grimme, S. Supramolecular Binding Thermodynamics by Dispersion-Corrected Density Functional Theory. *Chem. Eur. J.* **2012**, *18* (32), 9955–9964. https://doi.org/10.1002/chem.201200497.

(12) Young, T. A.; Silcock, J. J.; Sterling, A. J.; Duarte, F. AutodE: Automated Calculation of Reaction Energy Profiles— Application to Organic and Organometallic Reactions. *Angew. Chem. Int. Ed.* **2021**, *60* (8), 4266–4274. https://doi.org/https://doi.org/10.1002/anie.202011941.

(13) Young, T. A.; Gheorghe, R.; Duarte, F. Cgbind: A Python Module and Web App for Automated Metallocage Construction and Host–Guest Characterisation. *J. Chem. Inf. Model.* **2020**, *60* (7), 3546–3557. https://doi.org/10.1021/acs.jcim.0c00519.

(14) Abraham, M. J.; Murtola, T.; Schulz, R.; Pall, S.; Smith, J. C.; Hess, B.; Lindah, E. Gromacs: High Performance Molecular Simulations through Multi-Level Parallelism from Laptops to Supercomputers. *SoftwareX* **2015**, *1–2*, 19–25. https://doi.org/10.1016/j.softx.2015.06.001.

(15) Van Der Spoel, D.; Lindahl, E.; Hess, B.; Groenhof, G.; Mark, A. E.; Berendsen, H. J. C. GROMACS: Fast, Flexible, and Free. *J. Comput. Chem.* **2005**, *26* (16), 1701–1718. https://doi.org/10.1002/jcc.20291.

(16) van der Spoel, D.; van Maaren, P. J.; Caleman, C. GROMACS Molecule & Liquid Database. *Bioinformatics* **2012**, *28* (5), 752–753. https://doi.org/10.1093/bioinformatics/bts020.

(17) Izadi, S.; Anandakrishnan, R.; Onufriev, A. V. Building Water Models : A Different Approach. *J. Phys. Chem. Lett.* **2014**, *5*, 3853–3871.

(18) Berendsen, H. J. C.; Postma, J. P. M.; van Gunsteren, W. F.; DiNola, A.; Haak, J. R. Molecular Dynamics with Coupling to an External Bath. *J. Chem. Phys.* **1984**, *81* (8), 3684–3690. https://doi.org/10.1063/1.448118.

(19) Bussi, G.; Donadio, D.; Parrinello, M. Canonical Sampling through Velocity Rescaling. *J. Chem. Phys.* **2007**, *126* (2007), 014101. https://doi.org/10.1063/1.2408420.

(20) Parrinello, M.; Rahman, A. Crystal Structure and Pair Potentials: A Molecular-Dynamics Study. *Phys. Rev. Lett.* **1980**, *45* (14), 1196–1199. https://doi.org/10.1103/PhysRevLett.45.1196.

(21) Darden, T.; York, D.; Pedersen, L. Particle Mesh Ewald: An N -Log(N) Method for Ewald Sums in Large Systems. *J. Chern. Phys.* **1993**, *98* (12), 10089–10092.

(22) Essmann, U.; Perera, L.; Berkowitz, M. L.; Darden, T.; Lee, H.; Pedersen, L. G. A Smooth Particle Mesh Ewald Method. *J. Chem. Phys.* **1995**, *103* (19), 8577–8593.

https://doi.org/10.1063/1.470117.

(23) Michaud-Agrawal, N.; Denning, E. J.; Woolf, T. B.; Beckstein, O. MDAnalysis: A Toolkit for the Analysis of Molecular Dynamics Simulations. *J. Comput. Chem.* **2011**, *32* (10), 2319–2327. https://doi.org/10.1002/jcc.

(24) Gowers, R. J.; Linke, M.; Barnoud, J.; Reddy, T. J. E.; Melo, M. N.; Seyler, S. L.; Domański, J.; Dotson, D. L.; Buchoux, S.; Kenney, I. M.; Beckstein, O. MDAnalysis: A Python Package for the Rapid Analysis of Molecular Dynamics Simulations. In *Proceedings of the 15th Python in Science Conference*; Benthall, S., Rostrup, S., Eds.; 2016; pp 98–105. https://doi.org/10.25080/Majora-629e541a-00e.

(25) Wang, J.; Wolf, R. M.; Caldwell, J. W.; Kollman, P. A.; Case, D. A. Development and Testing of a General Amber Force Field. *J. Comput. Chem.* **2004**, *25* (9), 1157–1174. https://doi.org/10.1002/jcc.20035.

(26) Li, P.; Merz, K. M. MCPB.Py: A Python Based Metal Center Parameter Builder. *J. Chem. Inf. Model.* **2016**, *56* (4), 599–604. https://doi.org/10.1021/acs.jcim.5b00674.

(27) Allen, A. E. A.; Payne, M. C.; Cole, D. J. Harmonic Force Constants for Molecular Mechanics Force Fields via Hessian Matrix Projection. *J. Chem. Theory Comput.* **2018**, *14* (1), 274–281. https://doi.org/10.1021/acs.jctc.7b00785.

(28) Tafi, A.; Agamennone, M.; Tortorella, P.; Alcaro, S.; Gallina, C.; Botta, M. AMBER Force Field Implementation of the Boronate Function to Simulate the Inhibition of β-Lactamases by Alkyl and Aryl Boronic Acids. *Eur. J. Med. Chem.* **2005**, *40* (11), 1134–1142. https://doi.org/10.1016/j.ejmech.2005.06.011.

(29) Goodman, J. M.; Paterson, I.; Kahn, S. S. A Force Field Model for Boron Enolates. *Tetrahedron Lett.* **1987**, *28* (43), 5209–5212. https://doi.org/10.1016/S0040-4039(00)95631-4.

(30) Bayly, C. I.; Cieplak, P.; Cornell, W. D.; Kollman, P. A. A Well-Behaved Electrostatic Potential Based Method Using Charge Restraints for Deriving Atomic Charges: The RESP Model. *J. Phys. Chem.* **1993**, *97* (40), 10269–10280. https://doi.org/10.1021/j100142a004.

(31) Young, T. A.; Martí-Centelles, V.; Wang, J.; Lusby, P. J.; Duarte, F. Rationalising the Activity of an “Artificial Diels-Alderase”: Establishing Efficient and Accurate Protocols for Calculating Supramolecular Catalysis. *J. Am. Chem. Soc.* **2020**, *142* (3), 1300–1310. https://doi.org/10.1021/jacs.9b10302.

(32) Bickelhaupt, F. M.; Houk, K. N. Analysing Reaction Rates with the Distortion/Interaction-Activation Strain Model. *Angew. Chem. Int. Ed.* **2017**, *56* (34), 10070–10086. https://doi.org/10.1002/anie.201701486.

(33) Contreras-García, J.; Johnson, E. R.; Keinan, S.; Chaudret, R.; Piquemal, J. P.; Beratan, D. N.; Yang, W. NCIPLOT: A Program for Plotting Noncovalent Interaction Regions. *J. Chem. Theory Comput.* **2011**, *7* (3), 625–632. https://doi.org/10.1021/ct100641a.

(34) Pedregosa, F.; Varoquaux, G.; Gramfort, A.; Michel, V.; Thirion, B.; Grisel, O.; Blondel, M.; Prettenhofer, P.; Weiss, R.; Dubourg, V.; Vanderplas, J.; Passos, A.; Cournapeau, D.; Brucher, M.; Perrot, M.; Duchesnay, E. Scikit-Learn: Machine Learning in Python. *J. Mach. Learn. Res.* **2011**, *12*, 2825–2830. https://doi.org/10.1145/2786984.2786995.

(35) Ariai, J.; Gellrich, U. The Entropic Penalty for Associative Reactions and Their Physical Treatment

during Routine Computations. *Phys. Chem. Chem. Phys.* **2023**, *25* (20), 14005–14015. https://doi.org/10.1039/d3cp00970j.
